# Modulation of Immune Signaling and Metabolism Highlights Host and Fungal Transcriptional Responses in Mouse Models of Invasive Pulmonary Aspergillosis

**DOI:** 10.1038/s41598-017-17000-1

**Published:** 2017-12-06

**Authors:** Shiv D. Kale, Tariq Ayubi, Dawoon Chung, Nuria Tubau-Juni, Andrew Leber, Ha X. Dang, Saikumar Karyala, Raquel Hontecillas, Christopher B. Lawrence, Robert A. Cramer, Josep Bassaganya-Riera

**Affiliations:** 1 Nutrional Immunology and Molecular Medicine Laboratory, Biocomplexity Institute of Virginia Tech., Blacksburg, VA 24061 USA; 2 Department of Microbiology and Immunology, Geisel School of Medicine at Dartmouth, Hanover, NH 03755 USA; 3 Department of Biological Sciences, Virginia Tech., Blacksburg, VA 24061 USA; 4 Present Address: McDonnell Genome Institute at Washington University, St. Louis, MO 63108 USA; 5 Present Address: National Marine Biodiversity Institute of Korea, Seochun-gun, 33662 Republic of Korea

## Abstract

Incidences of invasive pulmonary aspergillosis, an infection caused predominantly by *Aspergillus fumigatus*, have increased due to the growing number of immunocompromised individuals. While *A. fumigatus* is reliant upon deficiencies in the host to facilitate invasive disease, the distinct mechanisms that govern the host-pathogen interaction remain enigmatic, particularly in the context of distinct immune modulating therapies. To gain insights into these mechanisms, RNA-Seq technology was utilized to sequence RNA derived from lungs of 2 clinically relevant, but immunologically distinct murine models of IPA on days 2 and 3 post inoculation when infection is established and active disease present. Our findings identify notable differences in host gene expression between the chemotherapeutic and steroid models at the interface of immunity and metabolism. RT-qPCR verified model specific and nonspecific expression of 23 immune-associated genes. Deep sequencing facilitated identification of highly expressed fungal genes. We utilized sequence similarity and gene expression to categorize the *A. fumigatus* putative *in vivo* secretome. RT-qPCR suggests model specific gene expression for nine putative fungal secreted proteins. Our analysis identifies contrasting responses by the host and fungus from day 2 to 3 between the two models. These differences may help tailor the identification, development, and deployment of host- and/or fungal-targeted therapeutics.

## Introduction

Invasive pulmonary aspergillosis (IPA) is an infection of the lower respiratory system by the filamentous fungus *Aspergillus fumigatus*, and is principally associated with high mortality rates. IPA occurs in immune compromised patient populations and progresses rapidly. These populations are composed of those: (i) suffering from severe or prolonged neutropenia^1,2^ (ii) receiving prolonged and high dose steroid treatments^3,4^ (iii) receiving immune suppressive regimens^5^, (iv) receiving stem cell and organ transplants^6–8^, (v) with chronic obstructive pulmonary disease (COPD), and (vi) viral and microbial sepsis^9–11^. The spectrum and diversity of patients susceptible to IPA is rather astounding though the underlying mechanisms in each at risk patient population remains enigmatic. Progression of IPA is thought to be dependent primarily on the type and severity of immune deficiency and includes both quantitative and qualitative innate immune effector cell defects. However, recent studies also highlight significant fungal strain variability that may account for differences in establishment of infection and disease progression^12–16^. Several mouse models of IPA have been developed to dissect clinically relevant host and fungal responses. For example, IPA in chemotherapeutic mouse models, highlighted by the use of cyclophosphamide, is thought to be due to host damage driven principally by the growth and progression of the fungus. Host damage in repeated high dose cortisone acetate treatment models has been suggested to occur due to a combination of fungal proliferation and immunopathogenesis^17,18^. Pulmonary cytokine responses differ amongst mouse models of IPA^19^, as well as putative fungal virulence mechanisms, with the role of gliotoxin being a prime example^18,20^.

The development of whole genome and transcript based sequencing technologies has facilitated the discovery of novel aspects of *A. fumigatus* biology and pathogenesis. Genome sequences are available for several strains, including both the Af293 and Af1163 isolates^21,22^. Transcriptomics studies focused on *A. fumigatus* biofilm and planktonic growth provided novel insight into newly identified genes associated with biofilm formation^23^. *In vitro* challenge of *A. fumigatus* conidia and hyphae with neutrophils from humans suggested enhanced metabolic reprogramming and iron/copper assimilation in response to healthy neutrophils in comparison to those suffering from chronic granulomatous disease^24^. In human blood, *A. fumigatus* is thought to enter a resting mycelial stage due to decreased expression of genes associated with metabolism and nutrient uptake^25^. Dual organism transcriptomics of human airway epithelial cells challenged with *A. fumigatus* has also provided mechanistic insights into differences between immortalized and primary cell responses to *A. fumigatus in vitro* ^26^. Transcriptomics of normal human monocytes in response to *A. fumigatus* identified several upregulated cytokines, specifically IL-1β, IL-8, CXCL2, CCL4, CCL3, and CCL20^27^. Transcriptomics has also facilitated the identification of global gene expression changes associated with the pH-responsive transcription factor PacC during chemotherapeutic mouse model of IPA^28^. *In vivo* transcriptomics studies of *A. fumigatus* identified SrbA as a novel regulator of fungal hypoxia and virulence^29^. Importantly, it seems clear that *in vivo* transcriptional responses are likely different from standard *in vitro* culture conditions as highlighted by studies on the AcuK and AcuM transcription factors^30^.

Here we provide a global overview of our dual organism transcriptomics study aimed at identifying differences and similarities in host and fungal gene expression between steroid treatment and chemotherapeutic mouse models of IPA. Our findings highlight the novel and context-specific expression of several *Nlrs*, *Tlrs*, and *Clecs* during IPA. We also identify conserved and contrasting expression of the putative *A. fumigatus* secretome between the chemotherapeutic and steroid mouse models of IPA. These differences and similarities in host and fungal gene expression provide a system-wide overview of the interaction of *A. fumigatus* and the host. Determination of global gene expression profiles during chemotherapeutic and steroid models of IPA provides an important framework for the system-wide identification of potential novel host and fungal therapeutic targets that can be explored mechanistically in future studies for biological significance.

## Results

### Analysis of RNA Sequencing

RNA was extracted from total lung tissue of chemotherapeutic (LD) and triamcinolone treated (SD) mice (CD1) on day two and three post *A. fumigatus (CEA10)* aerosol challenge. In both these models a 80–100% mortality is reached within two weeks, with the majority of deaths occurring between days 4–6^31^. Fungal load was determined using the quantitation of 18 s rRNA normalized to host β-actin mRNA via RT-qPCR (Supplementary Figure S1). We detected varying levels of 18 s rRNA across all normalized sample replicates; however samples with relatively low normalized levels produced substantial burden (1,752 ng of 18 s rRNA per ng of host β-actin). Total RNA was utilized for subsequent library preparation via Oligo-dT beads that capture polyA tails to generate a cDNA library of the coding transcriptome without strand information. Library sequencing via HiSeq-2500, mapping, and quality control filtration of reads resulted in approximately 16 M to 29 M paired end reads per sample replicate (Supplementary Table S1). Approximately 98% of mapped reads aligned to mouse genes, while (50,000 to 1.1 M) paired end reads mapped to *A. fumigatus* strain A1163 genes per sample replicate. The vast majority of mapped mouse reads corresponded to exonic regions (>83%), while ~10% and ~2% mapped to intronic and intergenic regions. Reads mapped to *A. fumigatus* were >75% for exonic regions, ~4% for intronic regions, and 20% for intergenic regions. Cufflinks/CummeRbund and HTSeq. 2/DeSeq. 2 based pipelines were employed to determine FPKM and count distribution, covariance between samples, fold difference in expression (log_2_ fold change > 1), and cut-off values for statistical significance (q-value < 0.05, FDR-adjusted p-value < 0.05) for mouse and fungal genes respectively (Supplementary Files S1,6, Supplementary Table S4, Supplementary Figure S2).

### Host Gene Expression

**Figure 1:**
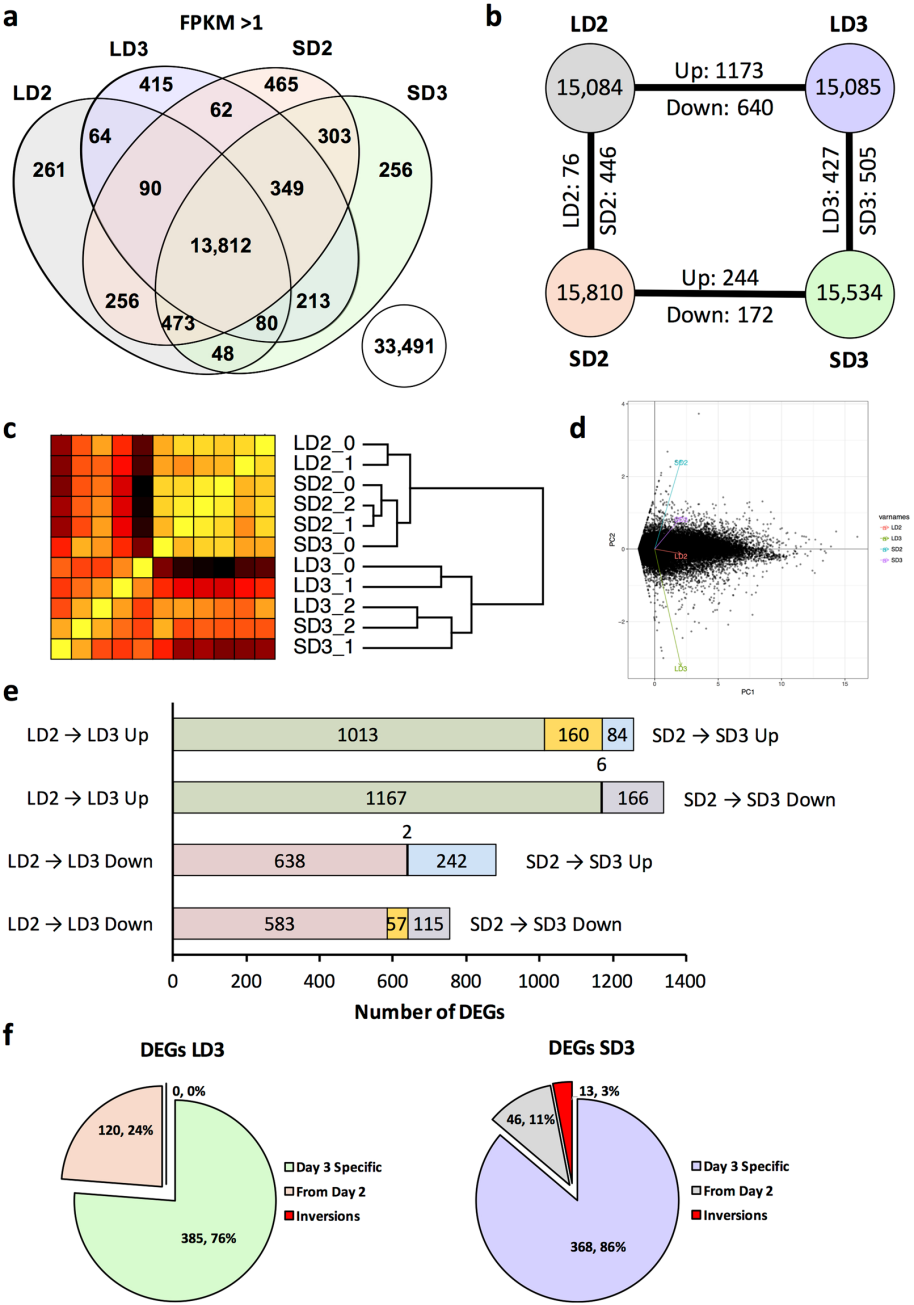
Profiles of murine transcripts during steroid and chemotherapeutic models of invasive pulmonary aspergillosis on day 2 and 3 post inoculation. Filtered, paired-end reads reads were analyzed by Cufflinks based pipeline. Transcripts with a FPKM greater than 1 were considered adequately expressed. (**a**) Venn diagram of murine transcript expression (FPKM > 1) between the two models. (**b**) Diagram of number of unique murine transcripts expressed and number of identified transcripts considered differentially expressed between the two models (FPKM > 1 in one of two comparators, log_2_-fold change > 1, and q < 0.05). (**c**) Hierarchical clustering and (**d**) principal component analysis of samples based on filtered gene expression. (**e**) Differentially expressed genes unique and conserved (yellow) between models from day 2 to day 3 post inoculation. (**f**) Composition of day 3 differentially expressed genes for a given model in respect to differential expression at day 2. Steroid Model, S; Chemotherapeutic Model, L; Day 2, D2; Day 3, D3; DEGS, differentially expressed genes.

Approximately 15,000 unique mouse genes were identified with a FPKM > 1 for either the steroid (SD) and chemotherapeutic (LD) models two and three days (D2, D3) post fungal inoculation (Fig. 1a). FPKM cutoff of 1 was chosen based on a manual investigation of the histogram of FPKM distribution across genes (Supplementary Figure S3A). 13,812 mouse genes were expressed at varying levels amongst all models and days, while 256–465 genes were uniquely expressed for a given model and day (Fig. 1a). Mouse genes, of which 33,491 had an FPKM ≤ 1, across both models and time points were not considered as adequately expressed (Fig. 1a). Hierarchical clustering analysis based upon transcript levels for each sample replicate indicated 9 of 11 replicates clustered closely with their respective counterparts (Fig. 1c). In two instances, a given sample clustered more closely with an alternate grouping, specifically a replicate of the day 3 steroid model (SD3_0 group) grouped with the replicates of the steroid model at day 2, and the chemotherapeutic model at day 3 (LD3_2) grouped more closely with the steroid model at day 3. SD3_0 and LD3_2 both had relatively low measured fungal burden in comparison to their counterparts, though both were lower in comparison to their apparent novel clustered groupings as well. Hierarchical clustering also indicated an initial separation from the root node by day and then by model. Principal component analysis and multidimensional scaling of filtered mouse genes suggests the steroid model on day 2 and the chemotherapeutic model on day 3 accounted for the highest degrees of variability while the steroid model on day 3 and the chemotherapeutic model on day 2 were significantly lower (Fig. 1d, Supplementary Figure S2). Due to the large coverage of mouse genes by each model and time point, further analysis was relegated to differentially expressed genes and clustered gene families amongst the two models and time points.

### Differentially Expressed Genes in the Host

Differential gene expression was determined between each model for a given day and between each day for a given model (Fig. 1b) using a Cufflinks based pipeline (q-value < 0.05). This output was then further filtered based on the requirement that one comparator have a FPKM > 1 and a minimum of log_2_-fold change of 1 for a given comparison (Supplementary File S1). With these requirements, 416 differentially expressed genes (244 with increased expression, 172 with decreased) were identified from day 2 to day 3 post-fungal inoculation for the steroid model, while 1,813 differentially expressed genes (1,173 with increased expression, 640 with decreased) were identified in the chemotherapeutic model from day 2 to day 3 post-inoculation (Fig. 1b). Of these 416 and 1,813 differentially expressed genes from day 2 to day 3, only 160 were increased in expression in both models, 57 were decreased in expression in both models, and 8 had inverse expression between the two models (Fig. 1e). Approximately 88% (1,596 of 1,813) of differentially expressed genes from day 2 to day 3 were unique to chemotherapeutic model, while 47% (199 of 416) of differentially expressed genes from day 2 to day 3 were unique to the steroid model (Fig. 1e). These findings suggest the majority of gene expression changes from day 2 to day 3 were exclusive to a given model. The increased number of differential expressed genes between the two models on day 3 (932 total; 427 with increased expression in chemotherapeutic, 505 with increased expression in steroid) in comparison to day 2 (522 total; 76 with increased expression in chemotherapeutic, 446 with increased expression in steroid) furthered the concept of increased divergence in gene expression (Fig. 1b).

We then looked to determine how many of the differentially expressed genes between the models on day 3 were also differentially expressed between the models on day 2. For the steroid model, approximately 76% (385 of 505) of day 3 differentially expressed genes with statistically higher expression (enriched) for the steroid models were not considered enriched on day 2. Similarly, 86% (368 of 427) of day 3 enriched genes for the chemotherapeutic model were not enriched on day 2. The identification of these various groupings of differentially expressed genes provided a template for pathway, protein class, and functional categorization and enrichment to initially describe changes between and within a given IPA model. Importantly, these gene expression data suggest that host responses to IPA disease progression is temporal and model specific.

### Enrichment and Categorization of Mouse DEGs

To characterize the differentially expressed gene groupings, we utilized a combination of statistical enrichment of both biological processes (BINGO^32,33^) and curated protein-protein interaction networks (Reactome^34^), classification of protein function (PantherDB^35^), and involvement in metabolic pathways (KEGG overlays^36^) (Tables 1 and 2, Supplementary Figure S4, Supplementary Files S2, S3). Across all methods, most statistically enriched pathways and processes were focused on aspects of immune signaling and to a lesser extent various aspects of central metabolism. The greatest number of statistically enriched pathways were associated with the large number of differentially expressed genes from day 2 to day 3 in the chemotherapeutic model (Supplementary Files S2, S3). This grouping of differentially expressed genes also had the highest degree of network connectivity amongst enriched protein-protein interaction networks via Reactome (Supplementary Figure S4). Increased connectivity suggests a concerted effort to differentially regulate multiple agents of a given biological process in the chemotherapeutic model.

**Table 1:**
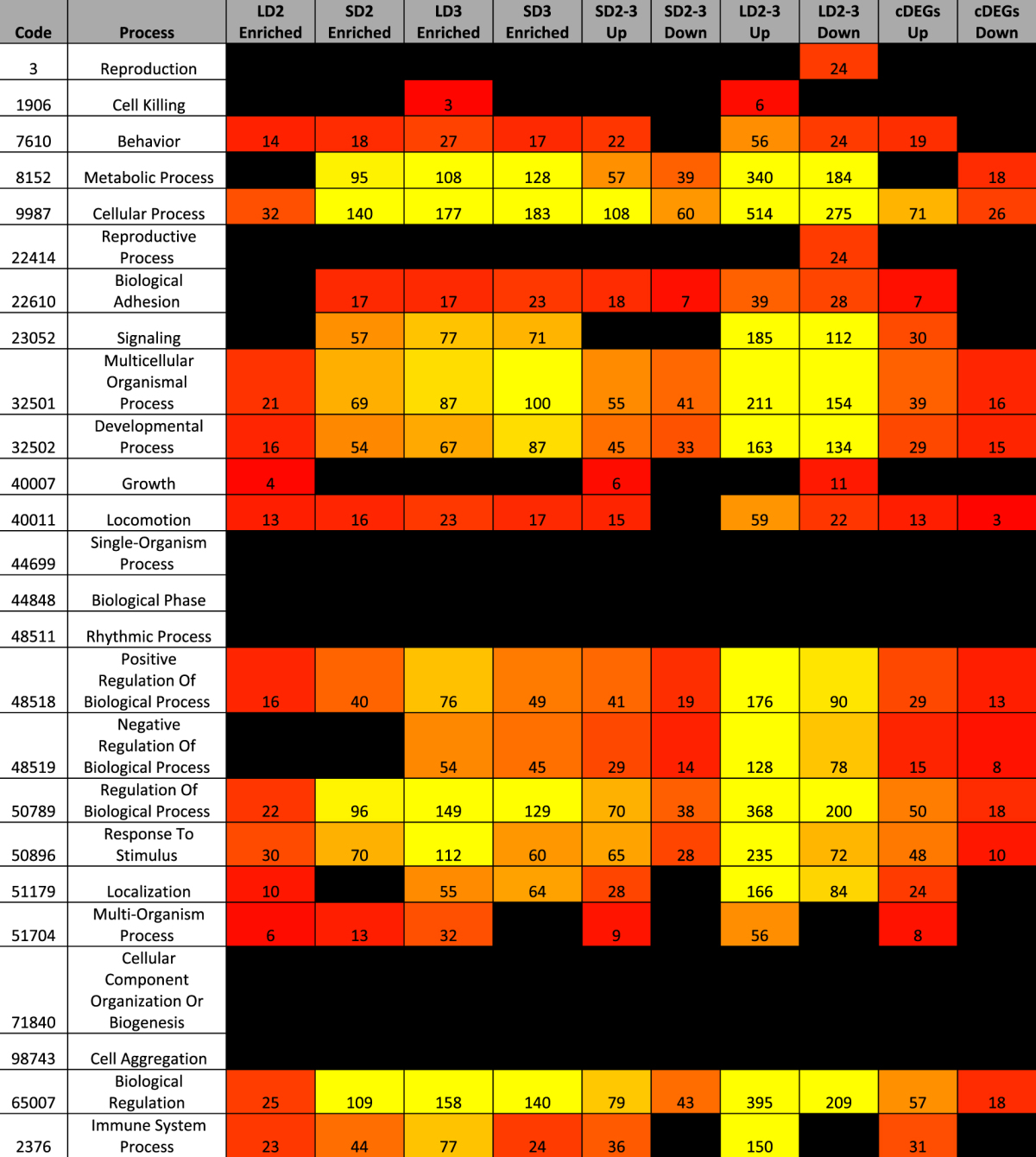
Enrichment of gene ontology biological processes specific for mice based upon differentially expressed gene groupings. Enrichment of primary (tier 1) biological functions from the gene ontology (BINGO^32,33^) based upon differentially expressed genes in the steroid and chemotherapeutic models of invasive pulmonary aspergillosis from day to day 3. LD, chemotherapeutic model; SD, steroid model; 2–3, from day 2 to 3; Up, differentially expressed genes increasing in expression; Down, differentially expressed genes decreasing in expression; Enriched, differentially expressed genes of higher transcript expression for the identified model in comparison to the alternate model for a given day; cDEGs Up, differentially expressed genes increasing in expression from day 2 to day 3 in both models; cDEGS Down, differentially expressed genes decreasing in expression from day 2 to day 3 in both models. Color code gradient from null (black) to 1 (red) to 100 (yellow).

**Table 2:**
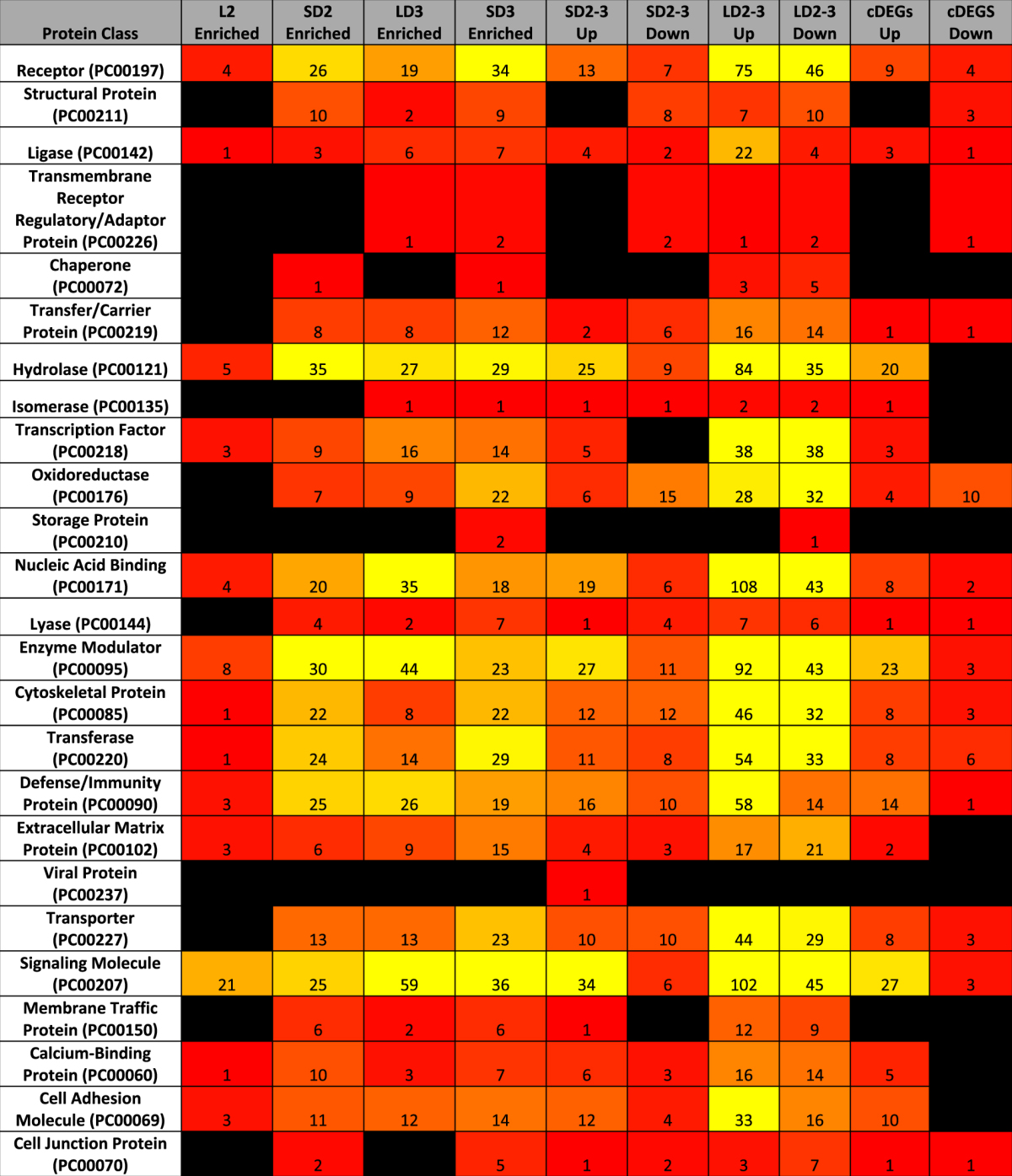
Categorization of differentially expressed genes based on PangtherDB protein class. Categorical binning of PantherDB^35^ protein classes based upon differentially expressed genes in the steroid and chemotherapeutic models of invasive pulmonary aspergillosis from day to day 3. LD, chemotherapeutic model; SD, steroid model; 2–3, from day 2 to 3; Up, differentially expressed genes increasing in expression; Down, differentially expressed genes decreasing in expression; Enriched, differentially expressed genes of higher transcript expression for the identified model in comparison to the alternate model for a given day; cDEGs Up, differentially expressed genes increasing in expression from day 2 to day 3 in both models; cDEGS Down, differentially expressed genes decreasing in expression from day 2 to day 3 in both models. Color code gradient from null (black) to 1 (red) to 30 (yellow).

Our initial assessment of statistically enriched immune pathways from Reactome (p < 0.001) in the chemotherapeutic model from day 2 to day 3 identified increased expression of “cytokines and receptors” (51 genes), and “chemokines and receptors” (39 genes) (Supplementary File S3). Of specific note was the statistical enrichment of Tnf-α (26 genes), IL-23 (14 genes), IL-12 (12 genes), and IL-2 (11 genes) signaling pathways, as well as broader Jak-STAT signaling (25 genes), NFκB signaling (16 genes), and nod-like receptor signaling (12 genes).

Differentially expressed genes in steroid model from day 2 to day 3 statistically enriched “Cytokine-cytokine receptor interaction” (15 genes), and “Chemokine signaling pathway” (11 genes). These genes also only statistically enriched TNF-α (6 genes) and IL-23 (4 genes) signaling pathways. Comparison of differentially expressed genes between the two models on day 3 revealed statistically enriched “Cytokine-cytokine receptor interaction” (34 genes) and “chemokines and receptors” (20 genes) for the chemotherapeutic model. Elevated expression of differentially expressed genes for the chemotherapeutic model in comparison to the steroid model also statistically enriched the Jak-Stat (25 genes), toll-like (18 genes), and nod-like (12 genes) innate immunity pathways.

### Analysis of Immunologically Relevant Genes

**Figure 2:**
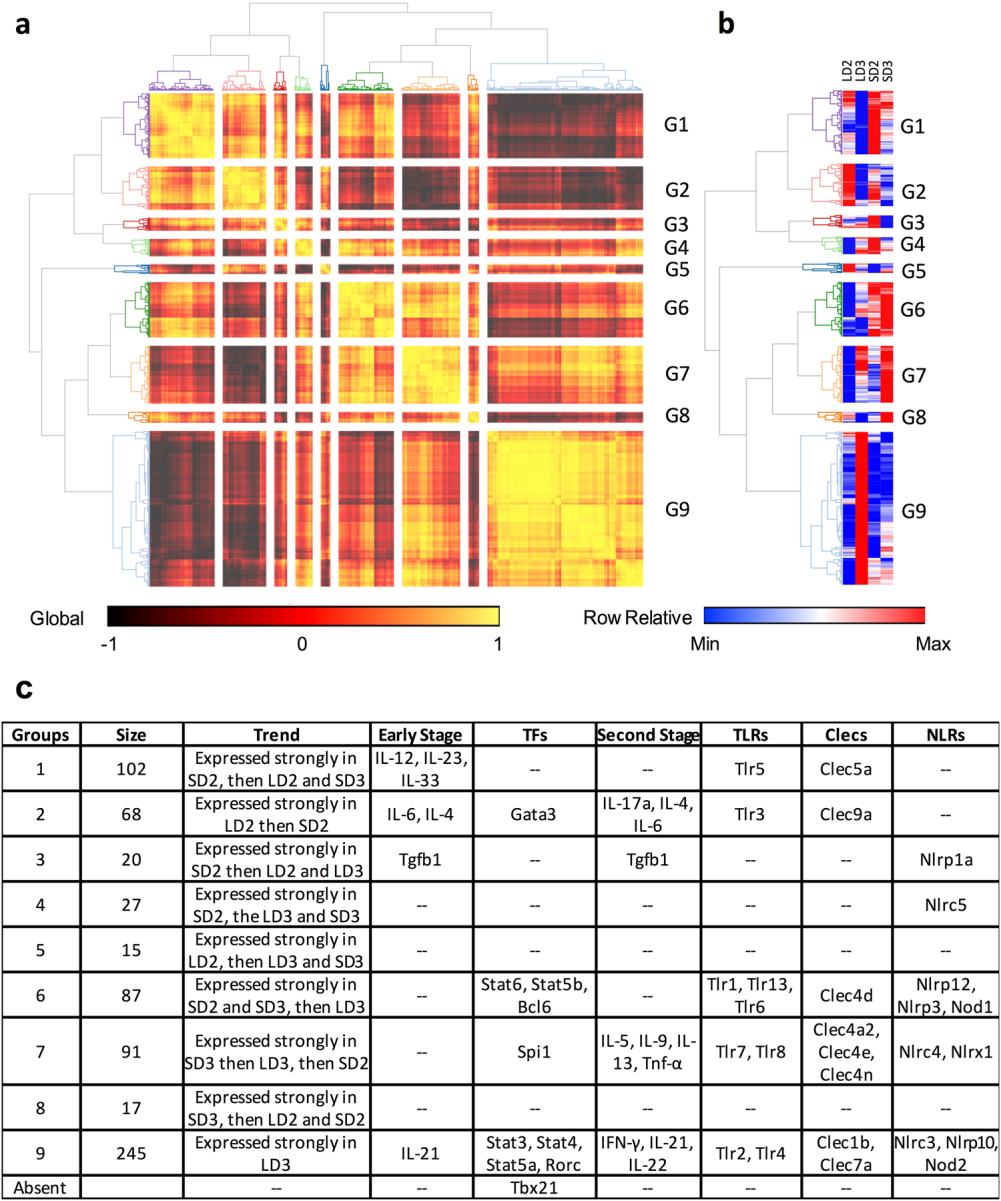
Transcript profiles of immunologically relevant murine genes. (**a**) hierarchical clustering of murine immunological relevant genes (IRGs) based on transcript expression during steroid and chemotherapeutic models of invasive pulmonary aspergillosis on day 2 and 3 post inoculation. (**b**) relative transcript expression for a given IRG. (**c**) Summary table of identified genes that cluster together based on expression. Identification of notable early stage cytokines, later stage cytokines, and transcription factors associated with T-cell differentiation, toll-like receptors (*Tlrs*), C-type lectins (*Clecs*), and Nod-like receptors (*Nlrs*) amongst clustered IRG groupings. Steroid Model, S; Chemotherapeutic Model, L; Day 2, D2; Day 3, D3.

To further understand immune signaling patterns amongst the steroid and chemotherapeutic models we clustered 672 immunologically relevant genes (IRGs) (FPKM > 1 for at least one model and time point) based on gene expression on day 2 and day 3 in the steroid and chemotherapeutic models (Fig. 2, Supplementary Table S2). Gene expression patterns fell into 9 categories with group 9 being largest in size (245 members) followed by groups 1,7,6, and 2 (68–102 members) (Fig. 2a,b). Gene expression pattern for group 9 was characterized by strong expression in the chemotherapeutic model on day 3. Other groups, such as Group 6, 2, and 7, were characterized by strong expression for more than one model and/or day.

We then independently analyzed several genes, predominately cytokines and transcription factors, known to be hallmarks and drivers of T-cell differentiation (Supplementary Figure S5). Our analysis of transcript levels from early stage cytokines of T-helper (T_h_) cell differentiation suggest a predominantly mixed T_h_1, T_h_17, T_h_2, T_h_9, and T_f_H response. For T_h_1, T_h_2, and T_h_17 associated transcription factors (TFs), only one TF (Stat4, Stat3, Stat6, Stat5) was expressed, while their counterpart transcription factors (Tbx21, Rorc, Gata3, Foxp3) remained generally repressed or lowly expressed for the chemotherapeutic and steroid models. Secondary stage cytokines suggested T_h_9 and T_f_H signaling events were not occurring in both models while T_h_17 associated cytokines were largely mitigated in the steroid model and non-existent in the chemotherapeutic. The T_h_1 associated cytokine Tnf-α was differentially increasing in the chemotherapeutic model, while consistent in the steroid model from day 2 to day 3. Our analysis demonstrates a partial activation of T_h_1, T_h_2, and T_h_17 transcription factors, followed by a low number and diminutive level of secondary stage cytokines associated with these specific T_h_ cell responses. The exception of course being the elevated expression of *Tnf-α*, which can also be viewed as an early phase or broadly pro-inflammatory cytokine. Both models resulted in an incomplete T_h_1, T_h_17, and T_h_2 signaling when factoring in expression levels of both known transcription factors and their associated secondary stage cytokines. It is important to note that leukocyte populations only make up a small portion of the total cell numbers and low expression of select cytokines and TFs could be due to lack of capturing these gene expression changes. Expression of cytokines (IL-33, Tnf-α, *etc*.) and TFs (STAT family) in relatively large cell populations, such as endothelial, epithelial, and airway smooth muscle cells would result in a greater number of reads.

**Figure 3:**
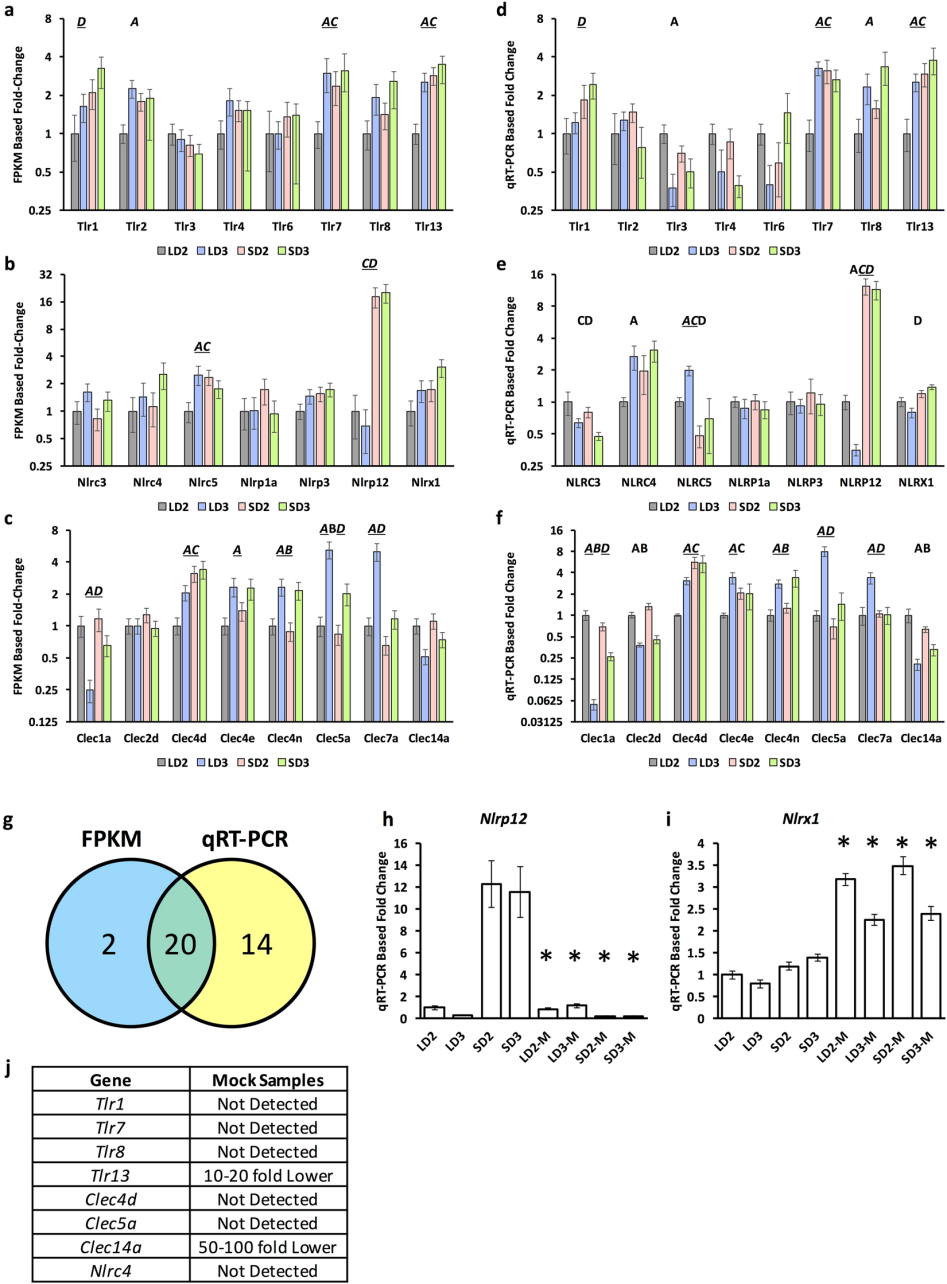
RT-qPCR analysis of murine genes differentially expressed during IPA. Fold change was determined for differentially expressed and expressed (**a,d**) Toll-like receptors, (**b,e**) Nod-like proteins, (**c,f**) and C-type lectins via FPKM analysis and RT-qPCR analysis respectively. Fold-change for RT-qPCR was normalized using *β-tubulin*, and related to LD2 gene expression (comparator) using the *ΔΔCT* method. For FPKM based analysis, data is presented in relation to LD2 gene expression (comparator). Statistical significance is indicated for instances where p < 0.05. A/B statistically significant differential expression between day 2 and day 3 for the chemotherapeutic/steroid model respectively. C/D, statistically significant differential expression between the chemotherapeutic and steroid model for day 2/day 3 respectively. Underlined letters indicate statistical significance by both FPKM and RT-qPCR. (**g**) Summary of total instances of differential gene expression between FPKM and RT-qPCR based methods. Gene expression of (h) *Nlrp12* and (**i**) *Nlrx1* in inoculated and mock (−M) inoculated samples. Statistical significance (*) is indicated where p < 0.05 for comparisons between a mock and inoculated sample. (**j**) Summary of analyzed genes from mock samples not expressed or expressed at a significantly lower level (>10-fold decrease in expression in comparison to inoculated sample). Steroid Model, S; Chemotherapeutic Model, L; Day 2, D2; Day 3, D3.

We subsequently identified these transcription factors and cytokines amongst our IRG groupings (Fig. 2c) as well as *Tlrs*, *Clecs*, and *Nlrs* to gain insight into trends in gene expression of IRGs in relation to cytokine and transcription factor expression. Many of the expressed *Clecs* and *Nlrs* have no previous association with invasive pulmonary aspergillosis, while others such as Dectin-1 (Clec7a), Dectin-2 (Clec4n/Clec6a), and MINCLE (Clec4e) are well studied or associated with IPA or fungal infection^37–41^. Using the same total RNA we used for RNA-Seq, we then analyzed the expression of 23 of these genes using RT-qPCR (Fig. 3). Of the 31 instances of differential expression identified by our Cufflinks pipeline, only 22 met the additional criteria of being greater than 1-fold in difference. Of these 22 instances of differential expression, 20 were verified to be differentially expressed via RT-qPCR, while the remaining 2 were not considered differentially expressed. An additional 14 were further identified as differentially expressed via RT-qPCR. For 9 of these 14 instances, the relative transcript levels determined by RT-qPCR were in general agreement with the relative FPKM transcript level, but due to the sensitivity of RT-qPCR were now considered statistically significant. It will be interesting to compare the remaining 5 instances with FPKM based isoform abundances, as RT-qPCR maybe biased towards specific isoforms.

We then analyzed 10 genes for expression in control samples, where mice were respectively immunosuppressed and then mock inoculated with sterile PBS. RNA was extracted on day 2 and day 3 post mock inoculation and analyzed via RT-qPCR. *Tlr1*, *Tlr7*, *Tlr8*, and *Tlr13* as well as *Clec4d*, *Clec5a*, *Clec15a*, and *Nlrc4* were all found to be differentially expressed or expressed amongst the *A. fumigatus* inoculated samples, but could not be detected or were >10-fold lower in expression in the mock inoculated samples. *Nlrp12*, which was highly expressed in the in the *A. fumigatus* inoculated steroid model, was lowly expressed in the mock inoculated samples for both models (Fig. 3h). This puts forth the notion that *Nlrp12* expression is context specific to both *A. fumigatus* and steroid treatment. *Nlrx1*, which we had found to be expressed in both models, was statistically elevated in mock samples for both models and days (Fig. 3i). The contrasting expression from day 2 to day 3 for *Nlrx1* between inoculated and mock inoculated samples suggests it may be impacted by a number of concurrent events that shape its role as an attenuator of inflammation.

### Simulating CD4+ T-cell Differentiation

**Figure 4:**
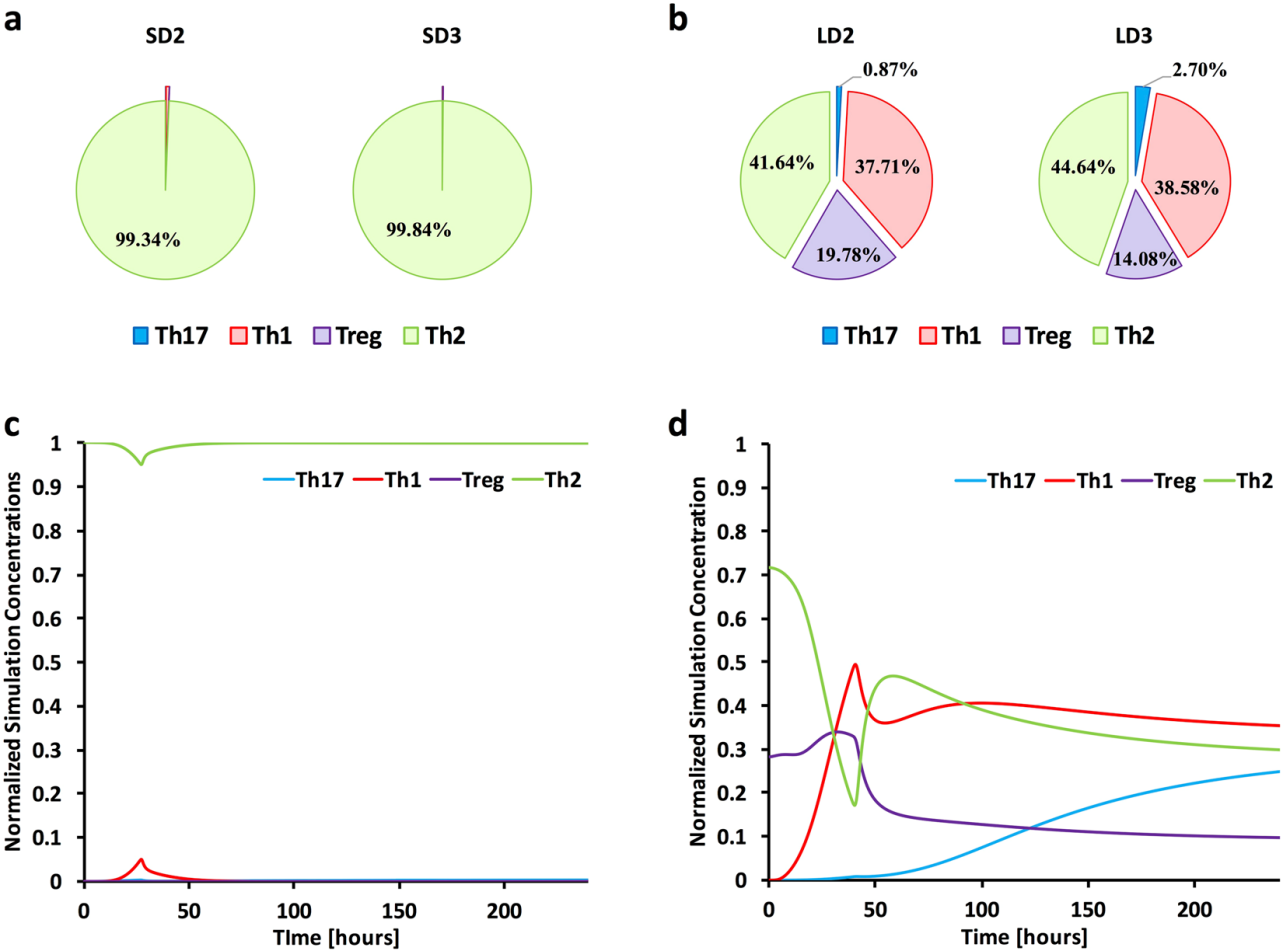
Predictive systems modeling of CD4+ T cell differentiation. Cytokine (IL-18, IL-12, IFN-γ, IL-21, IL-6, IL-17, IL-23, IL-4, TGF-β, IL-2, IL-10) and receptor (IL-18r, IL-12r, IFN-γr, Il-6r, IL-17r, IL-23r, IL-4r, TGF-βr, IL-2r, IL-10r), and transcription factor (Tbx21, Gata3, Foxp3, Rorc) expression data was compiled and used as input for computational simulations. In silico models were separately calibrated using the particle swarm method. (**a,b**) The overall predicted CD4+ subset composition for day 2 and day 3 for both the steroid and chemotherapeutic models. (**c,d**) Model specific time course predictions from 0 to 240 hours post inoculation for the steroid and chemotherapeutic model. Steroid Model, S; Chemotherapeutic Model, L; Day 2, D2; Day 3, D3.

Our qualitative analysis of expressed stage 1 and stage 2 cytokines and transcription factors associated with T_h_-cell differentiation suggested strongly tempered and partial T_h_ mediated responses that were due to either biological phenomena or inability to capture reads from this relatively low population of cells. We utilized an established predictive model of CD4 T_h_ differentiation built on ordinary differential equations to understand the effect of specific immune molecules on initial and cross-talk signaling in the chemotherapeutic model and steroid model of IPA. This computational modeling approach has been utilized successfully to predict differentiation and plasticity of CD4+ T cells in the gastrointestinal tract of mice in response to pharmacological activation of PPARγ that were then validated *in vivo* ^42–45^. The computational models were based on the overall transcript levels of a number of cytokines (IL-18, IL-12, IFN-γ, IL-21, IL-6, IL-17, IL-23, IL-4, TGF-β, IL-2, IL-10), receptors (IL-18r, IL-12r, IFN-γr, Il-6r, IL-17r, IL-23r, IL-4r, TGF-βr, IL-2r, IL-10r), and a subset of transcription factors (*Tb21*, *Gata3*, *Foxp3*, *Rorc*) from our RNA-Seq study to determine the likelihood for each CD4+ subset and overall CD4+ subset composition throughout the course of infection (Fig. 4, Supplementary Figure S6). *In silico* modeling resulted in early and overall T_h_2 response for the steroid model with marginal instances of T_h_1, T_h_7, and T_reg_ cell populations (Fig. 4a–c). The modeling also predicted an early, but ablating T_h_2 response, a low yet rapidly increasing T_h_1 response, a weak though slowly increasing T_h_17 response, and a steady initial T_reg_ response that then decreased for the chemotherapeutic model.

**Figure 5:**
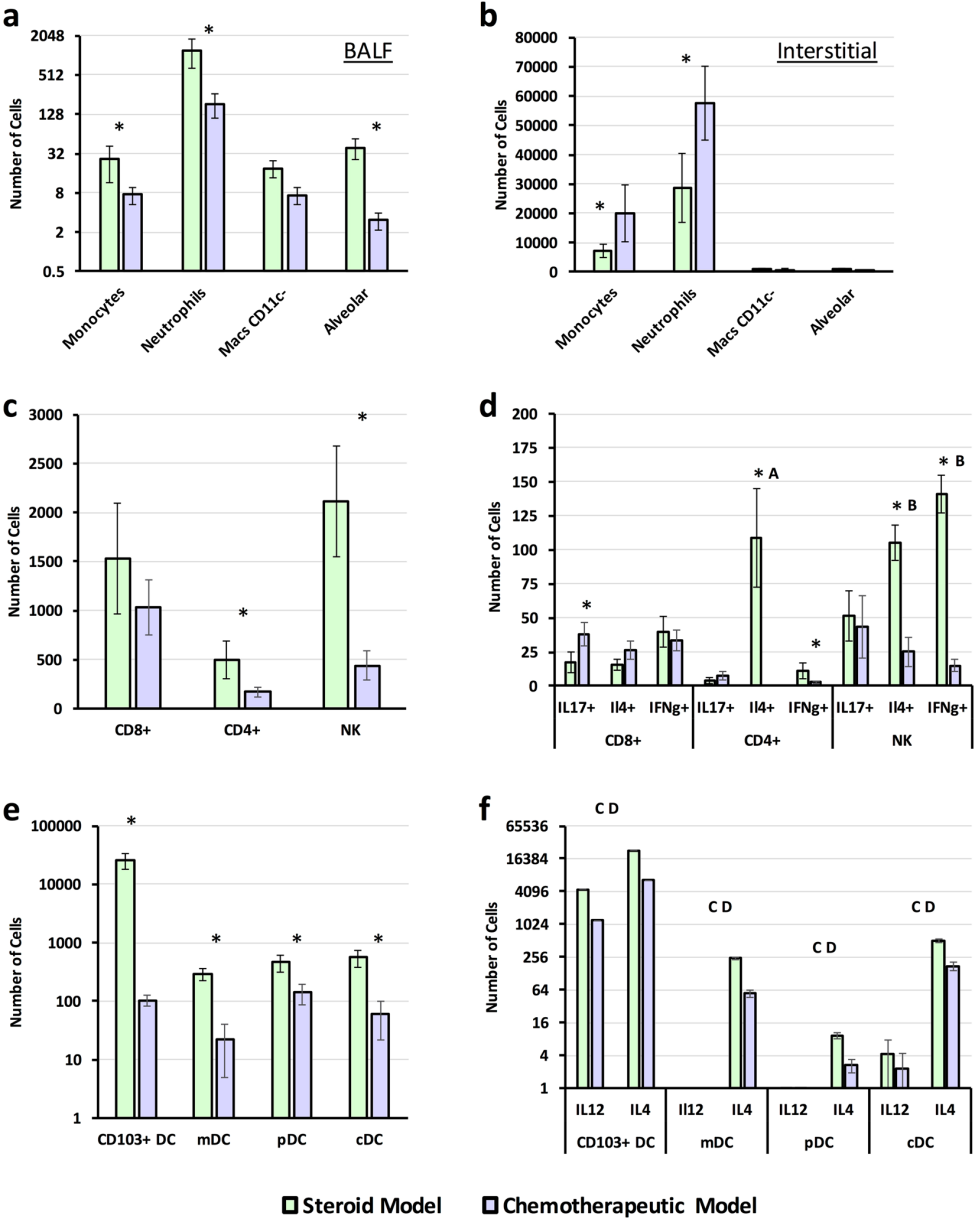
Determination of *in vivo* leukocyte and intracellular cytokine production. Freshly harvested *A. fumigatus conidia* (CEA10, 12 × 10^9^) were delivered via aerosolization to immunosuppressed C57BL/6 mice. Mice in the steroid model were immunosuppressed via subcutaneous injection of cortisone acetate, while mice in the chemotherapeutic model were immunosuppressed via subcutaneous injection of cortisone acetate and intraperitoneal injection of cyclophosphamide three days prior to inoculation. Leukocyte populations and counts were determined from (**a**) bronchoalveolar lavage fluid (BALF) and (**b**–**f**) lung tissue. Cell counts were determined for (**c**) CD4+, CD8+, and NK cell populations and (**d**) for positive intracellular staining of IL-17, IL-4, or IFN-γ. Cells counts were also determined (**e**) CD103+, monocytoid (mDC), plasmocytoid (pDC), and conventional (cDC) dendritic cells and for (**f**) positive intracellular staining of IL-12 and IL-4. Statistical significance was determined use the student t-test. Statistical significance (p < 0.05) between the two models for a given cell type is indicated by an asterisk. Statistical significance via Duncan’s multiple range test (p < 0.05) for CD4+ T cells stained positive IL-4, IL-17, and IFN-γ is indicated with ‘A’ (steroid model). Statistical significance via Duncan’s multiple range test (p < 0.05) for NK cells stained positive IL-4, IL-17, and IFN-γ is indicated with ‘B’ (steroid model). Statistical significance (p < 0.05) between a given dendritic cell population for count of cells positive for IL-12 versus IL-4 production is indicated with an ‘C’ in the steroid model, and a ‘D’ for the chemotherapeutic model. All experiments were run in independent replicates, n = 8.

To determine if our *in silico* analysis provided accurate insight into T_h_ cell response, we analyzed BALF and interstitial leukocyte populations for IL-4, IL-12, IFN-γ, and/or IL-17 production on day 3 post inoculation (Fig. 5). We observed statistically significant (p < 0.05) decrease in interstitial CD4+, CD8+, NK, and dendritic cell populations for the chemotherapeutic model in comparison to the steroid model with the exception of interstitial monocytes and neutrophils, which were significantly higher in the chemotherapeutic model (p < 0.05) (Fig. 5). In addition, we also noted a characteristic increase in neutrophil counts in BALF samples for the steroid model in comparison to the chemotherapeutic model (p < 0.05) (Fig. 5a). Analysis of CD4+ cells in the steroid model indicated a significantly large number of cells were positive for IL-4 production in comparison to a small number of cells were positive for IFN-γ or IL-17 production (p < 0.05) (Fig. 5d). A large number of NK cells were also positive for IL-4 and IFN-γ production in the steroid model in comparison to IL-17 (p < 0.05), while the few CD8+ cells positive for cytokine production indicated an evenly mixed T_h_ response.

Analysis of CD4+ cells in the chemotherapeutic model indicated a near 5-fold statistically significant reduction in number of cells in comparison to the steroid model (p < 0.05) (Fig. 5c). Very few CD4+ T cells (< 20) were positive for IL-4, IFN-γ or IL-17 (Fig. 5d). NK cells from the chemotherapeutic model were significantly depleted in comparison the steroid model (p < 0.05). The few NK cells from the chemotherapeutic model that were positive for a given cytokine were skewed towards IL-17, then IL-4 and then IFN-γ production. CD8+ T cells from the chemotherapeutic model were also depleted in relation to the steroid model and the few cells positive for cytokine production suggest a mixed response.

We then analyzed IL-4 and IL-12 production by specific dendritic cells populations (monocytoid, plasmacytoid, conventional, and CD103+) to determine how these APCs were contribution to the overall immune response. Analysis of cell populations indicated CD103+ dendritic cells were significantly depleted, over 100-fold, in the chemotherapeutic model in comparison to the steroid (p < 0.05) (Fig. 5e). Further analysis of this subset and other dendritic cell subsets clearly indicated a 5–10x fold statistical increase in number of dendritic cells positive for IL-4 in comparison to IL-12 for both models (p < 0.05) (Fig. 5f). The overall response and larger number of IL-4+ dendritic cells in the steroid model would favor a predominately strongly T_h_2 response observed by T_h_ cells as predicted by our *in silico* modeling. Due to the depleting nature of cyclophosphamide we were unable to confidently validate if our *in silico* populations were indeed representative of the CD4+ response. However, we did observe preferential elevated IL-4 production by DC population in comparison to IL-12. This finding further insinuates a skewed T_h_2 response that we observed in the *in silico* chemotherapeutic model for CD4+ T cell differentiation.

### Analysis of Metabolic Genes

Based on the large number of differentially expressed genes associated with metabolic processes (Table 1), we overlaid these differentially expressed gene groupings onto the KEGG mouse metabolic pathways^36^ (Supplementary Figure S7). Analysis of differentially expressed genes from day 2 to day 3 in the chemotherapeutic model identified functionally related enzymes, L-amino-acid oxidase, arginase, argininosuccinate synthetase 1, and nitric oxide synthase, that provide a pathway for L-aspartate mediated arginine biosynthesis and metabolism as a precursor for nitric oxide or urea generation via the urea cycle (Supplementary Figure S7A). These specific genes were also differentially expressed in the steroid model from day 2 to day 3 (Supplementary Figure S7C); however, further analysis indicates these genes are significantly more highly expressed in the chemotherapeutic model (Supplementary Figure S7F). Differentially expressed genes from day 2 to day 3 in the chemotherapeutic models also enriched oxidative phosphorylation pathways associated with the TCA cycle, nucleotide metabolism, lipid metabolism, and complex sugar metabolism (Supplementary Figure S7A), suggesting important immunometabolic interactions. Analysis of statistically enriched genes in the steroid model on day 3 identified genes associated with fatty acid oxidation, connections from glycolysis and the TCA cycle to fatty acid oxidation, and aspects of sugar and starch metabolism (Supplementary Figure S7H).

**Figure 6:**
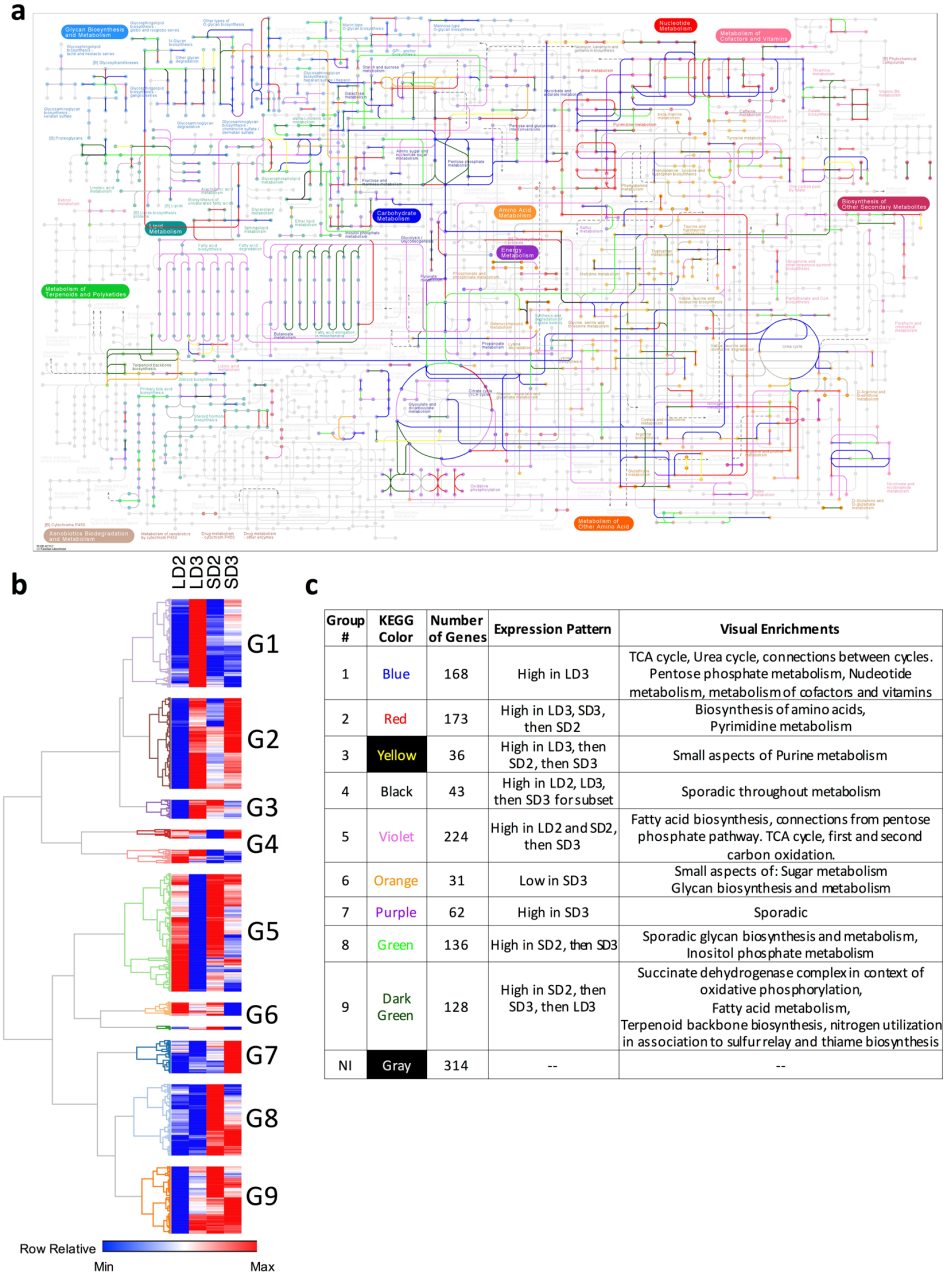
Overlay of gene clusters onto the KEGG primary metabolism for mice. Clusters were generated from gene expression during steroid and chemotherapeutic models of invasive pulmonary aspergillosis on day 2 and 3 post inoculation. Filtered, paired-end reads with a FPKM > 1 were (**b**) hierarchically clustered based on transcript expression. (**a**) Resultant groupings were color coded and overlaid onto the KEGG primary metabolism for mice^36^. (**c**) Summary table of identified groups: overlay color, number of genes in cluster, expression pattern, visual enrichments. Steroid Model, S; Chemotherapeutic Model, L; Day 2, D2; Day 3, D3.

To advance our understanding of metabolic changes during the steroid and chemotherapeutic models, we identified 1,317 genes from the KEGG primary metabolic pathway to cluster based on gene expression (Supplementary Table S3). We initially filtered these genes based on minimum gene expression (FPKM > 1) for at least one of the four model/timepoints, and then clustered the resultant 1,001 genes based on expression patterns from the steroid and chemotherapeutic models of IPA. The resulting hierarchical clustering resulted in 9 groups varying in size from 31 to 244 members (Fig. 6b). These groupings were then overlaid onto the KEGG metabolic pathway and visually assessed for association with broad and specific processes (Fig. 6a–c, Supplementary File S4). These groupings were also used for specific statistical enrichment (p < 0.001) of biological processes associated with the gene ontology (BINGO) and Reactome (Supplementary File S5). Group 1 consisted of genes highly expressed in the chemotherapeutic model on day 3 and statistically enriched a substantial portion of TCA cycle, urea cycle, pentose phosphate metabolism, nucleotide metabolism and the metabolism of cofactors and vitamins. Several group 1 genes also linked the urea cycle to the TCA cycle. Groups 7, 8 and 9 represented genes generally highly expressed in the steroid model over the chemotherapeutic model. These genes were associated with inositol phosphate metabolism, fatty acid oxidation, terpenoid biosynthesis, thiamine biosynthesis, sulfur relay, and oxidative phosphorylation. Group 2 generally represented genes being turned on in both the chemotherapeutic and steroid model from day 2 to day 3. These genes were associated with the biosynthesis of amino acids and pyrimidine metabolism. Group 5 represented gene expression decreases in both models from day 2 to day 3, with an emphasis on larger decrease in the chemotherapeutic model. These genes were associated with fatty acid biosynthesis, connections from pentose phosphate pathway to fatty acid biosynthesis, and specific aspects of first and second carbon oxidation in the TCA cycle.

We then specifically looked at changes in gene expression between the two models in glycolysis, the citric acid (TCA) cycle, and the arginine biosynthesis (urea cycle) pathway (Supplementary Figure S8, Supplementary Table S3). Analysis of genes involved in glycolysis in the chemotherapeutic model from day 2 to day 3 suggested increased expression of lactate dehydrogenase (Ldha, from ~211 to ~465 FPKM, respectively) converting pyruvate to lactate, and minimal changes in gene expression of pyruvate dehydrogenase (Pdha1, 67–70 FPKM), the first component enzyme in converting pyruvate to acetyl-CoA (Supplementary Figure S8A–C, Supplementary Table S3). Genes associated with the conversion of ethanol to acetyl-CoA (Adh1, Aldh3a1, Aldh7a1, and Acss2) were also significantly down regulated (>0.5-fold) in the chemotherapeutic model. Additionally, we observed an increase in expression (>0.3-fold) from day 2 to day 3 in genes (Hk1, Hk2, Hk3, Adpgk, and Gpi1) associated with the isomerization and/or phosphorylation of α/β-glucose to α-D-glucose-6-phosphate and β-D-fructose-6-phosphate. Similar expression patterns were apparent in the steroid model from day 2 to day 3 as these same genes were respectively increased or decreased in expression.

Direct comparison of gene expression on day 3 between the two immunologically distinct models suggest stronger gene expression of specific sugar kinases for each model, while both models were consistent for very elevated Ldha (465–480 FPKM) and moderate Pdha1 expression (~72 FPKM). Specific increased expression of Phosphoenolpyruvate carboxykinase (Pck1, 4-fold) was also noted in the steroid model suggesting activation of gluconeogenesis or replenishment of phosphoenolpyruvate in this model. Analysis of fructose bisphosphate (Fbp2), another important driver of gluconeogenesis, indicated moderate expression (~40 FPKM), while glucose-6-phosphatases (G6pc, G6pc2) were not considered expressed (FPKM < 1).

Analysis of the TCA cycle from day 2 to day 3 in the chemotherapeutic model suggested a diminished expression in a part of the pathway from the conversion of isocitrate to succinyl-CoA encompassing isocitrate dehydrogenase (Idh1, Idh3g) and oxoglutarate dehydrogenase (Ogdh). This decrease in gene expression was also observed for fumarate hydratase 1, which is responsible for the conversion of fumarate to malate (Supplementary Figure S8D–F). These specific changes result in a “broken TCA cycle,” which is defined as build up of TCA intermediaries for alternate biological functions^46^. The precursor substrates at the site of these breaks, are known intermediaries to amino acid biosynthesis pathways particularly arginine biosynthesis, alanine, aspartate, tyrosine and glutamate metabolism. Gene expression analysis of the TCA cycle during the steroid model from day 2 to day 3 reveals minor increased expression for various enzymes. Analysis of gene expression between the chemotherapeutic and steroid model on day 3 reiterated the broken TCA cycle in the chemotherapeutic model, but not the steroid model.

Examination of the gene expression changes in the arginine biosynthesis pathway from day 2 to day 3 in the steroid and chemotherapeutic model suggested increased expression of nearly all intermediaries from the TCA cycle to the urea cycle as well as 4 of 5 enzymes in the urea cycle (Supplementary Figure S8G–I). Of importance is the increased expression of both nitric oxide synthase (Nos2 and Nos3) and arginase (Arg1) that are involved in the production of nitric oxide and urea respectively. In both models, the amount of Arg1 expression was significantly greater than that of Nos2 on day 3 (3.5-fold for the chemotherapeutic model and 5.3-fold for the steroid model) (Supplementary Table S3). We then looked at known cationic amino acid transporter genes to determine if it was possible for arginine to reach these respective enzymes^47^ (Supplementary Figure S8J). CAT-1 and CAT-2 were increasingly differentially expressed in both models and had moderate FPKMs (5–22 FPKM), while CAT-3 and CAT-4 were fairly low in expression for both models and time points (FPKM <  = 1.3). The light chain subunit of the cationic amino acid transporter y+Lat1 and y+Lat2 were also moderately expressed while the heavy chain subunit 4F2HC was highly expressed. Our analysis implies host arginine transport is likely not constrained in either model during IPA.

### Conserved and Unique Fungal Genes Across Models

**Figure 7:**
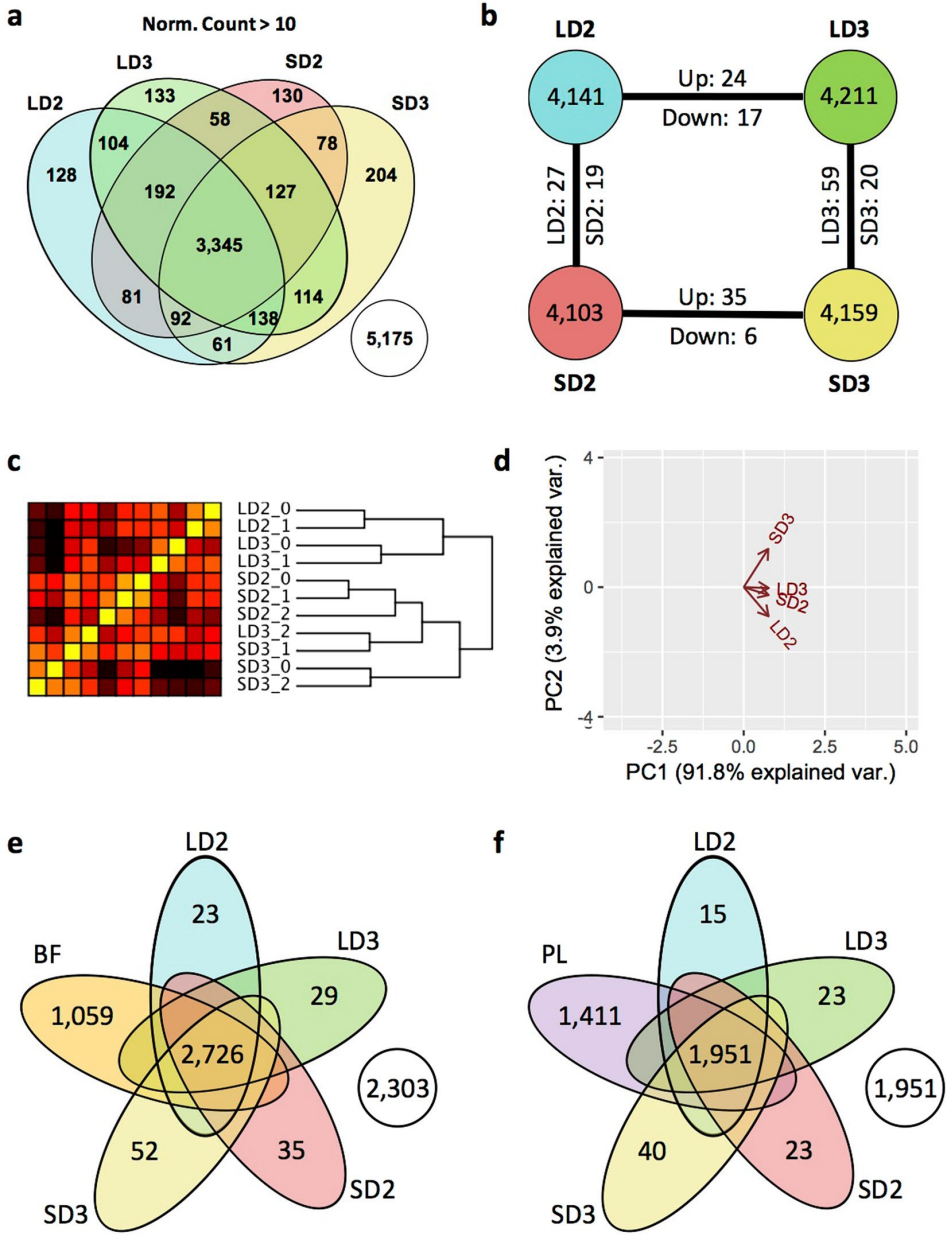
*A. fumigatus* transcript profiles during steroid and chemotherapeutic models of Invasive pulmonary aspergillosis day 2 and 3 post inoculation. Filtered, paired-end reads murine associated reads were analyzed by HTSeq. 2/DeSeq. 2 pipeline. Transcripts with a normalized count greater than 10 were considered adequately expressed. (**a**) Venn diagram of *A. fumigatus* transcript expression between the two models. (**b**) Diagram of number of unique *A. fumigatus* transcripts expressed and number of identified transcripts considered differentially expressed between the two models. (**c**) Hierarchical clustering and (**d**) principal component analysis of samples based on filtered gene expression. Venn diagram of fungal gene expression incorporating gene expression data sets of (**e**) biofilm (BF) and (**f**) planktonic (PL) growth (RPKM > 10). Steroid Model, S; Chemotherapeutic Model, L; Day 2, D2; Day 3, D3.

Fungal gene expression was initially analyzed via the Cufflinks pipeline and resulted in skewing of gene expression due to a higher abundance of fungal reads in the chemotherapeutic samples. This overall skewing of reads was in accord with our measurement of fungal load as samples with lower RNA-Seq counts also had lower relative fungal burden and vice versa (Supplementary Figure S1, Supplementary Table S1). Supplementary Figure S9 additionally highlights this finding in regards to RT-qPCR validation of 9 putative secreted fungal proteins, where we observed the fold normalization of FPKM gene expression was skewed towards lower values in the steroid model. Based on this apparent discord between the fold normalized FPKM and RT-qPCR fold changes, we then utilized the HTSeq. 2/DeSeq. 2 pipeline to determine normalized counts and differential expression for fungal genes (Fig. 7, Supplementary Table S4). Histogram distribution of normalized counts resulted in a selected cut-off for basal expression at 10 (Supplementary Figure S3B). 3,345 unique *A. fumigatus* genes were identified with a normalized count > 10 for the steroid and chemotherapeutic models on day two and day three (Fig. 7a), while 128–204 genes were uniquely expressed for a given model and day. 5,175 *A. fumigatus* genes were not expressed with a normalized count > 10. Hierarchical clustering analysis of each sample for a given model and day indicates samples were highly similar with a minimum correlation of 0.811 between samples (range −1 to 1). Given this close correlation, individual samples for a given model and day did not always cluster directly with their respective replicates. This was the case for both SD3_1 and LD3_2 (Fig. 7c). Hierarchical clustering analysis indicated an overall initial separation by model and then by date. PCA analysis resulted in similar clustering and suggested the vast majority of variance (91.8%) was explained by only 1 principal component. The greatest variance was found between chemotherapeutic model on day 2 and steroid model on day 3, while the chemotherapeutic model on day 3 and steroid model on day 2 had lower variance (Fig. 7d). Integration of gene expression (RPKM > 10) of vegetative growth from *Gibbons et al*.^23^ indicates a higher number of expressed genes shared with biofilm growth in comparison to planktonic growth (Fig. 7e,f).

### Enrichment and Categorization of A. fumigatus DEGs

*A. fumigatus* differentially expressed gene groupings (Fig. 7b, Supplementary File S6) were analyzed by FunCat, and GO for statistical enrichment and categorization of biological processes, pathways, and protein classification (p_adj_ < 0.05) (Supplementary Files S7, S8). Genes enriched in the steroid model on day 2 were associated with translation, cation (transmembrane) transport, and glutamate catabolic metabolism to 2-oxoglutarate. Genes enriched in the chemotherapeutic model on day 2 were associated with oxidation-reduction and fatty acid biosynthesis, tetracyclic and pentacyclic triterpenes (cholesterin, steroids and hopanoids) metabolism, and triterpenes metabolism. Genes enriched in the steroid model on day 3 include those annotated to be involved in oxidation-reduction, secondary metabolism, siderophore-iron transport, metabolism of thioredoxin, glutaredoxin, glutathione, heavy metal ion transport (Cu+, Fe3+, etc.), homeostasis of metal ions (Na, K, Ca etc.), and non-ribosomal peptide synthesis. As the importance of gliotoxin production was previously reported to be relevant to the steroid model of IPA and not the chemotherapeutic model (for murine survival), we analyzed the normalized counts of genes associated with gliotoxin biosynthetic pathway (Supplementary Figure S10). In several cases, specifically GliI, GliJ, GliC, GliN, GliF, the transcript levels were diminished in the chemotherapeutic model while increased in the steroid model from day 2 to day 3. Differential regulation of the gliotoxin biosynthesis cluster reiterates the notion of host context specific gene expression by *A. fumigatus*.

### Analysis of A. fumigatus Secreted Proteins by Gene Expression

**Figure 8:**
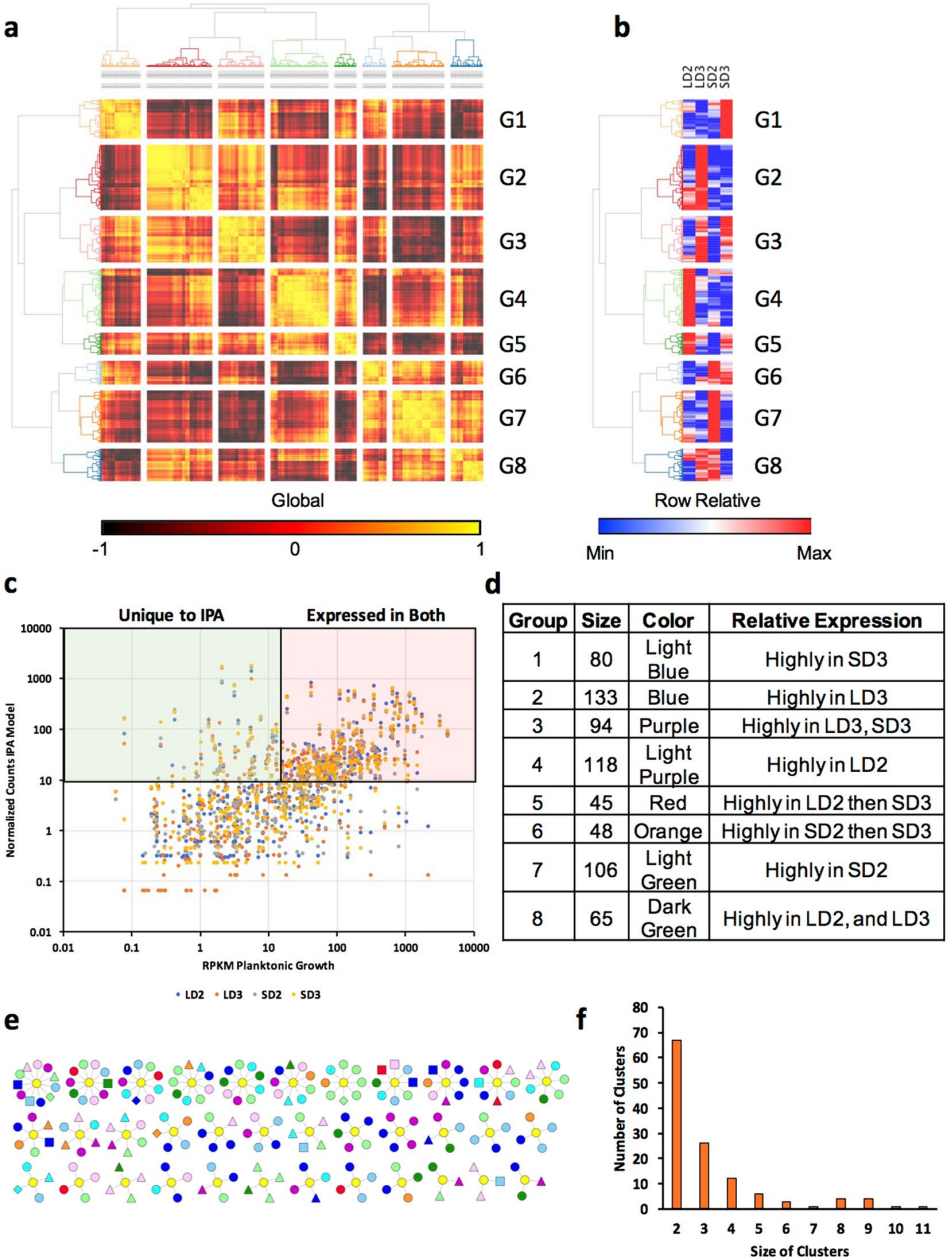
Clustering and expression analysis of the putative secretome from *Aspergillus fumigatus*. (**a**) Hierarchical clustering of the putative secretome of *A. fumigatus* Af1163 by gene expression during the steroid and chemotherapeutic models of invasive pulmonary aspergillosis on day 2 and day 3 post inoculation. (**b**) Hierarchical clustering based upon gene expression during the steroid and chemotherapeutic models on day 2 and day 3 post inoculation for the putative secretome of *A. fumigatus* Af1163. (**c**) Scatter plot of putative secreted protein gene expression (normalized counts) at LD2 (Blue), LD3 (Orange), SD2 (Gray), and SD3 (Yellow) versus expression during planktonic growth (RPKM). (**d**) Summary table of gene expression groupings: size of groupings, color code for clustering, and relative expression. (**e**) Clustering of putative secreted proteins based on sequence similarity. Color of nodes indicate grouping from gene expression (**a,b**) *see* (**d,e**). (**f**) Distribution of clusters based on size. Steroid Model, S; Chemotherapeutic Model, L; Day 2, D2; Day 3, D3.

**Figure 9:**
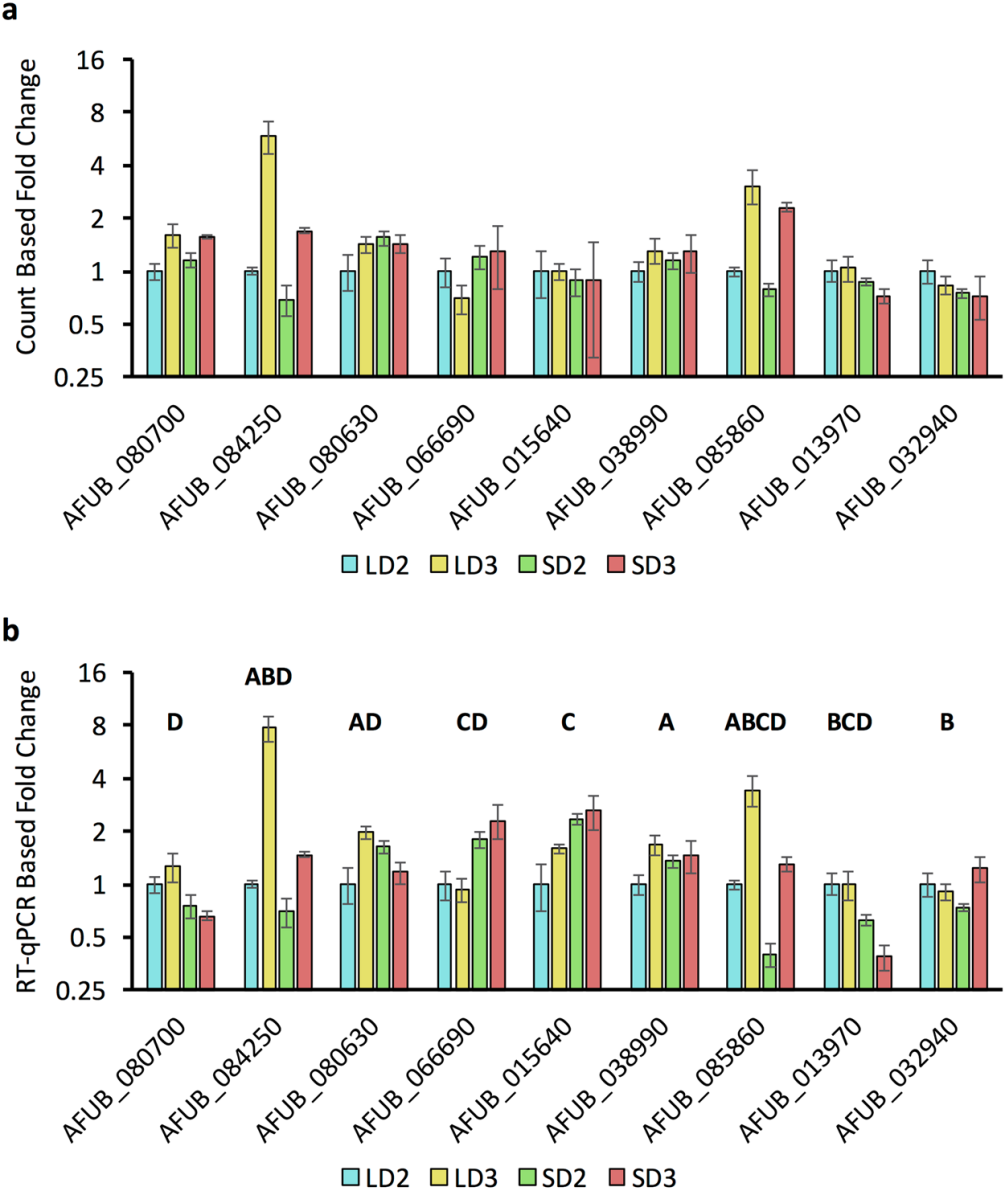
RT-qPCR analysis of *A. fumigatus* genes expressed during chemotherapeutic and steroid treatment mouse models of invasive pulmonary aspergillosis on day 2 and 3 post inoculation. Fold change was determined for expressed putative *A. fumigatus* secreted proteins via (**a**) Count based analysis and (**b**) RT-qPCR analysis. Fold-change was normalized for RT-qPCR using *β-tubulin* and *TefA*, and presented in relation to LD2 gene expression (comparator) using the *ΔΔCT* method. For count based analysis data is presented in relation to LD2 gene expression (comparator). Statistical significance is indicated for instances where p < 0.05. A/B statistically significant differential expression between day 2 and day 3 for the chemotherapeutic/steroid model respectively. C/D, statistically significant differentially expression between the chemotherapeutic and steroid model for day 2/day 3 respectively. Standard deviation from the mean is presented. Steroid Model, S; Chemotherapeutic Model, L; Day 2, D2; Day 3, D3.

We identified a predicted secretome of *A. fumigatus* through a signalP based pipeline. This secretome was then concurrently clustered via gene expression for a given model and time and fell into 8 broad categories (Fig. 8a,b). RPKM values for planktonic growth from the *Gibbons et al*. study^23^ were then integrated with normalized count data for a given *A. fumigatus* gene predicted to encode a secreted protein. We identified 199 highly expressed genes in both PL and IPA conditions and 59 genes that were highly expressed only during IPA (Fig. 8c). We then clustered our predicted secreted proteins from *A. fumigatus* based on similarity in primary amino acid sequence using the mature peptide sequence (Supplementary File S9). The 760 proteins clustered into predominantly 2–4 member tribes (105 tribes), while 20 tribes were greater than 4 members in size (Fig. 8f). Integration of gene expression into a visualization of clusters demonstrated diversity in gene expression grouping amongst all clusters (Fig. 8e). Larger tribes contained sporadic group members who were expressed uniquely during IPA (diamond) or expressed highly in both PL growth and IPA (triangle) (Fig. 8e, Supplementary Table S5, Supplementary File S10). A subset of these putative secreted proteins (9 in total) were analyzed by RT-qPCR (Fig. 9) to verify their changes in gene expression. These proteins were predominantly uncharacterized proteins (AFUB_080630, AFUB_80700, AFUB_032940, AFUB_084250, AFUB_015640, AFUB_038990), a putative anti-microbial peptide (AFUB_085860), a putative chitin binding protein (AFUB_013970), and the major allergen aspf2 (AFUB_066690). None of the genes were considered statistically differentially expressed via DeSeq. 2 even though several differed in expression by 0.5- to 1-fold amongst the models. RT-qPCR analysis indicated all 9 genes shared similar expression patterns as the DeSeq. 2 analysis via the normalized count method. Analysis of RT-qPCR also identified 17 instances of differential gene expression (Fig. 9).

Additional analysis of the 21 annotated major allergens from *A. fumigatus* identified only 4 allergens with a count below 10 per model, 2 with a count between 10–100, and the remaining 15 with a count between 100–1000 (Supplementary Table S6). The gene encoding Aspf2 was also the most abundant transcript present for all models while several other fungal allergens were in the top 50 expressed genes across all fungal genes for a given model. Seven of 8 putative secreted highly expressed allergens from IPA expression data set were also highly expressed in the planktonic growth data set. Only Aspf2 was modestly expressed in the planktonic growth suggesting its levels may be increased due to stress or host factors *in vivo*. Global analysis of gene expression of these putative secreted proteins suggests *A. fumigatus* responds in a model specific manner with specific expression of genes unique to IPA.

## Discussion

The form of immune suppression sets the foundation for the progression of invasive pulmonary aspergillosis. Prolonged glucocorticoid treatment reduces inflammation through trans-activation, trans-repression, and direct protein-protein interactions with the glucocorticoid receptor. Ultimately these molecular phenomena result in an anti-inflammatory effect, curtailed immune signaling, decrease function of neutrophils, lymphocytes, monocytes, and macrophages (*reviewed in* ^48^). In the chemotherapeutic model, cyclophosphamide (CTX) induced leukopenia results in depletion of leukocytes and aberrant leukocyte functionality. Low dose treatment with CTX has been shown to specifically deplete CD4+ CD25+ T_regs_ and diminish their suppressive functionality as well as drive a T_h_17 response^49–51^. CTX also results in profound leukopenia; however, adoptive cell therapy post CTX treatment suggests transplanted cells are able to exploit the present immune milieu of type I interferons and cytokines (IFN-γ, IL-1β, IL-2, IL-7, IL-15, and IL-21), and promote both B- and T-lymphocyte homeostatic proliferation and activation with a predominately T_h_17 signature^49,52^. Our data agrees with these general assessments of immunosuppressive host responses as we observed statistical enrichment and elevated expression of cytokines, chemokines, and their receptors in lungs of mice in the chemotherapeutic model, but not in the steroid model. Similar to the CTX treatment model, we observed a large depletion of host leukocytes in our IPA chemotherapeutic model. Our systems level perspective proposes that the steroid models fail to appropriately elicit a robust T_h_1 and T_h_17 response, while the latent T_h_1 and T_h_17 signaling in the chemotherapeutic model cannot be effectively amplified in a timely manner due to the lack of or functionality of leukocytes. It will be interesting to determine if adoptive transfer of naïve T cells, CD8+ T cells, neutrophils or natural killer cells would respond and amplify the initial signaling events. Adoptive transfer of *ex vivo* primed dendritic cells against *A. fumigatus* has proven successful in immune suppressed murine models^53^. More promising, *ex vivo* priming of PMNs from healthy donors successfully reduced fungal infection in clinical trials involving patients receiving T-cell depleted grafts^54^.

Invasive pulmonary aspergillosis is primarily associated with a T_h_17 CD4+ T cell response and also to a lesser extent T_h_ 1 response^55–57^. Contrastingly, APBA, a hypersensitive response to chronic *A. fumigatus* exposure, induces a primarily T_h_2 and T_h_9 response resulting in a contrasting pulmonary pathology^58,59^. Murdock *et al*. (2011) and Shriener *et al*. (2012) postulated a co-evolving mixed T_h_1, T_h_2, and T_h_17 in their repeated exposure model using immune-competent C57BL/6 mice^60,61^. In the context of IPA, loss or neutralization of T_h_17 and T_h_1 drivers results in increased mortality, while decrease in T_h_2 drivers results in diminished fungal burden^40,62–64^.

Based on this importance of CD4+ T cell signaling, we incorporated an ordinary differential equation based modeling approach to gain insight into how CD4+ T cells would differentiate and respond to the observed expression of cytokines and chemokines during steroid and chemotherapeutic models of IPA. This computational modeling approach has been utilized successfully to predict differentiation and plasticity of CD4+ T cells in the gastrointestinal tract of mice in response to pharmacological activation of PPARγ that were then validated *in vivo* ^42–45^. Our *in silico* and experimental results suggest a predominately T_h_2 response in the steroid model. Though CD4+ T cells were too depleted in the chemotherapeutic model to accurately analyze, the few NK and CD8+ suggested T_h_1/T_h_2 and T_h_1/T_h_2/T_h_17 mixed responses respectively. Further dendritic cell signaling indicated a strong preference to secretion of IL-4 over IL-12 in both models. Our modeling efforts and experimental validation suggest a strong bias towards a T_h_2 mediated response as well as mitigated T_h_17 and T_h_1 response, which have both been shown to be detrimental in resolving IPA^40,62–64^. These findings also shed light onto why *ex vivo* primed dendritic cells, which were shown to secret IL-12, provided a protective role during invasive aspergillosis.

### *Gene Expression of Tlrs*, *Clecs*, *and Nlrs*

The recognition of *A. fumigatus* occurs through pattern recognition receptors including the C-type lectins Dectin-1, Dectin-2, DC-SIGN, and the toll-like receptors Tlr2, Tlr4, and Tlr9^65–73^. Tlr4 was found to be decreasing in both models from day 2 to day 3, while Tlr2 was fairly consistent in gene expression across both models on day 2 and day 3. This loss of Tlr4 expression could be due to the enhanced germination of conidia as Tlr4 is thought to be an important receptor for conidial recognition, but not hyphae^71^. Our data also identifies Tlr1, Tlr6, Tlr7, Tlr8, and Tlr13 as additional *Tlrs* expressed during IPA. Specificity of expression during IPA, but not in control inoculations, was observed for Tlr1, Tlr7, Tlr9, and Tlr13 (Tlr6 was not tested). The elevated expression of these extracellular and endocytic *Tlrs* across both models hints at successful initial recognition of *A. fumigatus*. The expression of *Tlrs* by PMNs in response to *A. fumigatus* suggests specific responses are shaped upon initial recognition^74^. As *Tlr* expression was fairly consistent between the two models it seems unlikely that loss of *Tlr* expression is a cause for fungal infection in these models.

C-type lectins, an important family of pattern recognition receptors, were relatively more divergent in expression between the chemotherapeutic and steroid models. The increased expression of Dectin-1 (Clec7a), Dectin-2 (Clec4n/Clec6a), and MINCLE (Clec4e) in the chemotherapeutic model implies activation in response to fungi by DCs, monocytes, and/or macrophages^41,75,76^. The lack of increased Dectin-1 expression in the steroid model suggest recruited neutrophils may not be fully mature or functional as glucocorticoids are known to blockade neutrophil and monocyte recruitment into tissue from the vasculature and dampen inflammatory signaling. Clec5a had similar expression patterns in both models as Dectin-1, and was not detected in the control immunosuppressed samples on day 2 or day 3. Clec5a is currently known to be an important receptor on macrophages for dengue virus resulting in down stream activation of proinflammatory cytokines^77^. Clec14a, Clec1a, and Clec2d were all decreasing in expression in both models. The decreased Clec14a transcript levels may indicate a concerted effort in both models to induce angiogenesis and diminish cell-to-cell adhesion^78^. Clec1a is known to be a receptor found inside myeloid cells and is up regulated in response to TGF-β^79,80^. The decreased Clec1a expression may be an important marker for lack of NK cells as these populations are expected to be depleted in the chemotherapeutic model. Clec2d is associated with inhibition of osteoclasts, and an important in facilitating missing-self recognition by Natural Killer Cells^81^. Clec4d (MCL) was found elevated in the steroid model, was increasing in expression in the chemotherapeutic model, and was not detected in the control samples. Recently, Clec4d has been shown to be a key molecule in anti-mycobacterial host defense^82^. Both Clec4d and Clec5a are of high relevance as they contribute to the recognition of tuberculosis and dengue virus respectively^77,82^. It is not yet known if these two C-type lectins play important roles in the recognition and defense against *A. fumigatus* similar to that of Dectin-1; however, both their expression is dependent on inoculation with *A. fumigatus* in comparison to control mock inoculations.

The *Nlr* family of genes are comprised of approximately 20+ members in humans and mice. Nlrs are thought to function broadly as sensors for pathogen encoded molecular patterns or danger associated molecular patterns and play crucial roles in infectious and immune-mediated diseases. Seven of these genes were expressed or differentially expressed during the steroid or chemotherapeutic model of IPA. Recognition of *A. fumigatus* results in the stimulation of the NLRP3 inflammasome^83^. NOD1 and NOD2 are also upregulated in response to *A. fumigatus* ^84,85^. Our results identify the novel expression or differential expression of 7 *Nlrs* (Nlrc3, Nlrc4, Nlrc5, Nlrp1a, Nlrp3, Nlrp12, and Nlrx1) during mouse models of IPA. Of particular interest is the model specific expression of Nlrp12 and Nlrc5. Nlrp12 was found to be highly elevated in the steroid model, but marginally expressed in the chemotherapeutic. It has been shown that Nlrp12 is an important negative regulator of pro-inflammatory cytokines, NF-κB and MAPK signaling in response to *Brucella abortus* ^86^. Nlrp12 also attenuates colonic inflammation through the promotion of commensal bacterial growth^87^. Our results show highly elevated Nlrp12 expression only in the presence of *A. fumigatus* and steroid treatment. This suggests the immune system is primed for activation of Nlrp12 in response to *A. fumigatus* under steroid treatment. Nlrc5 was decreasing in expression in the steroid model relative to chemotherapeutic. *Nlrc5*−/− mice have strongly impaired MHC class I- mediated CD8+ T cell activation post challenge with *Listeria monocytogenes*, suggesting the expression of Nlrc5 is important for CD8+ T cell defense response^88^. CD8+ T cells are believed to be amongst an important class of lymphocytes for defense against *A. fumigatus* ^89,90^. The steady expression of Nlrx1 in the steroid model and chemotherapeutic model suggests Nlrx1 is relevant to both models of IPA and provides an avenue to link immune responses with metabolism. Recently, it has been shown loss of Nlrx1 results in increased proliferation and differentiation of CD4+ T cells into a pro-inflammatory state^91^. This occurs through decreased responsiveness to immune check point pathways such as those mediated by PD-1 and CTLA-4, enhanced lactate dehydrogenase signaling, and increased expression of HIF-1α in normoxic and hypoxic environments. The decreased expression of Nlrx1 during IPA in both models in comparison to mock inoculations suggests this immune check point is down-regulated in response to *A. fumigatus* in these immune suppressive host contexts. Additional dissection of Nlrs will provide new mechanistic insight into how these genes contribute to IPA disease outcomes.

### Host Metabolism

Our global and specific analysis of metabolic differences between the steroid and chemotherapeutic model suggested distinct expression patterns of metabolic pathways. The activation of the TCA cycle, urea cycle and their intermediaries may suggest a robust production of reactive nitrogen species in the chemotherapeutic model via Nos2 and Nos3. Nos2 expression is also an indicator of macrophage activation^92^, while Nos3 is associated with endothelial nitric oxide production^93^. The increase in Nos2, an inducible nitric oxide synthase, has been associated with M1 inflammatory phenotype in macrophages and is a requirement for effector/pro-inflammatory responses to bacteria and tumors^92^. However, the greater expression of arginase (Arg1) suggests a more tolerogenic response^94^. Elevated arginase expression has been associated with wound healing, limits the inflammatory potential and proliferation of effector T cells^95,96^, and increased the severity of HIV and visceral leishmaniasis^97^. Response to and killing of *A. fumigatus* conidia by alveolar macrophages requires the production of reactive oxygen species^98^. Reactive nitrogen species could not be measured or identified during these challenge experiments. Recently, a detailed series of *in vitro* experiments clearly demonstrate *Blastomyces dermatitidis* actively inhibiting nitric oxide production^99^. Though the inhibitory factor was not identified, the authors ruled out blockade of arginine transport and reduction in Nos2 gene expression as mitigating agents. Our study reveals moderate expression of a number of arginine transports and elevated expression of Nos2. No published study to our knowledge indicates loss of *Nos2* results in increased virulence or fungal load during IPA. It will be essential to determine if the Arg1 and Nos2 expression along with arginine transporter expression occurs in distinct populations, or if Nos2 function is inhibited at a post transcriptional level^100^.

Other aspects of the global metabolic gene expression analysis suggested both models are reducing pyruvate to lactate via Ldha in response to anaerobic respiration or to recycle NADH and maintain NADH/NAD+ balance. This is in agreement with observations of hypoxic domains at sites of infection during both chemotherapeutic and steroid models of IPA^31^. Intriguingly, myeloid HIF-1α is essential for protection against IPA and these data suggest one potential mechanism is a requirement for HIF-1α to maintain metabolic homeostasis^101^. The decreased expression of fatty acid synthesis genes in the steroid and particularly the chemotherapeutic model suggests diminished cell proliferation^46^. The increase in transcripts associated with fatty acid degradation in the steroid model proposes a preference toward a non-inflammatory and tolerogenic status^46^. The elevated expression of phosphoenolpyruvate carboxykinase Pck1 in the steroid model would also facilitate gluconeogenesis and a means for glucose independent cell growth and metabolic stress resistance^102^. This stress resilience is not apparent in the chemotherapeutic model as we found evidence for a broken TCA cycle. Our analysis of metabolism suggests these processes may play important roles in shaping the immune suppression, and understanding these mechanisms may yield new therapeutic targets to mitigate host damage. These findings also provide clues to the metabolic environment the invading fungus is exposed too during IPA in the respective models. An increase in lactate and processes associated with gluconeogenesis likely suggests the fungus is exposed to a hypoxic microenvironment and alternative carbon sources as disease progresses as previously has been suggested^31,103^. Moreover, it has been shown that loss of the AcuM gluconeogenesis associated transcriptional regulator in *A. fumigatus* decreases fungal virulence^104^.

Though our data provides novel insight into the metabolic and immune response during IPA, an important caveat emerged on determining from what cell type gene expression changes were occurring. The lung is made up of three major compartments and each compartment is composed of a variety of cell types. Recruitment of leukocytes adds complexity, as these diverse cell types make up a small percentage of total cell population. The use of RNA-Seq, on total lung tissue provides an averaged response on gene expression across all cell types, and would inherently better capture gene expression changes in more abundant cell types, such as airway cells, alveolar unit cells, and pulmonary vascular cells. Consequently, our RNA-Seq study is inherently biased for capturing gene expression changes from more abundant cell types, and does not retain or factor in gene expression changes for a given cell type.

These experimental limitations became more evident when we analyzed fungal transcripts during models of IPA. Fungal transcripts accounted for a small percentage of total reads and varied by model. The generally decreased number of fungal transcripts in the steroid model and increased number of fungal transcripts in the chemotherapeutic model masked and skewed fungal gene expression changes when analyzed through a FPKM based strategy. Only through a normalization of counts, which factors in the total number of counts for a given sample, were we able to look at fungal gene expression changes while factoring in the relative amount of fungus. RT-qPCR of fungal genes validated these changes in gene expression using the count-based method, but not the FPKM based method. The advent of single-cell sequences methods would remove many of the challenges associated with RNA-Seq analysis of complex tissue samples comprised of small heterogeneous cell populations of interest, and the recent development of a reversible fixative method to preserve gene expression changes would facilitate such single cell studies^105–107^. Notwithstanding, our data set provides an overview of the gene expression changes occurring in the host in response to *A. fumigatus* in the setting of different immune suppression regimens.

### Secreted proteins of *A. fumigatus*

The role of secreted proteins from fungi and oomycetes in facilitating symbiosis is clearly evident^108,109^. A number of such proteins function in extracellular spaces, while others are translocated into host cells. When clustered by protein sequence similarity, *A. fumigatus* generated unique and diverse sized tribes. The predicted secreted proteins of *A. fumigatus* also clustered into unique groupings associated with both murine model and day of infection based on gene expression. Overlaying of gene expression data on clustered tribes identified both instances of temporal gene expression redundancy and specificity amongst tribe members. The notion of temporal and organ specific gene expression of secreted proteins is well understood for a number of plant pathogenic fungi and oomycetes^110–112^. Here we showcase the temporal specificity of gene expression from 9 different putative secreted proteins. Of note are the expression of a putative secreted anti-microbial peptide and putative secreted chitin binding protein. Microbial derived chitin binding proteins have been shown to play major role in virulence by several plant pathogenic fungi^113–115^. The increased gene expression of a putative anti-microbial peptide in both the chemotherapeutic and steroid models may suggest an effort to neutralize microbial communities that may also be vying for growth in an immunocompromised host or more likely that environmental conditions in the lung induce expression of fungal AMPs. For example, hypoxia is known to induce production of AMPs from host cells in a HIF dependent mechanism^116^. Importantly, however, these AMP gene functions are dependent on annotation and not currently experimentally supported for this fungal protein. The gene encoding the major fungal allergen AspF2 was found to be the highest expressed gene in 3 of 4 models and third highest in the other. AspF2 and other allergenic proteins have been shown to stimulate a predominantly T_h_1 response in healthy human PBMCs, while inducing a T_h_2 response in PBMCs from individuals with ABPA^117^. The elevated expression of these allergens suggests they may function as an important peptide based stimulatory factors for the observed T_h_1 response in our models, though the mechanism and host receptors remain unknown. Finally, the differential expression of several components of the gliotoxin biosynthesis cluster also provides additional evidence of context specific responses associated with a given model of IPA. Forthcoming molecular and computational analysis of these tribes and expression groupings will provide insight into the mechanism by which these secreted proteins and secreted metabolites facilitate pathogenesis in a context specific manner.

This RNA-Seq study provides a systems level understanding of the murine immune and metabolic signaling processes in response to established *A. fumigatus* infections under different immune suppressing regimens. Our findings suggest conserved processes such as induction of host anaerobic respiration via Ldha and HIF signaling pathways in response to presumably lower oxygen previously reported to be an important component of IPA microenvironments^31^. However, the identification of a reduced TCA cycle in the chemotherapeutic model showcases key divergences exist between the models. Analysis of immune signaling pathways suggests both models are biased towards a T_h_2 cell based response and beneficial aspects of early cytokine signaling towards T_h_1 and T_h_17 responses are near completely depleted. Furthermore, this RNA-Seq study identifies the specific expression of several novel *Clecs*, *Tlrs* and *Nlrs* previously unassociated with IPA. Analysis of putative fungal secreted proteins reinforces the notion of temporal and model specific activation of secreted proteins indicating *A. fumigatus* is responding to contrasting environments through specific molecular mechanisms that remain to be functionally interrogated. Overall, the data set generated herein provides fertile ground for testing biological significance of the observed changes in host and fungal gene expression during IPA in two immunologically distinct murine models of infection.

## Materials and Methods

### Mouse models of IPA

Standardized steroid and chemotherapeutic mouse models of invasive pulmonary aspergillosis were used for this study^31^. For RNA-Seq experiments, female mice (CD1), 6–8 weeks of age, were housed 4 animals per cage in a controlled environment in the Dartmouth CCMR facility consisting of HEPA filtered air, autoclaved food *ad libitum*. Steroid model mice received a single dose of Kenalog (Bristol-Myers Squibb Company, Princeton, NJ, USA) injected subcutaneously (s.c.) at 40 mg/kg 1 day prior to inoculation. Chemotherapy model mice received intraperitoneal (i.p.) injections of cyclophosphamide (Baxter Healthcare Corporation, Deerfield, IL, USA) at 175 mg/kg 2 days prior to inoculation and a subcutaneously (s.c.) injection of Kenalog at 40 mg/kg 1 day prior to inoculation. Mice were inoculated via the intranasal route with 2 × 10^6^ *A. fumigatus* strain CEA10 (also called CBS144.89) conidia per mouse on day 0. Mock samples were inoculated with sterile phosphate buffer saline. Mouse lungs were harvested from each animal on day 2 and 3 post inoculation. All animal studies were carried out in strict accordance with the recommendations in the Guide for the Care and Use of Laboratory Animals of the National Institute of Health, and were approved by the respective Virginia Tech and Dartmouth IACUCs.

### Extraction of RNA, library preparation, and sequencing

Whole mouse lung tissue was lyophilized and processed through triazol extraction followed by Qiagen clean-up. Library preparation was performed using the TruSeq RNA preparation kit (Illumina, FC-122-1001/1002) from purified RNA (100 ng-1 μg of total RNA with RIN ≥ 8.0). Generated libraries were validated using Agilent 2100 Bioanalyzer and quantified using Quant-iT dsDNA HS Kit (Invitrogen) and qPCR. Twelve individually indexed cDNA libraries were pooled and designed to acquire 30 million paired end reads (60 M reads) per sample using an Illumina HiSeq-2500 (Supplementary Table S1).

### RNA-Seq data analysis

Read quality control was initially performed by FastQC. Adaptor sequences were trimmed and filtered based on phred score (>33) and length (>36) using Trimmomatic-0.35^118^. One of the replicate for the LD2 sample did not pass quality control due to overall low base call quality and was dropped, leaving LD2 sample with two biological replicates. Three remaining samples (LD3, SD2, SD3) all had three biological replicates. A master GTF was created using the GRCm38 mouse genome build and the Af1163 genome scaffolds. Filtered pair-ended RNA-Seq reads were mapped to the merged reference genome using the splice-aware short read mapping tool TopHat 2.1.0 with Bowtie2 2.2.7^119^. RNA sequencing data was then reassessed by FASTQC and QualiMap^120^. Abundance estimation and differential expression analysis for the reference genes and transcripts were performed using Cufflinks 2.2.1 and visualized in part via CummRbund^121^. Based on histogram distribution of mouse gene expression a gene is considered expressed when its FPKM > 1. Murine genes were considered differentially expressed when q-value < 0.05 and the log_2_-fold change was > 1. Counts for fungal genes were normalized based on both housekeeping genes and overall model normalization using DESeq. 2^122^. A given fungal genes was considered differentially expressed when either of the two compared normalized count were ≥ 10 and p_adj_ < 0.05. A given fungal genes was considered expressed when the normalized count was ≥ 10.

### Functional Computational Analysis

Groupings of mouse differentially expressed genes were analyzed for functional classification using PantherDB.org online server^35^. These groupings were then analyzed for functional enrichment of Gene Ontology Mouse specific Biological Processes terms using the BINGO app^32^ in Cytoscape^33^. Groupings were further analyzed using the ReactomeFIViz^34^ in Cytoscape. A list of IRGs was generated from innateDB^123^ and a list of metabolic genes was generated from KEGG^36^. These genes were independently clustered based on FPKM values using GENE-E [http://www.broadinstitute.org/cancer/software/GENE-E/index.html]. IRG clusters were then analyzed in Cytoscape and the Reactome FIVIZ app. Groupings of metabolic genes were overlaid onto the KEGG Metabolic Pathway through the online web portal^36^ and through Cytoscape using KEGGScape^124^ to read in KEGG KGML files for a given pathway. Groupings of *A. fumigatus* differentially expressed genes based on normalized counts were analyzed for enrichment (Hypergeometric test, Benjamini & Hochberg False Discovery Rate (FDR) correction, p < 0.05) by FungiFun2 using both FunCat and GO ontologies^125^.

A list of candidate fungal secreted proteins from the predicted proteins of A. fumigatus 1163 were generated using SignalP4.0^126^ and further filtered using TMHMM2.0^127^. Proteins were then compared at the amino acid level using command line Blast+^128^. Blast results were pooled and analyzed by the Markov clustering algorithm with various I cut-offs from 14 to 60^129^. These I14 clusters were then analyzed and visualized using Cytoscape. Temporal clusters were determined based on the FPKM values of the predicted secreted proteins from *A. fumigatus*.

### Computational modeling

Simulations of CD4+ T cell differentiation were run using a previously described model^42^. The model was implemented within the Complex Pathway Simulator (COPASI) software as a system of ordinary differential equations^43^. RNA-Seq data for cytokines *(IL-18*, *IL-12*, *IFNγ*, *IL-21*, *IL-6*, *IL-17*, *IL-23*, *IL-4*, *TGF-β*, *IL-2*, *IL-10*) and receptor *(IL-18r*, *IL-12r*, *IFNγr*, *Il-6r*, *IL-17r*, *IL-23r*, *IL-4r*, *TGF-βr*, *IL-2r*, *IL-10r)*, and transcription factors (*Tbx21*, *Gata3*, *Foxp3*, *Rorc*) was compiled in triplicate for steroid model day 2, steroid model day 3, and chemotherapeutic model day 3 and in duplicate for chemotherapeutic model day 2. The model was then calibrated for time course simulation using a particle swarm algorithm for the optimization of parameter fitting for the steroid and chemotherapeutic models separately. Time course simulations used an LSODA method for the deterministic solution of the system.

### RT-qPCR

Purified total RNA (500 ng) was used to construct a cDNA library (qScript cDNA Synthesis Kit, Quantas) in a total volume of 20 μL using the following parameters: 22 °C for 5 min; 42 °C for 30 min; 85 °C for 5 min; hold 4 °C. The cDNA was further diluted with 20 μL of supplied nuclease free water. Template cDNA, 2 μL, was used for a qPCR reaction (20 μL) and run in triplicate per sample using 250 nM primers (final concentration) and SsoAdvanced Universal SYBR green Supermix (Bio-Rad) using the following polymerization parameters: Activation: 30 sec at 95 °C; Amplification: 41 cycles of: 10 sec at 95.0 °C, 30 sec at 60 °C; Melting Analysis 60 °C to 95 °C with 0.5 °C increments every 5 sec. A standard curve using custom gblocks (IDTDNA) as template was used to assess PCR efficiency (Supplementary Table S7). Primers were designed using the IDT RealTime PCR Tool or prior published primer pairs (Supplementary Table S7). Differences in gene expression was calculated using the ΔΔCT method. β-actin was utilized a control for mouse gene expression studies. TefA and β-tubulin expression were used as a control for fungal gene expression studies.

### Characterization of leukocyte populations

For immunological studies, female mice (C57BL/6), 6–8 weeks of age were housed 3–5 animals per cage in a controlled environment in the Virginia Tech Vivarium consisting of HEPA filtered air, autoclaved food *ad libitum*, and purified filtered water. Steroid model mice received a single dose of cortisone acetate at 250 mg/kg injected subcutaneously on day 3 prior to inoculation. Chemotherapy model mice received an intraperitoneal injection of cyclophosphamide, at 250 mg/kg, on day 3 prior to inoculation and a subcutaneous injection of cortisone acetate at 250 mg/kg on day 3 prior to inoculation. Mice were inoculated with *A. fumigatus* (CEA10) via aerosolization as described in *Sheppard et al*.^130^. Mouse lungs and BALF fluid was collected on day 3 post inoculation.

Cells were obtained from lung tissue and bronchoalveolar lavage fluid (BALF). BALF was generated through cannulation of the trachea postmortem using a gavage needle and washed three times with 1 mL of room-temperature PBS that was then pooled and treated with protein transport inhibitor (BD #554724). Lungs tissue was processed and also treated with protein transport inhibitor. Red blood cells were removed through hypotonic lysis and filtration. Remaining cells were resuspended in 1 mL of PBS + 5% fetal bovine serum +0.09% sodium azide at a concentration 6 × 10^5^ cells per well in a 96 well plate. Plated cells were treated with antibody cocktail (anti-CD16/anti-CD32) to remove non-specific binding. Cells were then incubated with antibodies targeting extracellular receptors for 20 min at 4 °C (Supplementary Table 7). Cells were then fixed and permeabilized for intracellular staining. Cells were then incubated with intracellular antibodies in permeabilization buffer. Cell phenotyping was performed after live cell analysis (FSC vs SSC), doublet exclusion (FSC-H vs FSC-W and SSC-H vs SSC-W) and positive selection of CD45+ events. Approximately 30,000 CD45+ cells were acquired and subsequently analyzed for cell phenotyping. Flow cytometry experiments were conducted using a custom LSRII flow cytometer (Becton Dickinson). All experiments were independently repeated, with a N = 8.

### Data availability statement

The raw read data and computed count files for this study are available in the NCBI Gene Expression Omnibus repository under the accession number GSE104290. Datasets generated during and/or analyzed for this study are available as Supplementary Files. Additional information is readily available from the corresponding author with reasonable request.

## Electronic supplementary material

Supplementary zip

Supplementary Figures

Supplementary Table 1

Supplementary Table 2

Supplementary Table 3

Supplementary Table 4

Supplementary Table 5

Supplementary Table 6

Supplementary Table 7
