# Supplementary material for: Modulation of Immune Signaling and Metabolism Highlights Host and Fungal Transcriptional Responses in Mouse Models of Invasive Pulmonary Aspergillosis
Source: Sci Rep. 2017 Dec 6;7:17096. doi: 10.1038/s41598-017-17000-1 (PMC5719083; doi:10.1038/s41598-017-17000-1)
Supplement: Supplementary file 2 — Supplementary Figures [file 41598_2017_17000_MOESM2_ESM.pdf]

Modulation of Immune Signaling and Metabolism Highlights Host and Fungal Transcriptional Responses in Mouse Models of Invasive Pulmonary Aspergillosis

Shiv D. Kale[1*], Tariq Ayubi[1], Dawoon Chung[2,5], Nuria Tubau-Juni[1], Andrew Leber[1], Ha X. Dang[1,3], Saikumar Karyala[1], Raquel Hontecillas[1], Christopher B. Lawrence[4], Robert A. Cramer[2], Josep Bassaganya-Riera[1]

[1]Nutrional Immunology and Molecular Medicine Laboratory, Biocomplexity Institute of Virginia Tech. Blacksburg, VA 24061 USA

[2]Department of Microbiology and Immunology, Geisel School of Medicine at Dartmouth, Hanover, NH 03755 USA

[3]Current Address: McDonnell Genome Institute at Washington University, St. Louis, MO 63108

[4]Department of Biological Sciences, Virginia Tech. Blacksburg, VA 24061 USA

[5]Current Address: National Marine Biodiversity Institute of Korea, Seochun-gun, 33662, Republic of Korea

Corresponding Author [*]: Shiv D. Kale (sdkale@vt.edu)

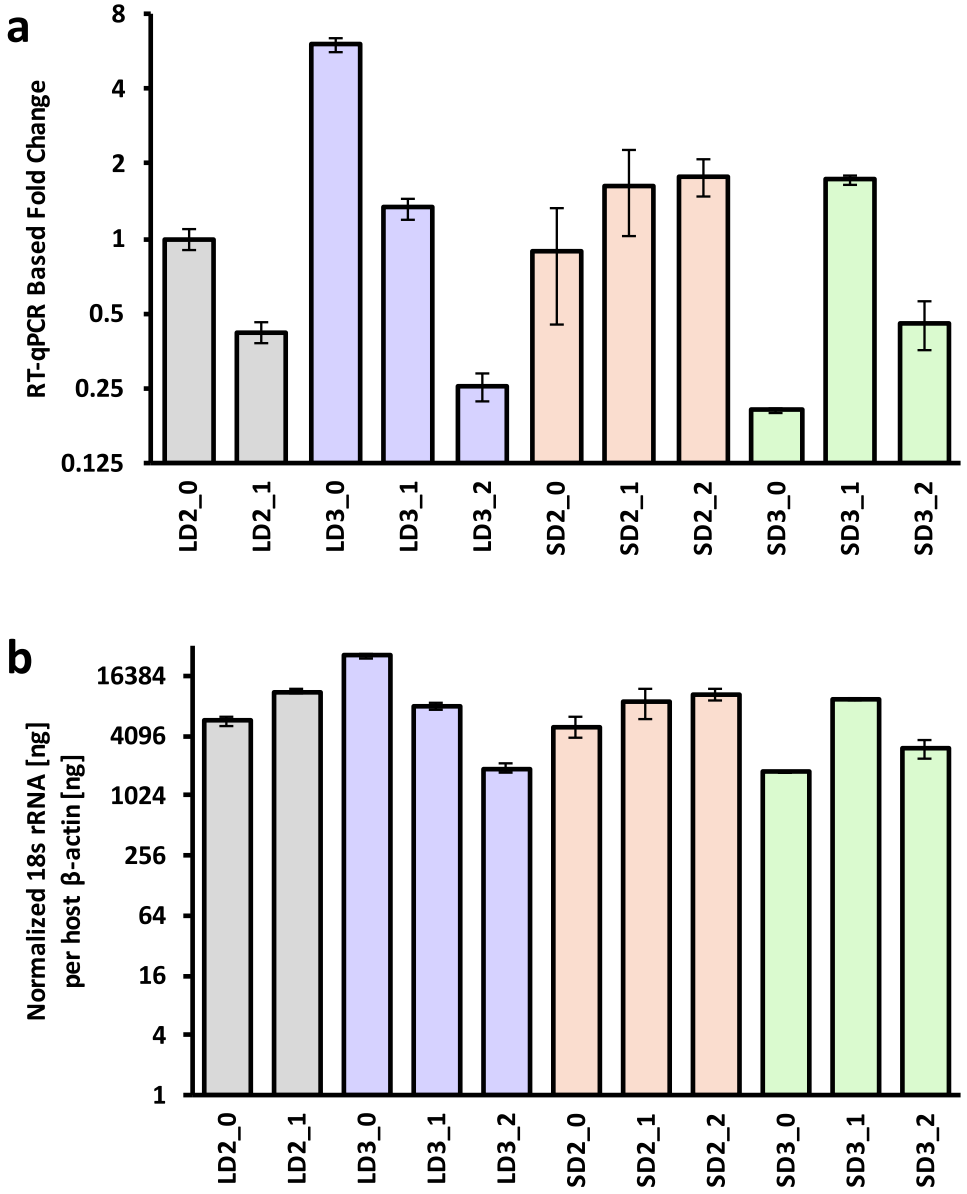

Supplementary Figure S1. Measurement of *A. fumigatus* 18s rRNA during chemotherapeutic and steroid models of invasive pulmonary aspergillosis on day 2 and 3 post inoculation. (A) RT-qPCR based fold change of 18s rRNA normalized to host β-actin mRNA, and then further normalized to LD2_0 sample (comparator). (B) Normalized 18s rRNA (ng) in respect to host β-actin (ng). _# denotes specific sample replicate. Each sample was measured in triplicate. Standard error of the sample mean is presented. Steroid model, S; Chemotherapeutic model, L; Day 2, D2; Day 3, D3.

a

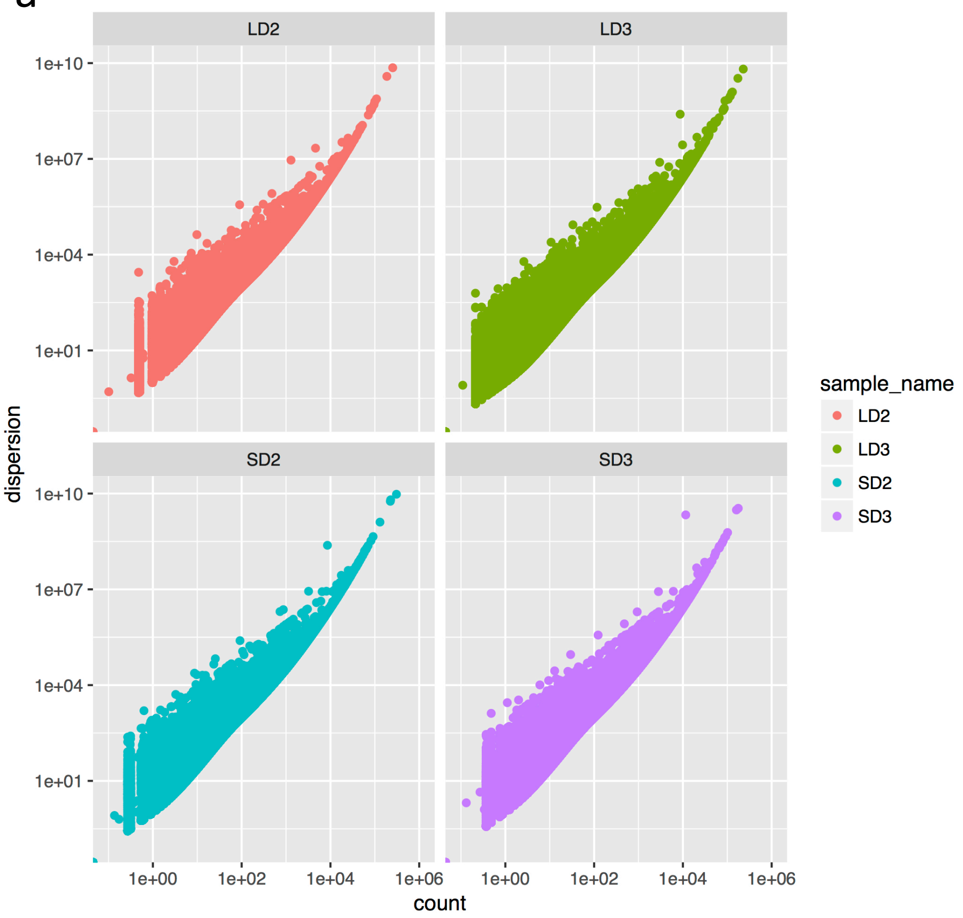

LD2
LD3
SD2
SD3
1e+10
1e+07
1e+04
1e+01
dispersion
1e+00
1e+02
1e+04
1e+06
count
sample_name
LD2
LD3
SD2
SD3

b

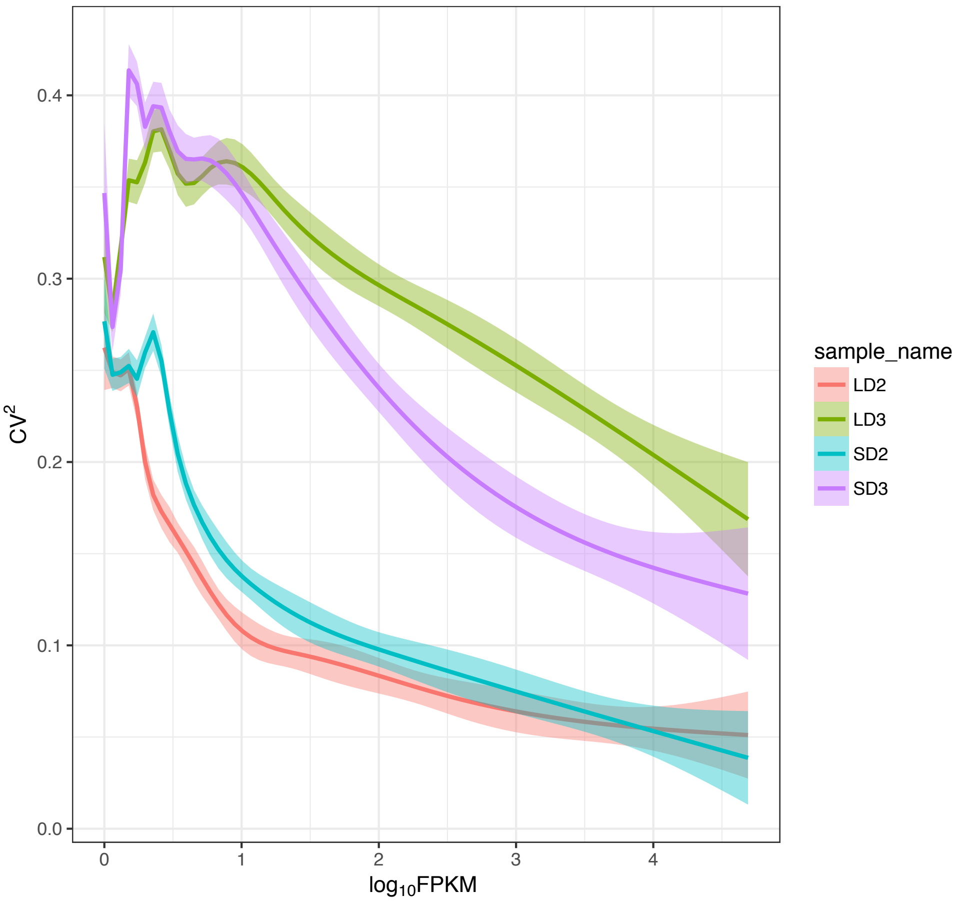

0.4
0.3
0.2
0.1
0.0
CV^2
0
1
2
3
4
$\log_{10}$FPKM
sample_name
LD2
LD3
SD2
SD3

c

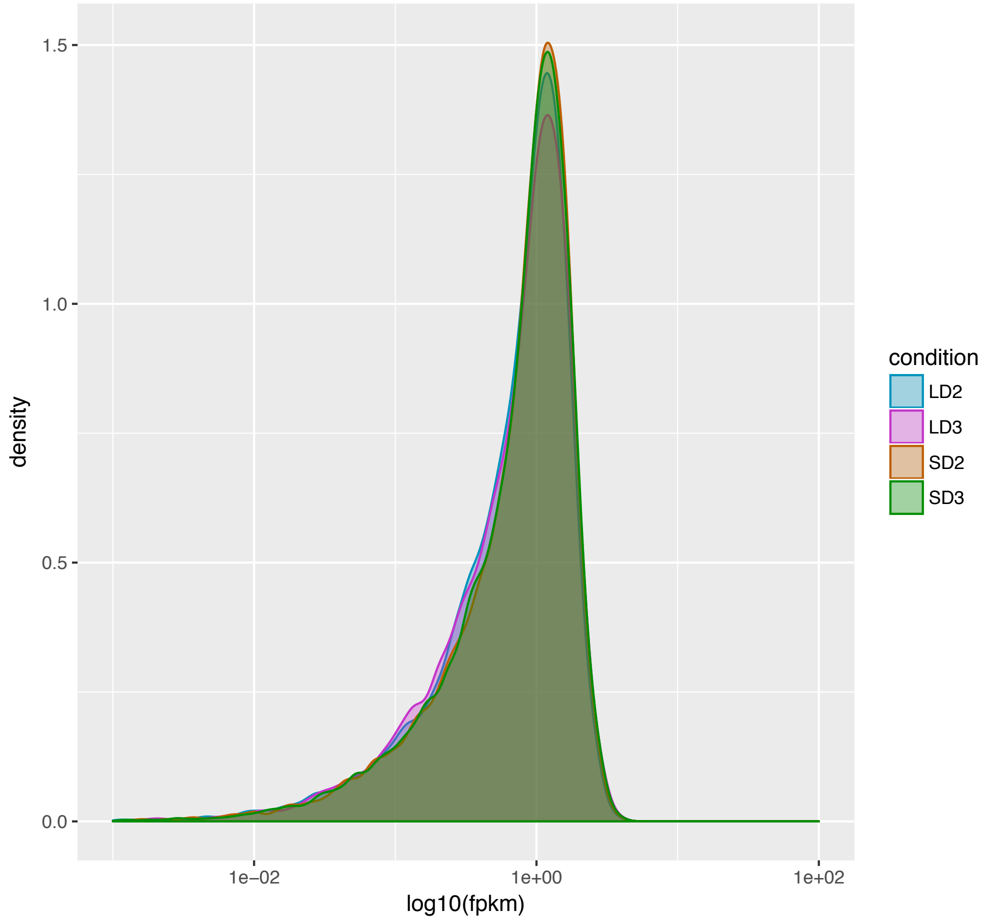
1.5
1.0
0.5
0.0
density
1e−02
1e+00
1e+02
log10(fpkm)
condition
LD2
LD3
SD2
SD3

d

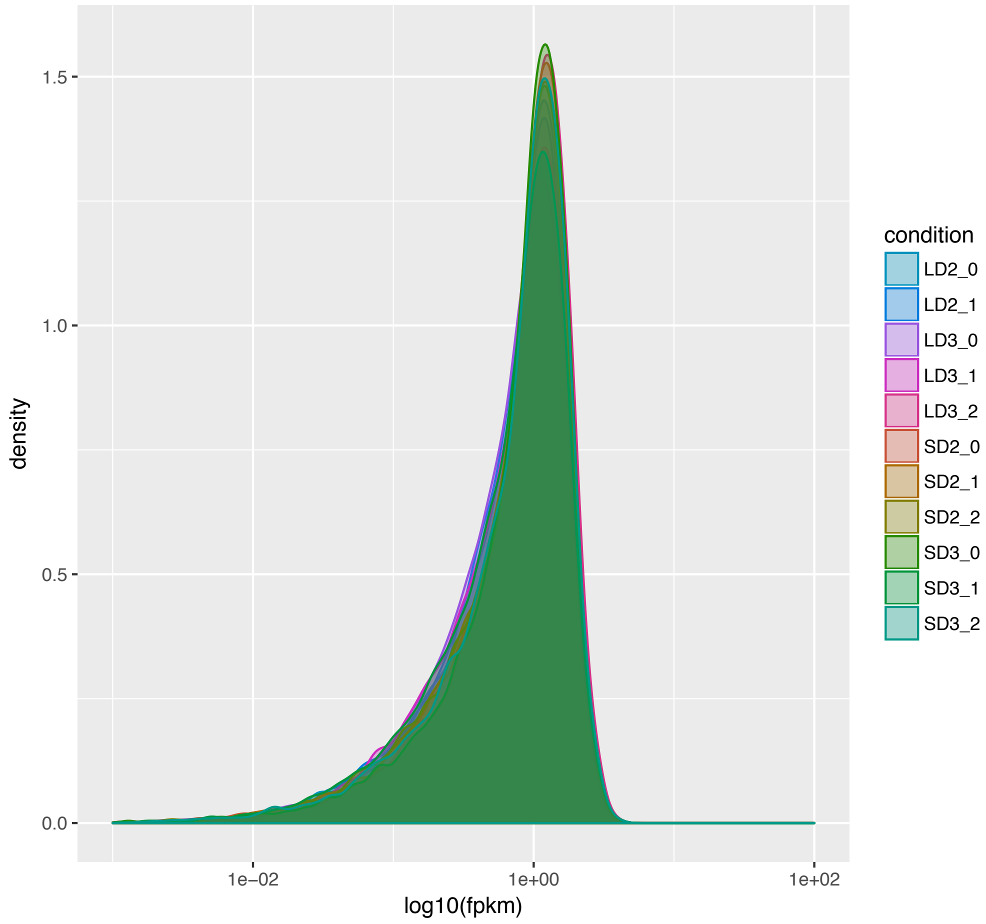
1.5
1.0
0.5
0.0
density
1e−02
1e+00
1e+02
log10(fpkm)
condition
LD2_0
LD2_1
LD3_0
LD3_1
LD3_2
SD2_0
SD2_1
SD2_2
SD3_0
SD3_1
SD3_2

e

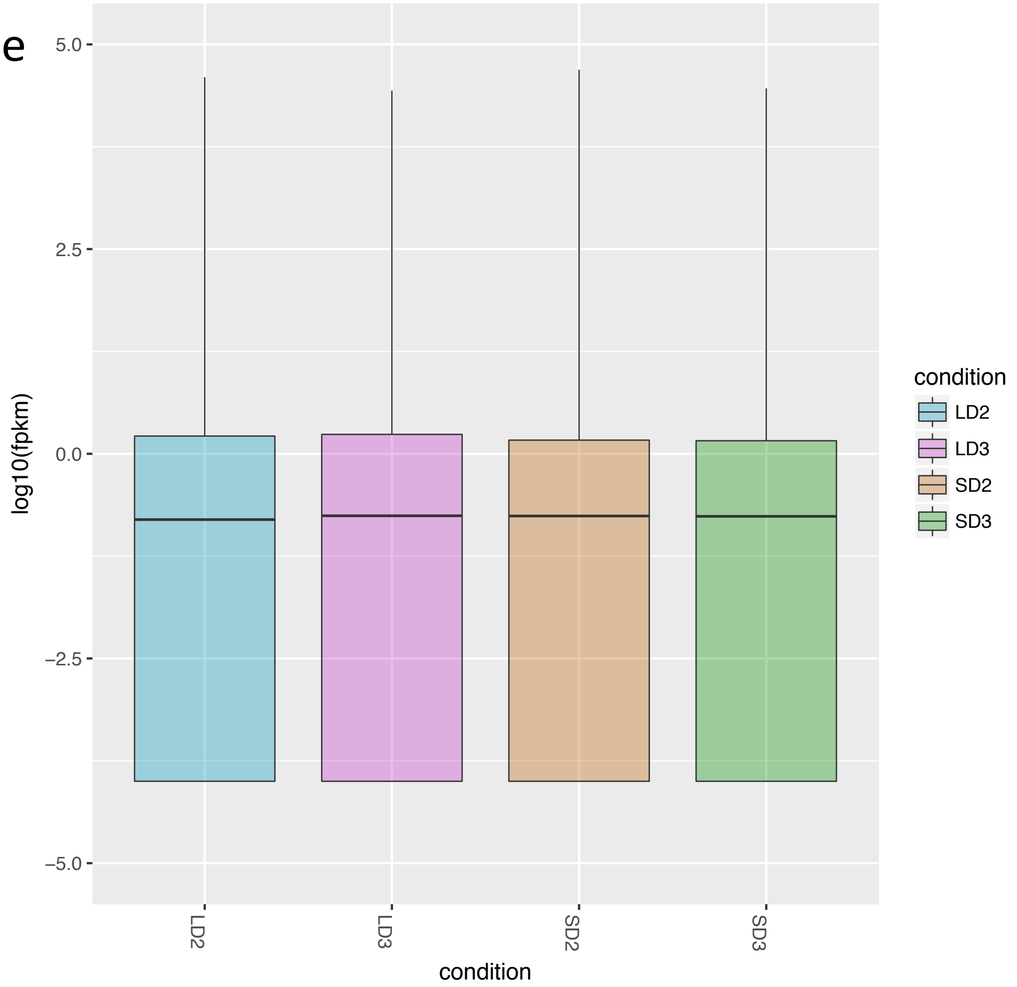
5.0
2.5
0.0
−2.5
−5.0
log10(fpkm)
LD2
LD3
SD2
SD3
condition
condition
LD2
LD3
SD2
SD3

f

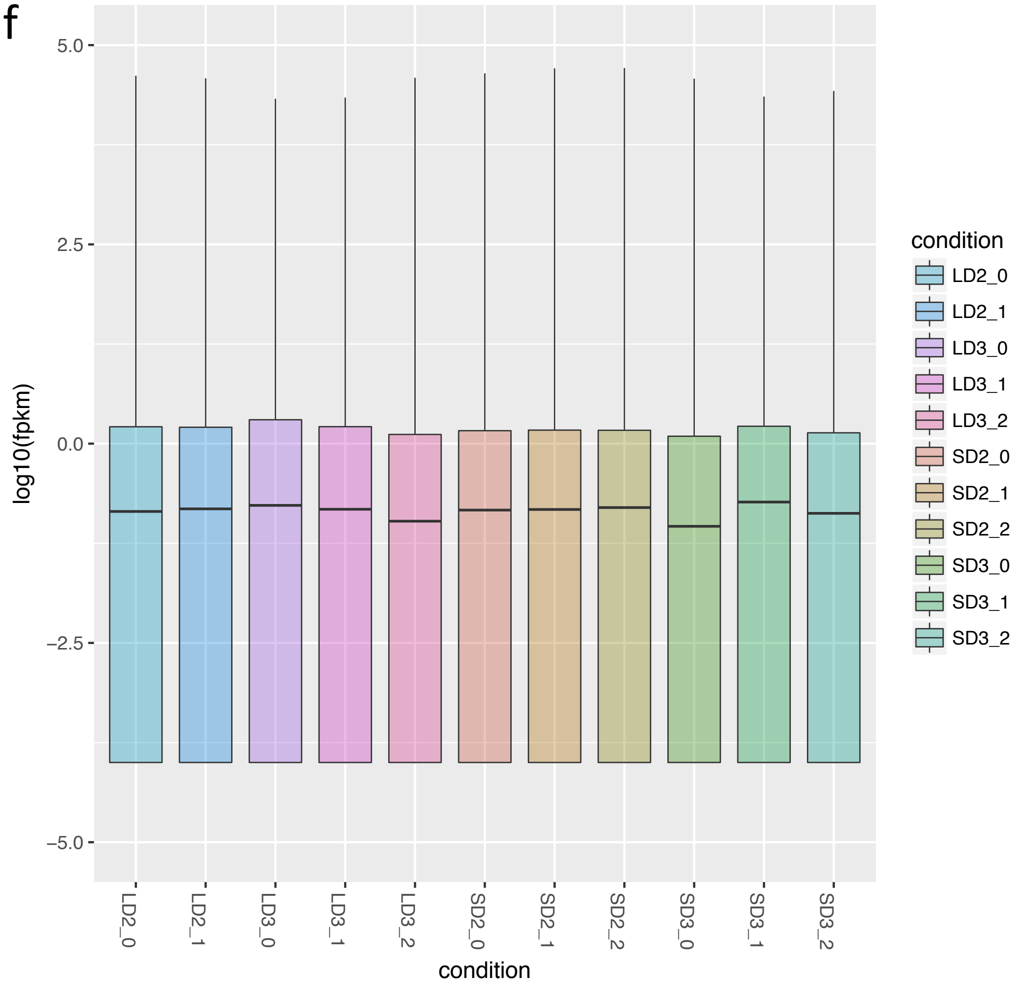
5.0
2.5
0.0
−2.5
−5.0
log10(fpkm)
LD2_0
LD2_1
LD3_0
LD3_1
LD3_2
SD2_0
SD2_1
SD2_2
SD3_0
SD3_1
SD3_2
condition
condition
LD2_0
LD2_1
LD3_0
LD3_1
LD3_2
SD2_0
SD2_1
SD2_2
SD3_0
SD3_1
SD3_2

g

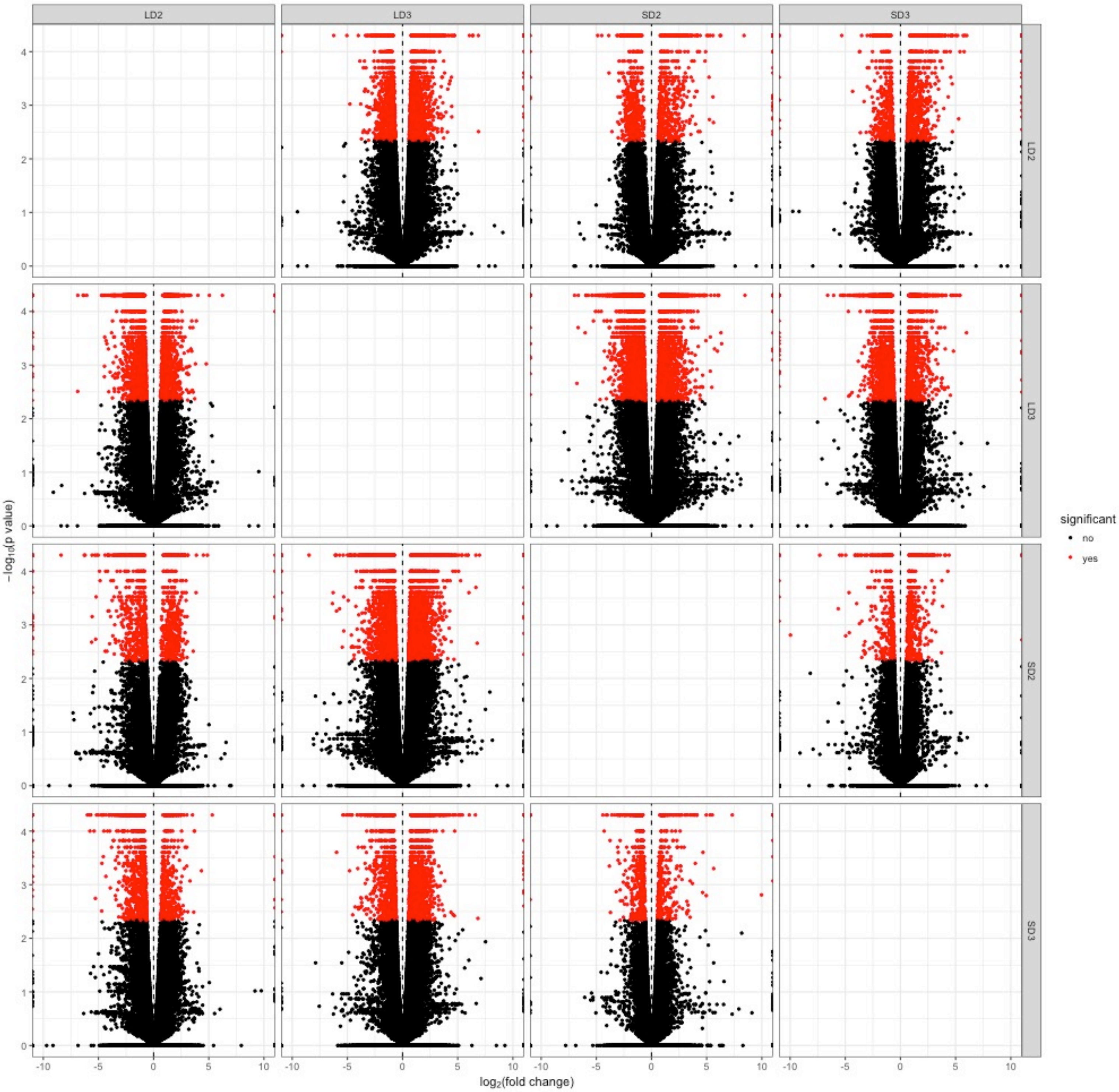
LD2
LD3
SD2
SD3
−log10(p value)
log2(fold change)
significant
no
yes

h

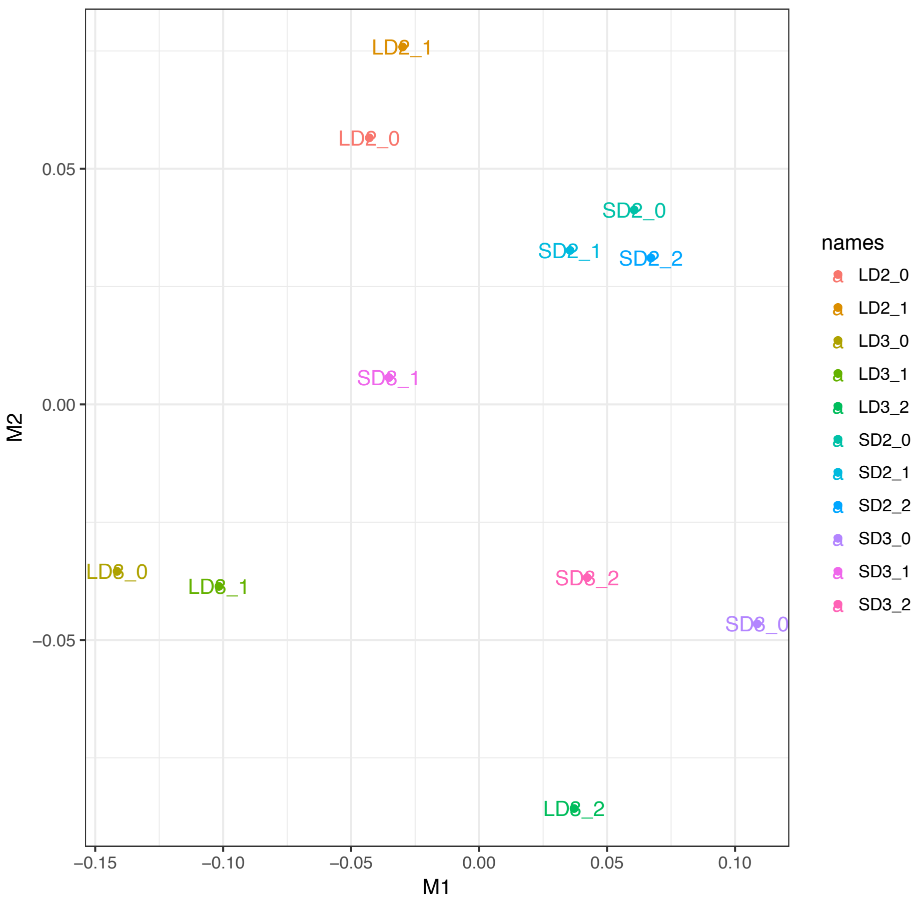
LD2_1
LD2_0
SD2_0
SD2_1
SD2_2
SD3_1
LD3_0
LD3_1
SD3_2
SD3_0
LD3_2
0.05
0.00
−0.05
M2
−0.15
−0.10
−0.05
0.00
0.05
0.10
M1
names
LD2_0
LD2_1
LD3_0
LD3_1
LD3_2
SD2_0
SD2_1
SD2_2
SD3_0
SD3_1
SD3_2

i

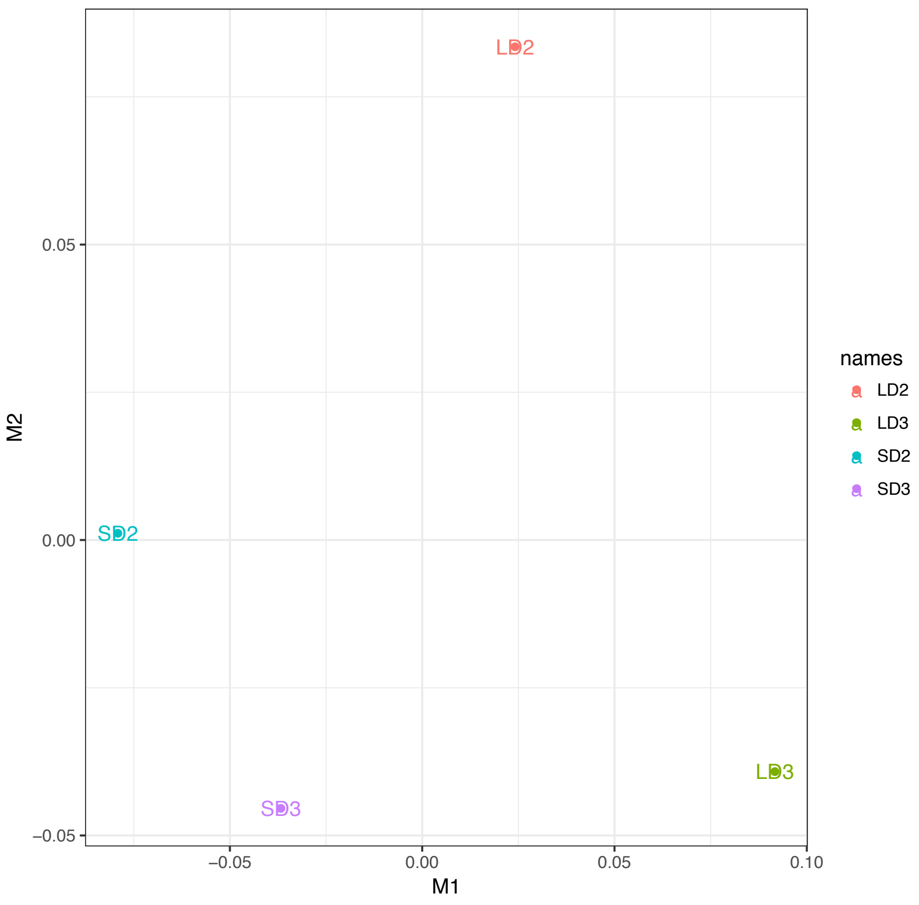
LD2
SD2
SD3
LD3
0.05
0.00
−0.05
M2
−0.05
0.00
0.05
0.10
M1
names
LD2
LD3
SD2
SD3

j

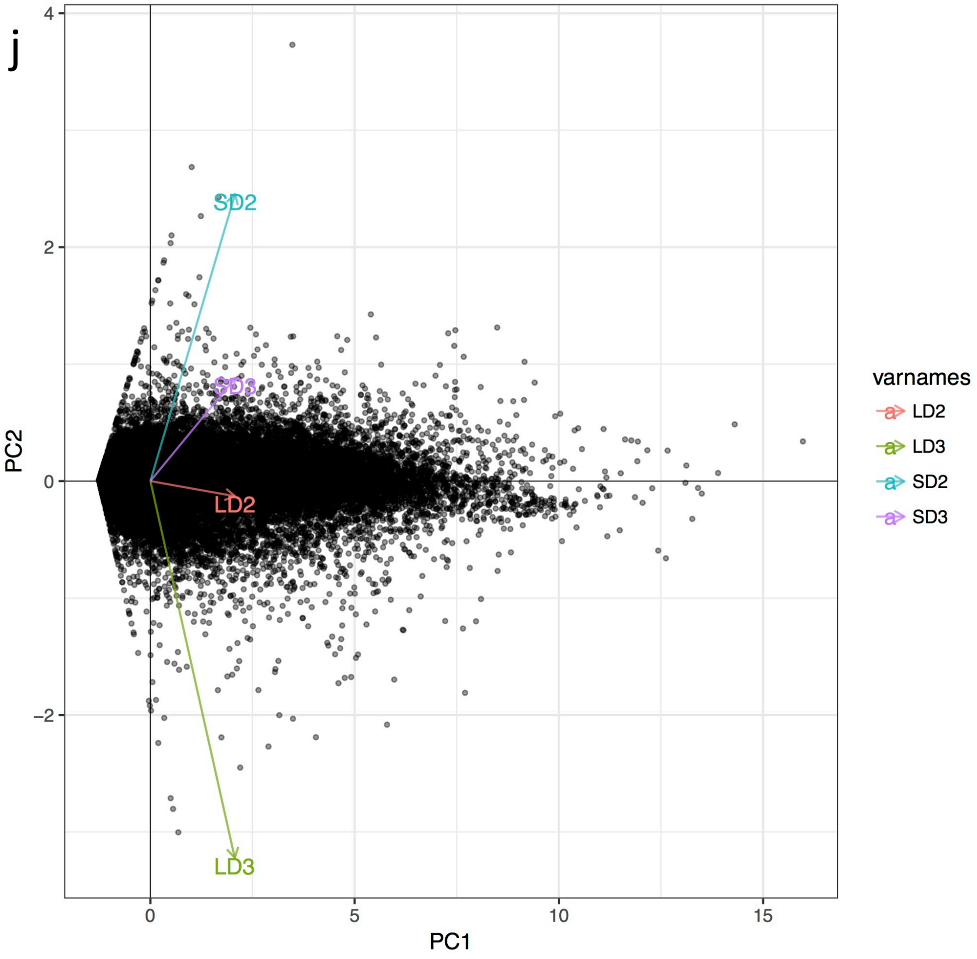

SD2
SD3
LD2
LD3
PC1
PC2
varnames
LD2
LD3
SD2
SD3

k

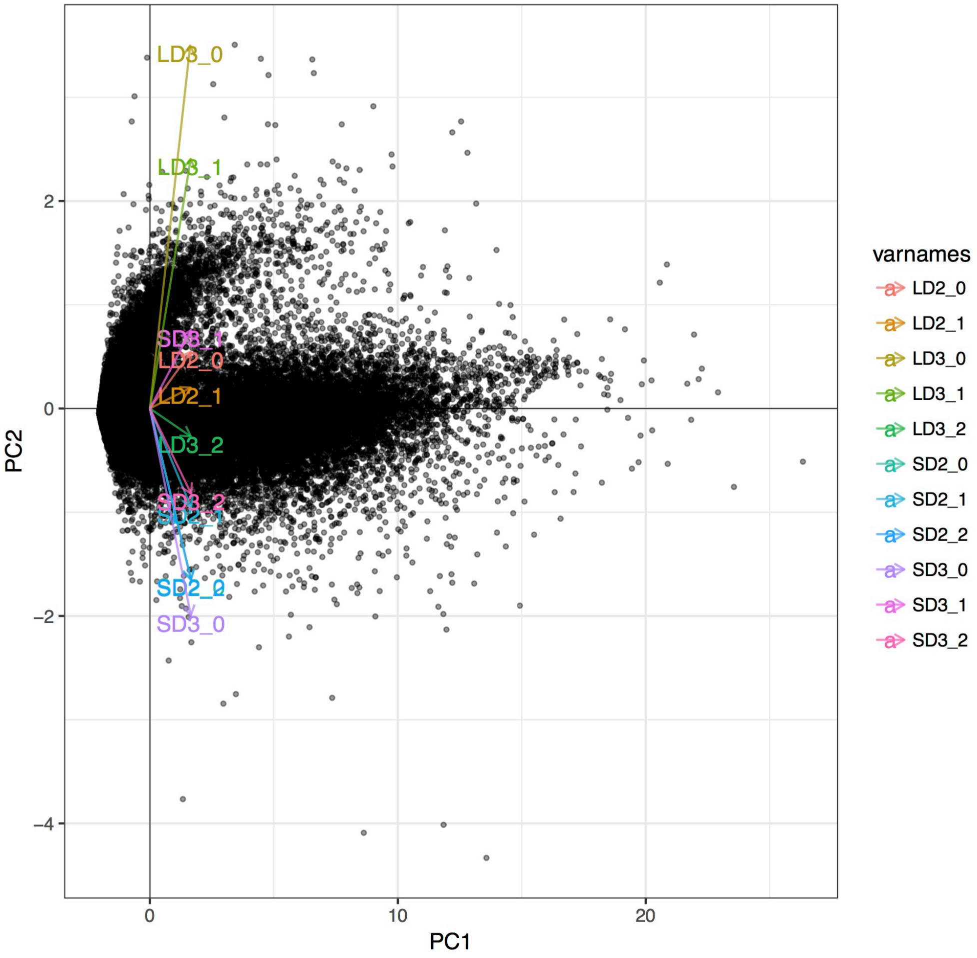

LD3_0
LD3_1
LD2_0
LD2_1
LD3_2
SD2_2
SD3_0
PC1
PC2
varnames
LD2_0
LD2_1
LD3_0
LD3_1
LD3_2
SD2_0
SD2_1
SD2_2
SD3_0
SD3_1
SD3_2

Supplementary Figure S2. Standardized cummeRbund output for global statistics and quality control of RNA-Seq data. (a) Dispersion parameter plotted against counts = fragments per kilobase of transcript per million mapped reads (FPKM) for each gene in a given model. (b) squared coefficient of variation plotted against log_{10} FPKM for each model. Density plot of log_{10} FPKM for all genes in a (c) given model and (d) samples replicates. (e) Box plot of FPKM distributions for a given model and (f) sample replicates. (g) volcano plot of log_2 fold change versus significance (-log_{10}(p-value)). Multidimensional scaling (MDS) plot for gene-level features for (h) each model and (i) sample replicates. Principal component analysis (PCA) plot for gene-level features for (j) each model and (k) sample replicates. Steroid Model, S. Chemotherapeutic Model, L. Day 2, D2. Day 3, D3.

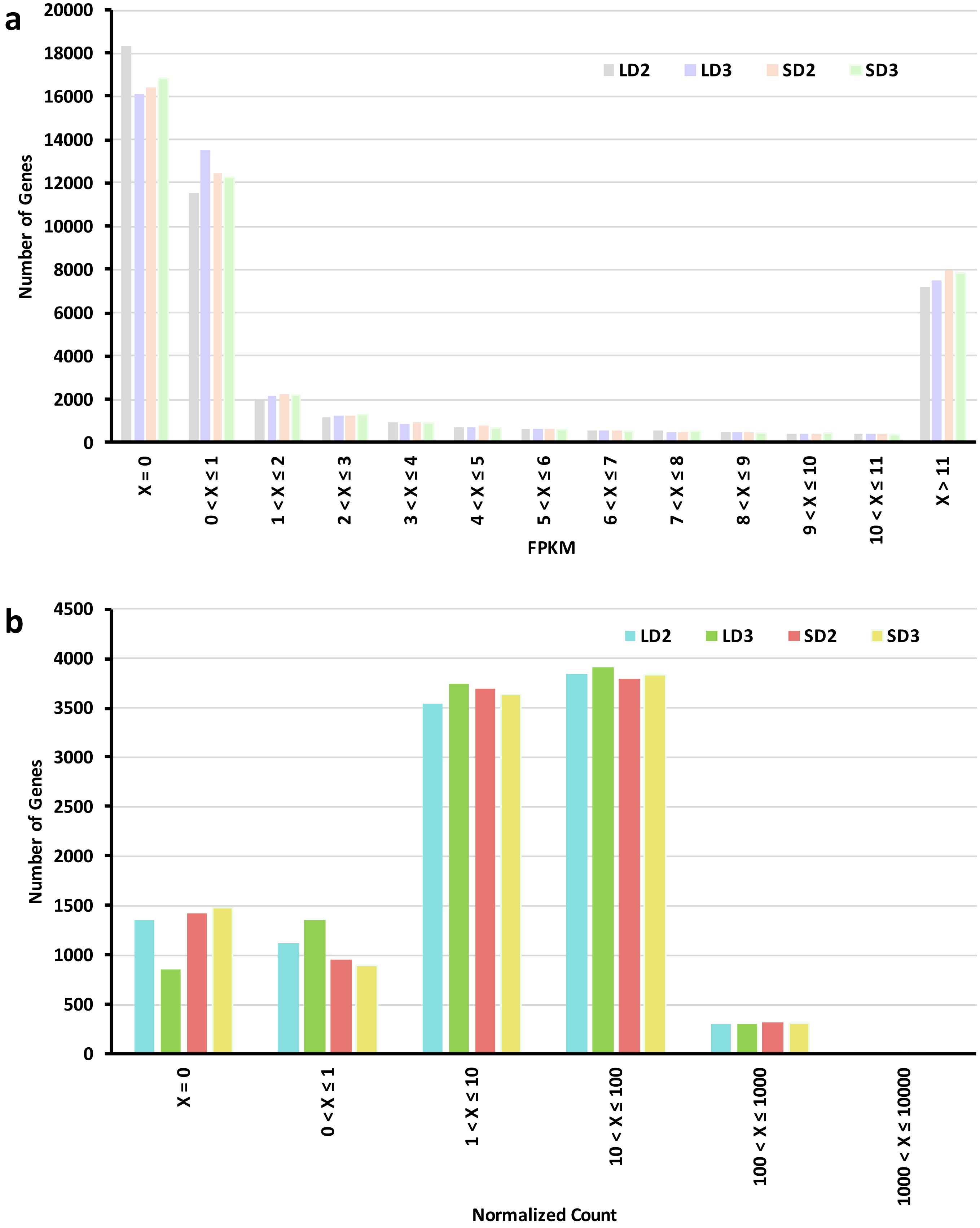

Supplementary Figure S3. Histogram distribution of the number of (a) mouse and (b) *A. fumigatus* genes within a given range of transcript expression for the chemotherapeutic and steroid models of invasive pulmonary aspergillosis on day 2 and 3 post inoculation. (a) mouse gene expression is presented in fragments per kilobase million (FPKM). (b) *A. fumigatus* gene expression is presented in normalized counts. A given bin's range is indicated by the x-axis. Steroid Model, S; Chemotherapeutic Model, L; Day 2, D2; Day 3, D3.

a

Network	Genes	Identified Nodes	Edges	E/N Ratio	Modules (> 2 Nodes)
LD2-3 Up	1173	472	2024	4.288	18
LD2-3 Down	640	159	239	1.503	18
SD2-3 Up	244	67	136	2.030	7
SD2-3 Down	172	24	25	1.042	4
LD2 Enriched	76	18	33	1.833	11
SD2 Enriched	446	70	81	1.157	10
LD3 Enriched	427	130	257	1.977	9
SD3 Enriched	505	99	128	1.293	3
cDEGS Up	160	44	72	1.636	6
cDEGS Down	57	5	3	0.600	1

b

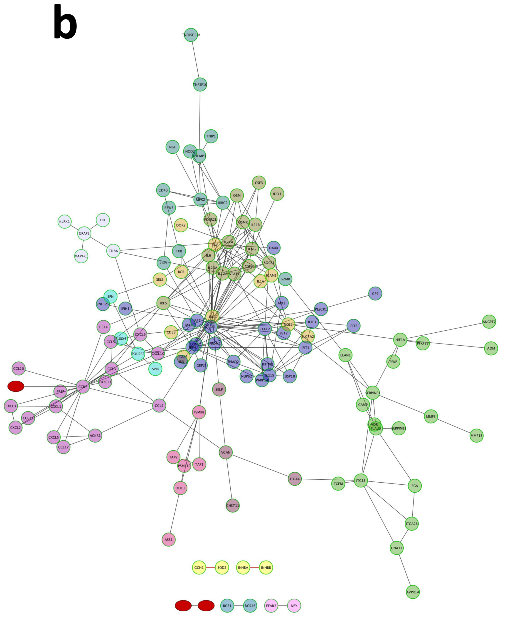

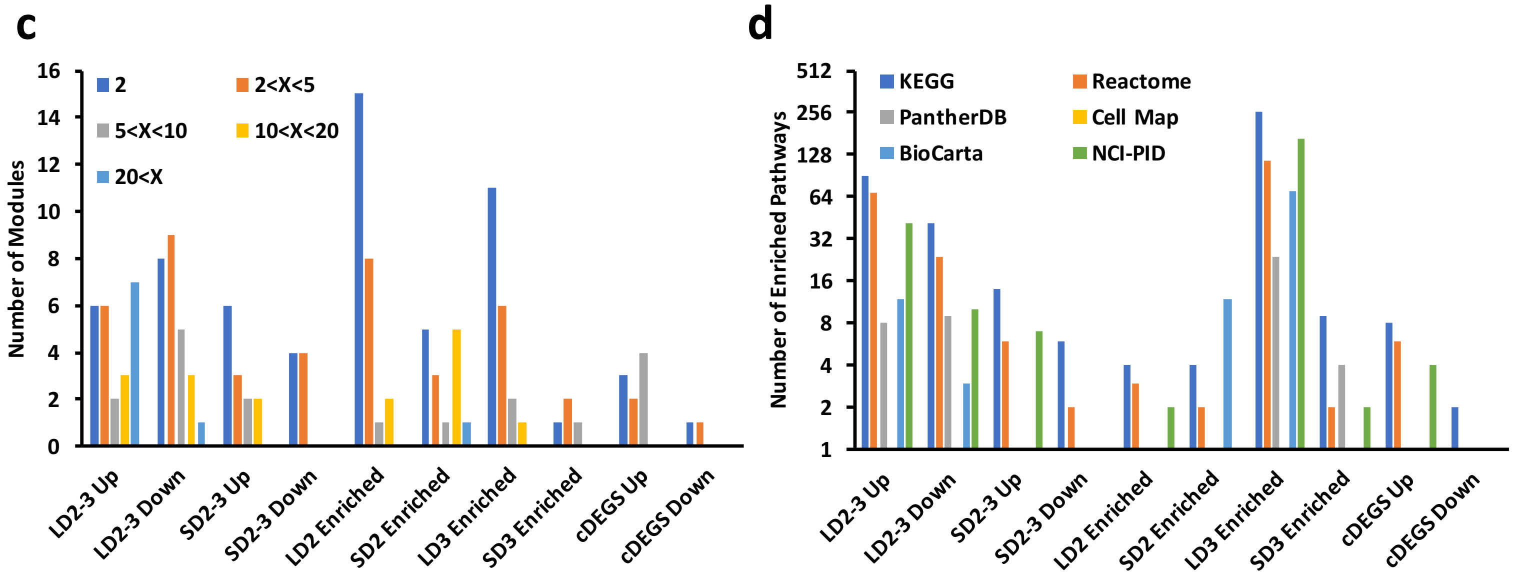

Supplementary Figure S4. Enrichment of known and curated protein-protein interaction networks and pathways (Reactome[34]) by mouse differentially expressed gene groupings identified amongst the chemotherapeutic and steroid models of invasive pulmonary aspergillosis on day 2 and 3 post inoculation. (a) Summary table of network properties: number of genes in grouping, number of genes identified in protein-protein interaction network, number of edges connecting nodes in a identified network, ratio of edges to nodes, and number of modules (peer-reviewed, assembled, and curated protein interaction networks) greater than 2 members identified in a given network. (b) Representative protein interaction network based upon differentially expressed genes enriched in the chemotherapeutic model in comparison to the steroid model on day 3. (c) bar graph of the number of modules of a given size for a given enriched network. (d) Number of enriched pathways ($p < 0.001$) from curated databases by a given identified network. LD, chemotherapeutic model; SD, steroid model; 2-3, from day 2 to 3; Up, differentially expressed genes increasing in expression; Down, differentially expressed genes decreasing in expression; Enriched, differentially expressed genes of higher transcript expression for the identified model in comparison to the alternate model for a given day; cDEGs Up, differentially expressed genes increasing in expression from day 2 to day 3 in both models; cDEGS Down, differentially expressed genes decreasing in expression from day 2 to day 3 in both models.

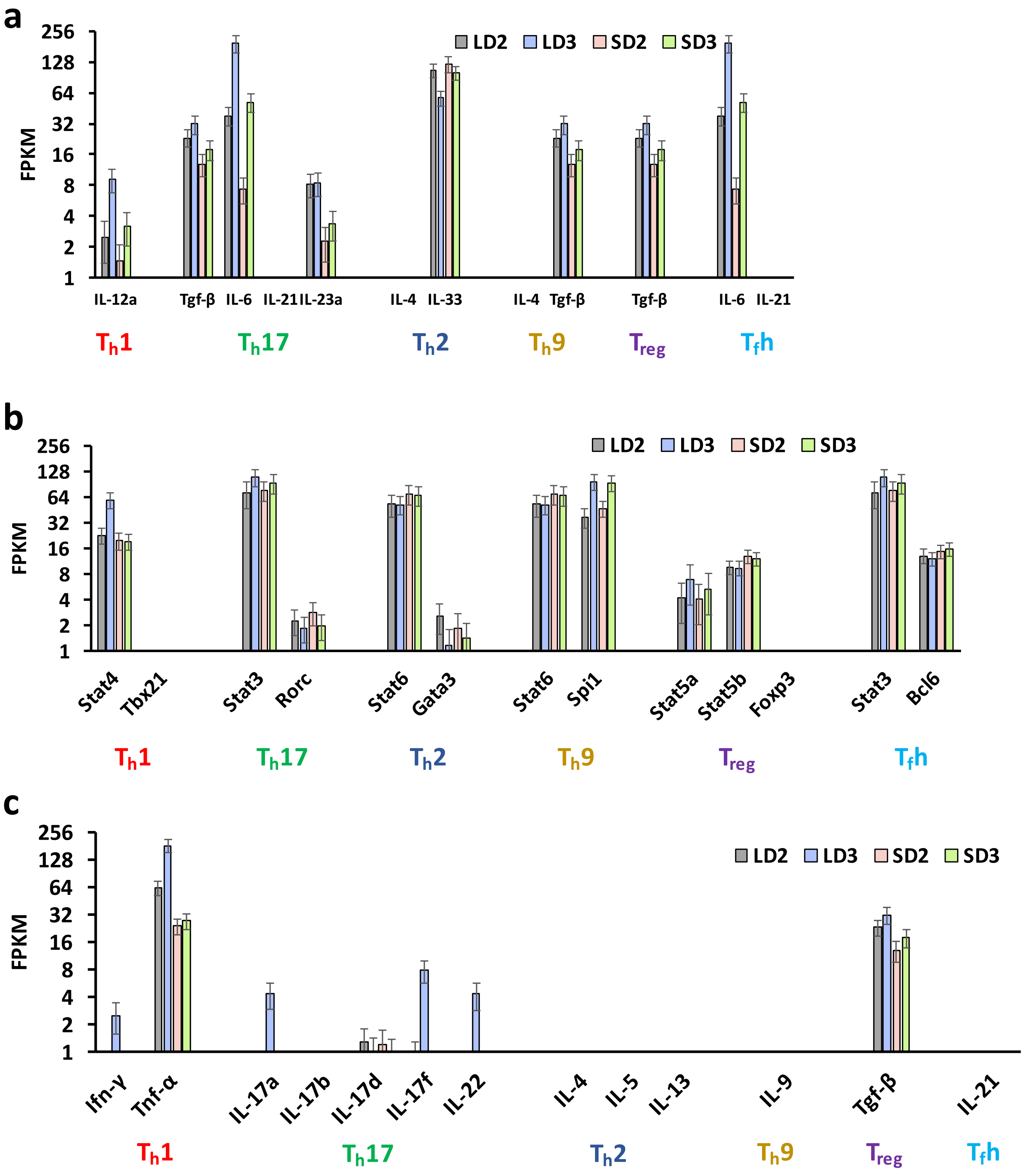

Supplementary Figure S5. Gene expression values (FPKM) for a subset of (a) early cytokines, (b) transcription factors, and (c) secondary stage cytokines expressed in the chemotherapeutic and steroid models of invasive pulmonary aspergillosis on day 2 and 3 post inoculation. Secondary x-axis labels are indicative of association with a given T-helper cell response (T_h). T_{reg}, T regulatory helper cell. T_fh, T follicular helper cell. Steroid Model, S; Chemotherapeutic Model, L; Day 2, D2; Day 3, D3. Standard error of the sample mean is presented.

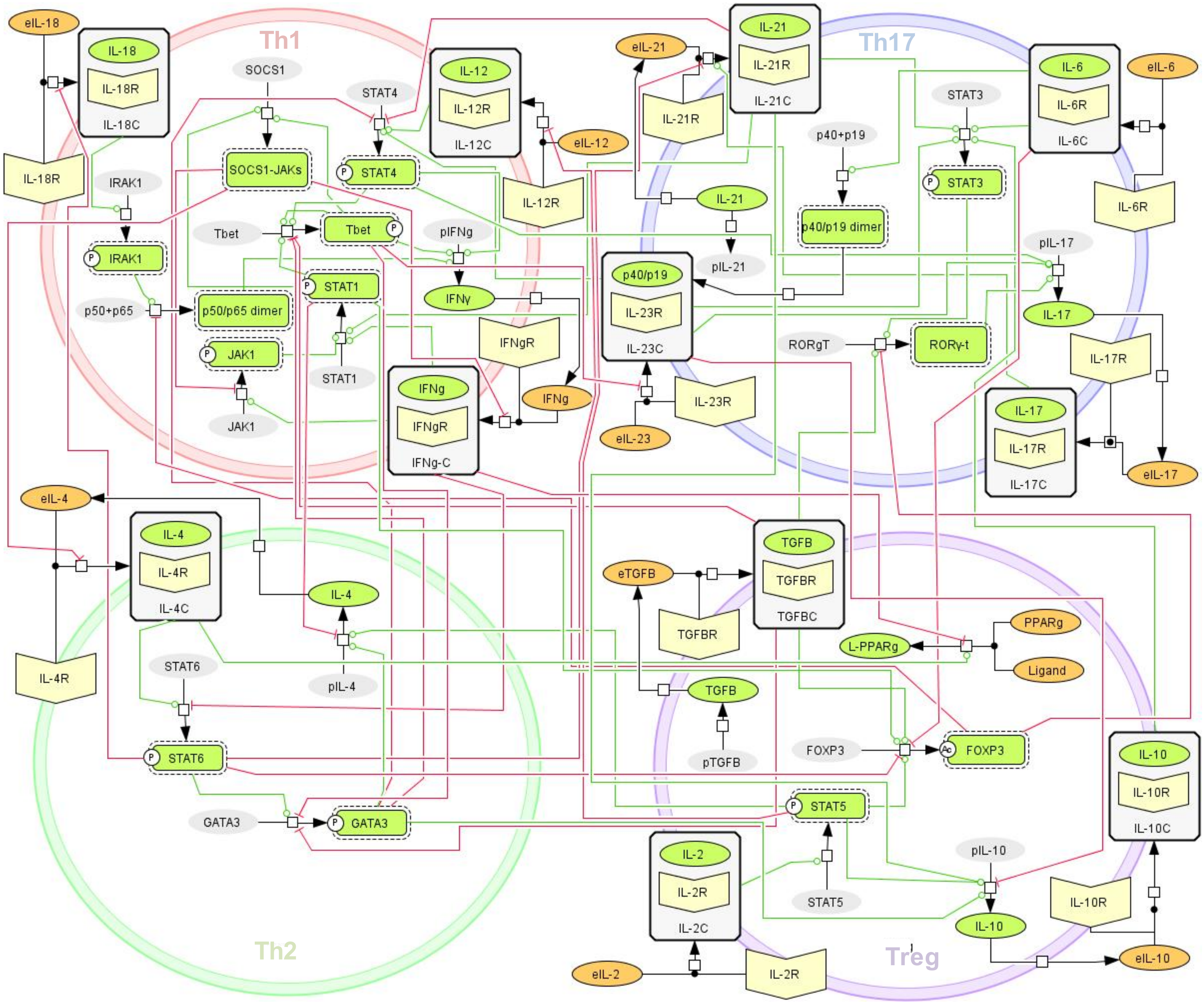

Supplementary Figure S6. Wiring Diagram for modeling of CD4+ T cell differentiation. Extracellular immune signaling molecules are denoted by orange circles and located outside of cells, while counterpart receptors are denoted by cream colored hexagons at the cell membrane. Internalized immune signaling molecules are denoted by green circles. Transcription factors are denoted by gray circles, will modified transcription factors are denoted by green rectangles. Directed green edges indicate a positive impact on a reaction, while directed red edges indicate a degree of inhibition on a reaction.

a LD2 to LD3 Up

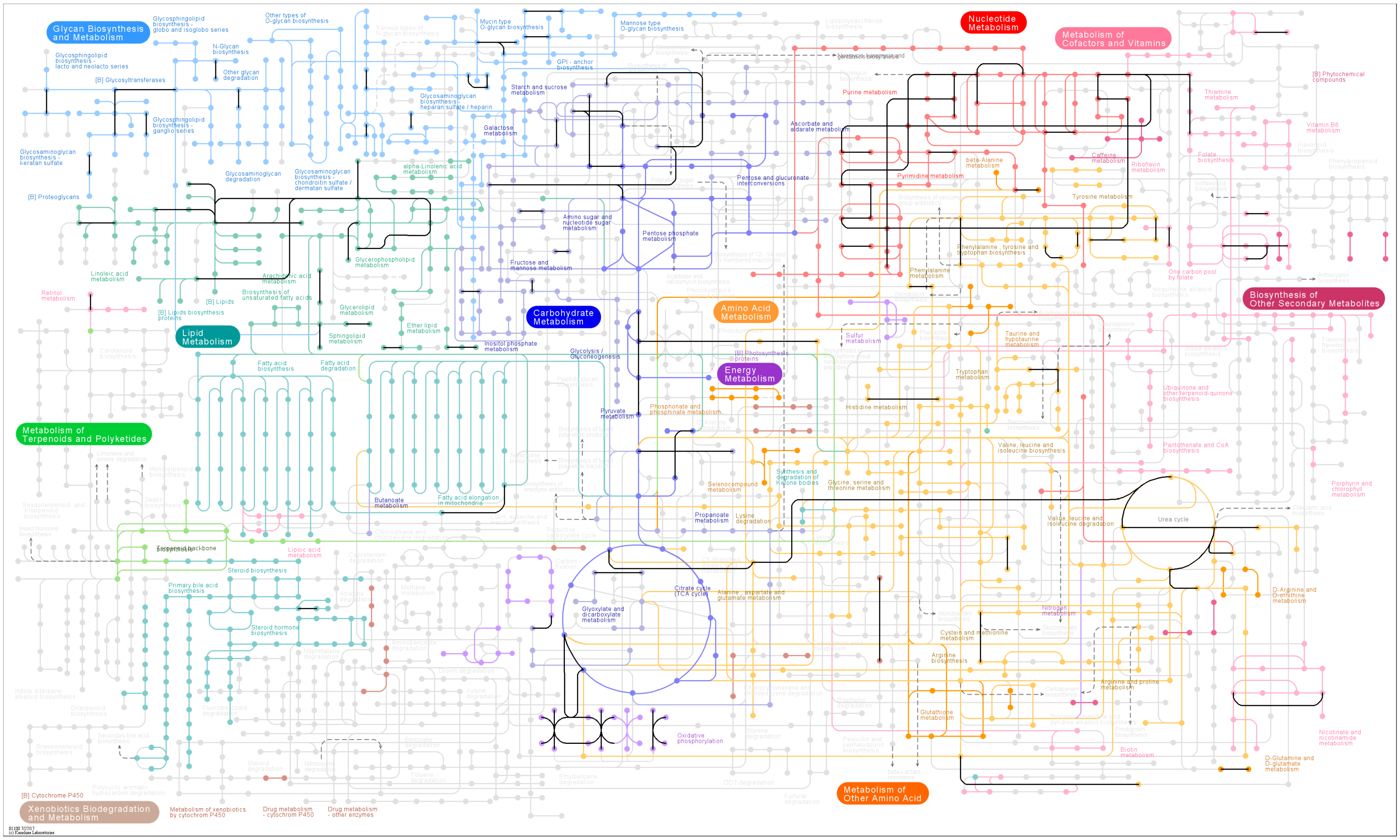

b LD2 to LD3 Down

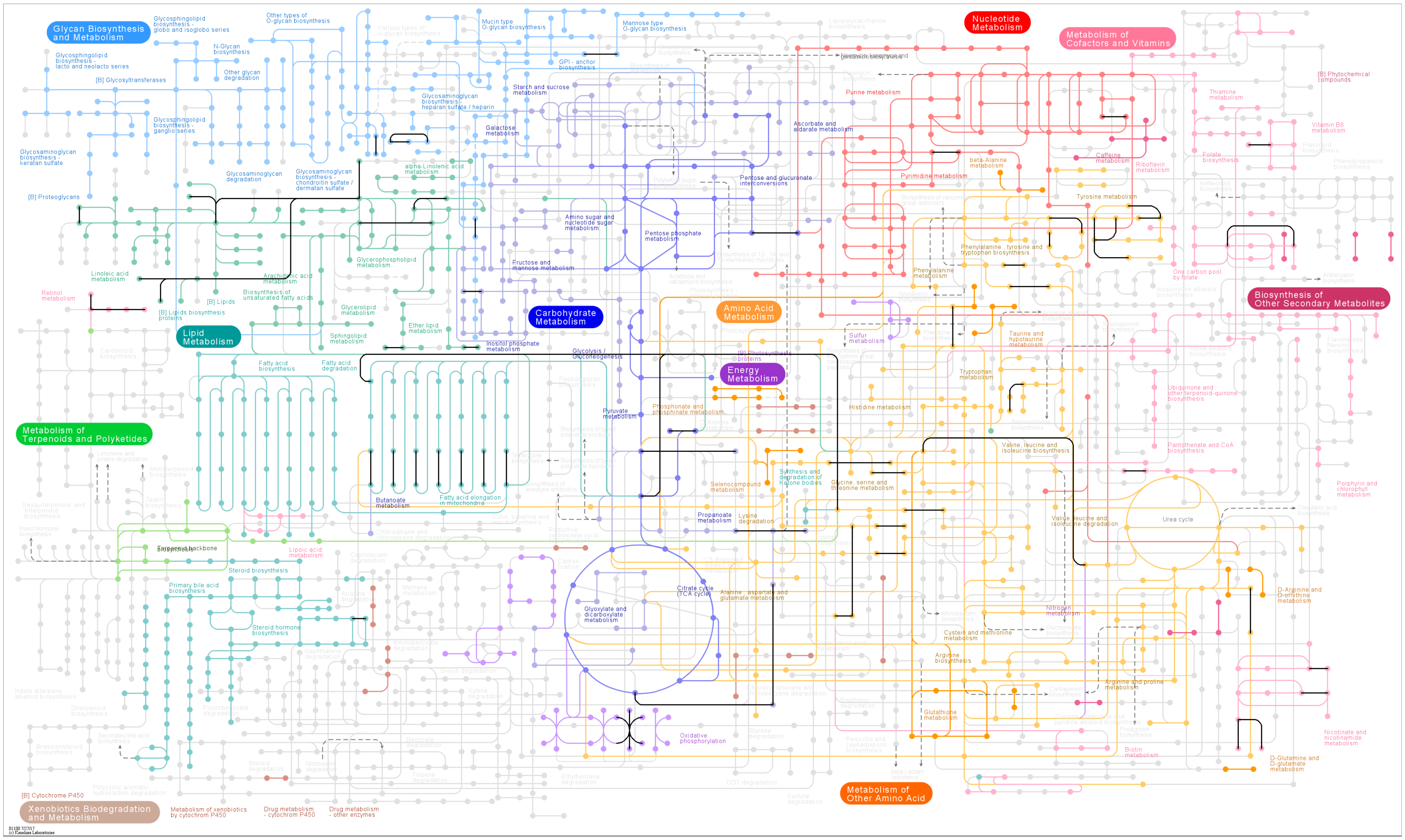

c SD2 to SD3 Up

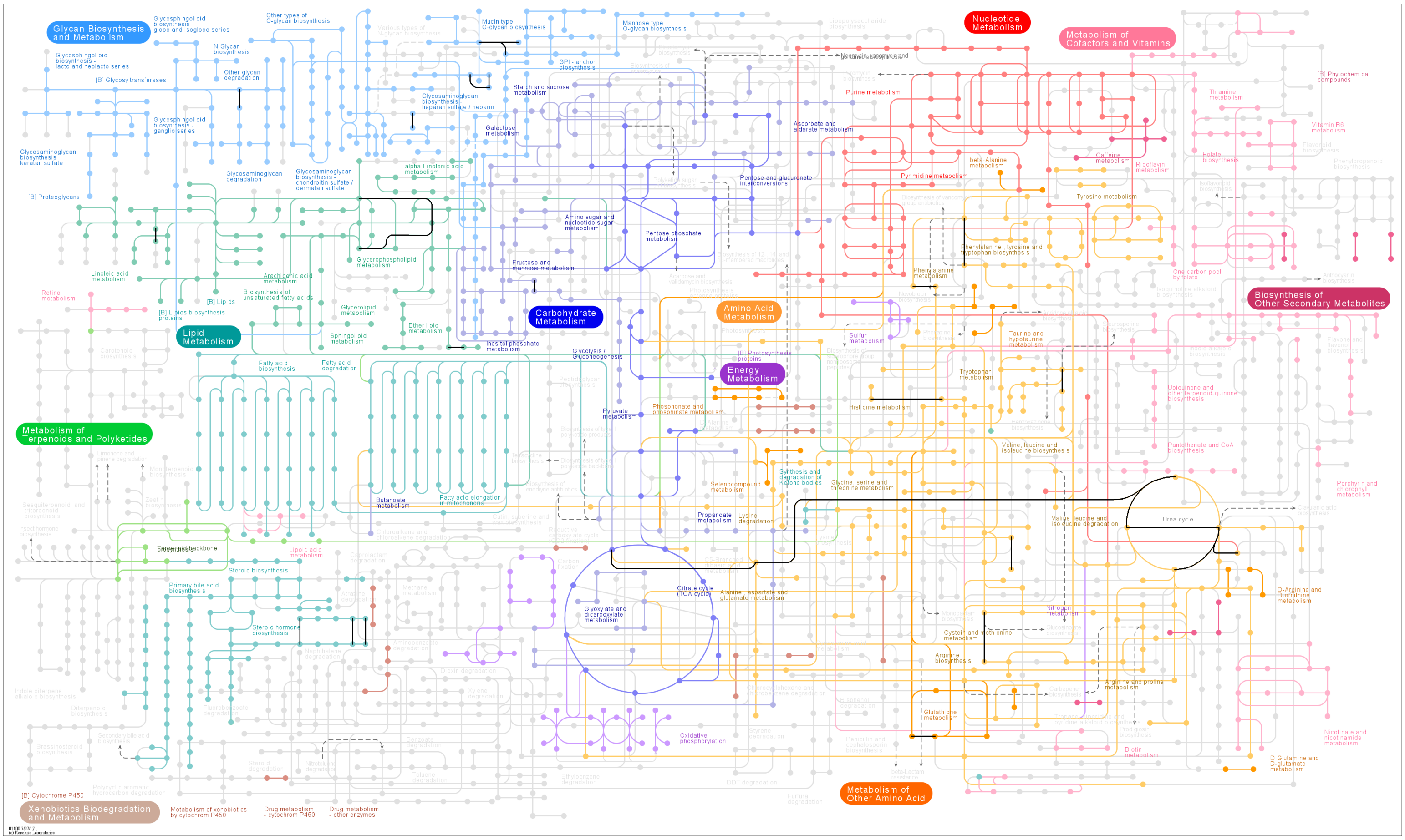

d SD2 to SD3 Down

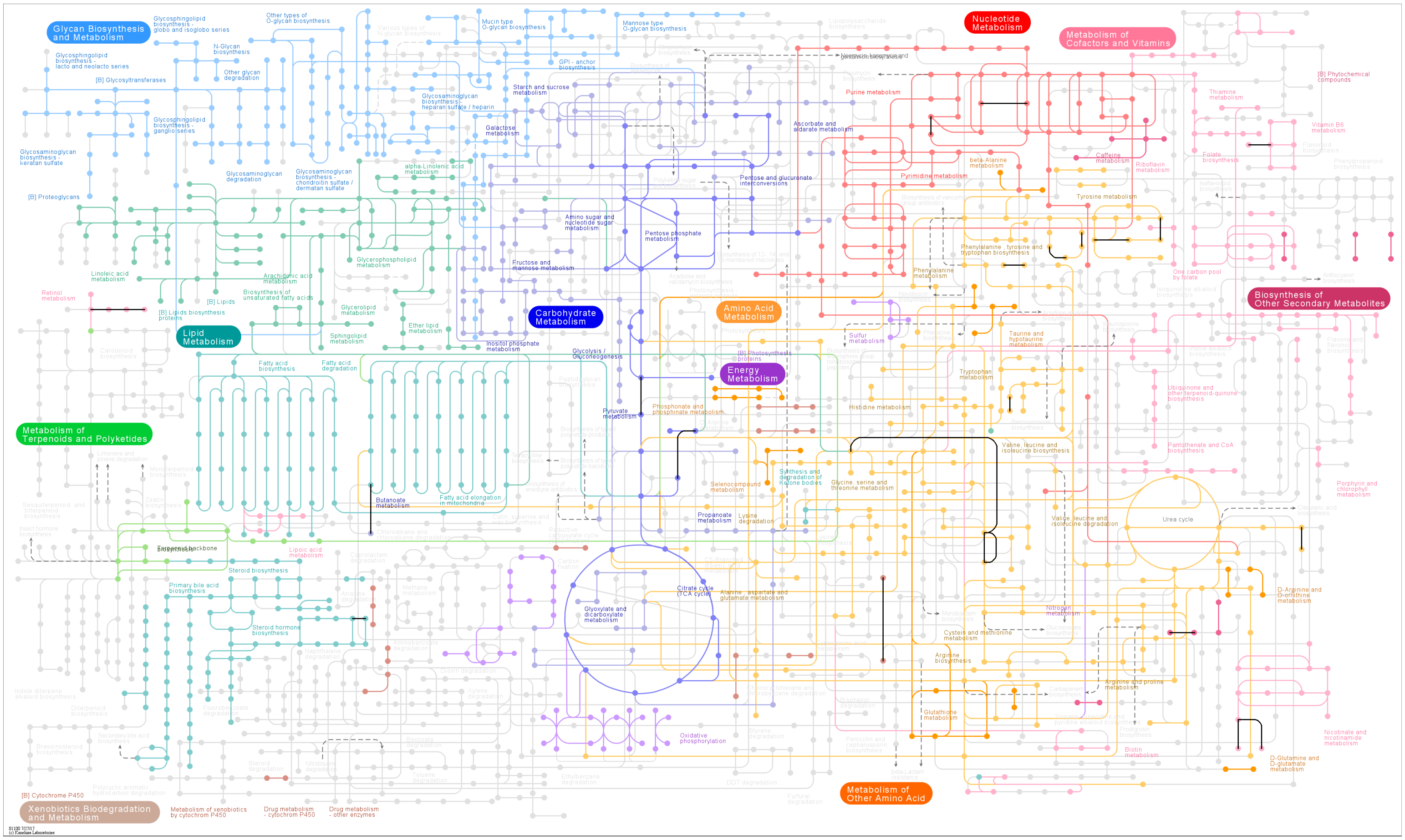

e LD2 Enriched

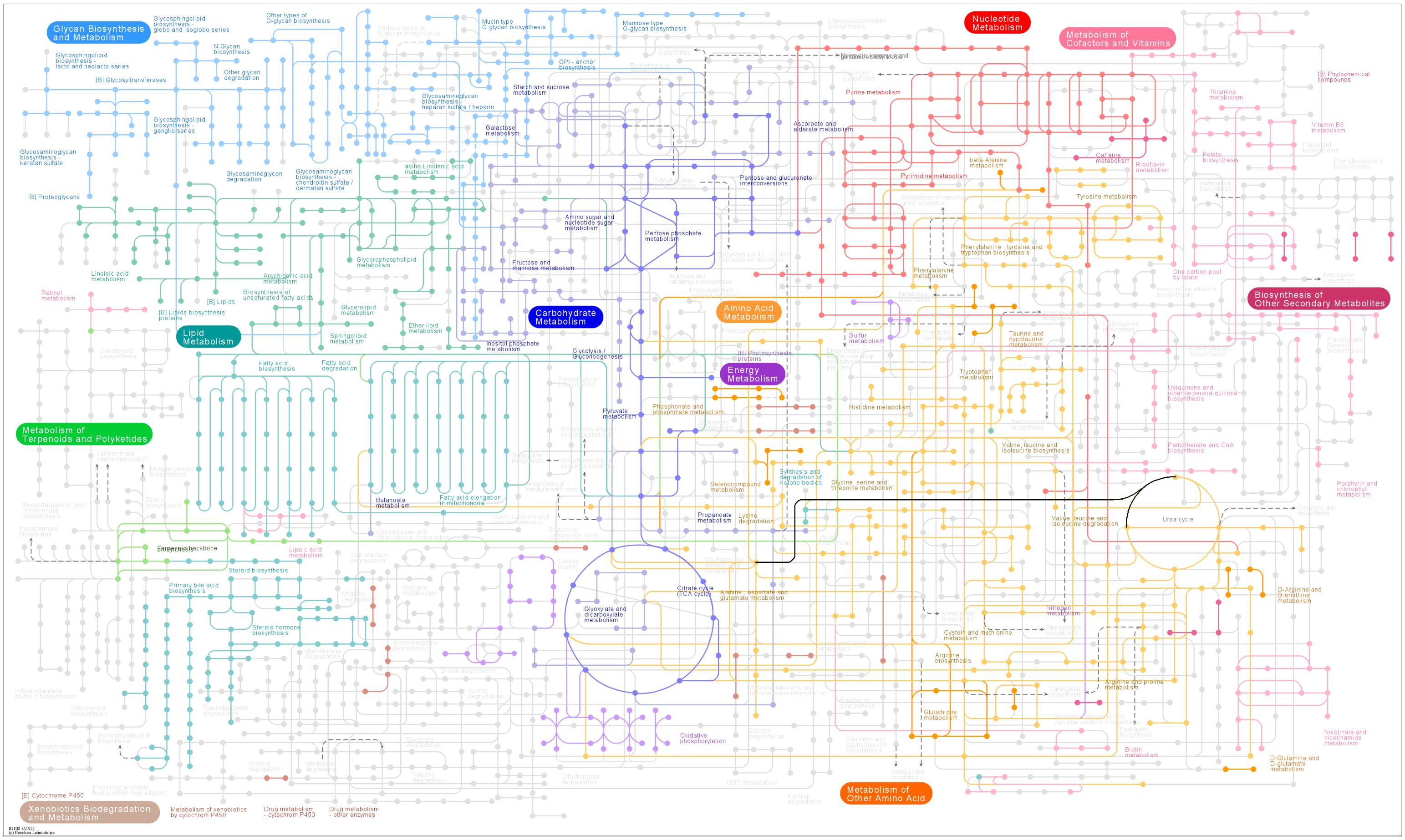

f LD3 Enriched

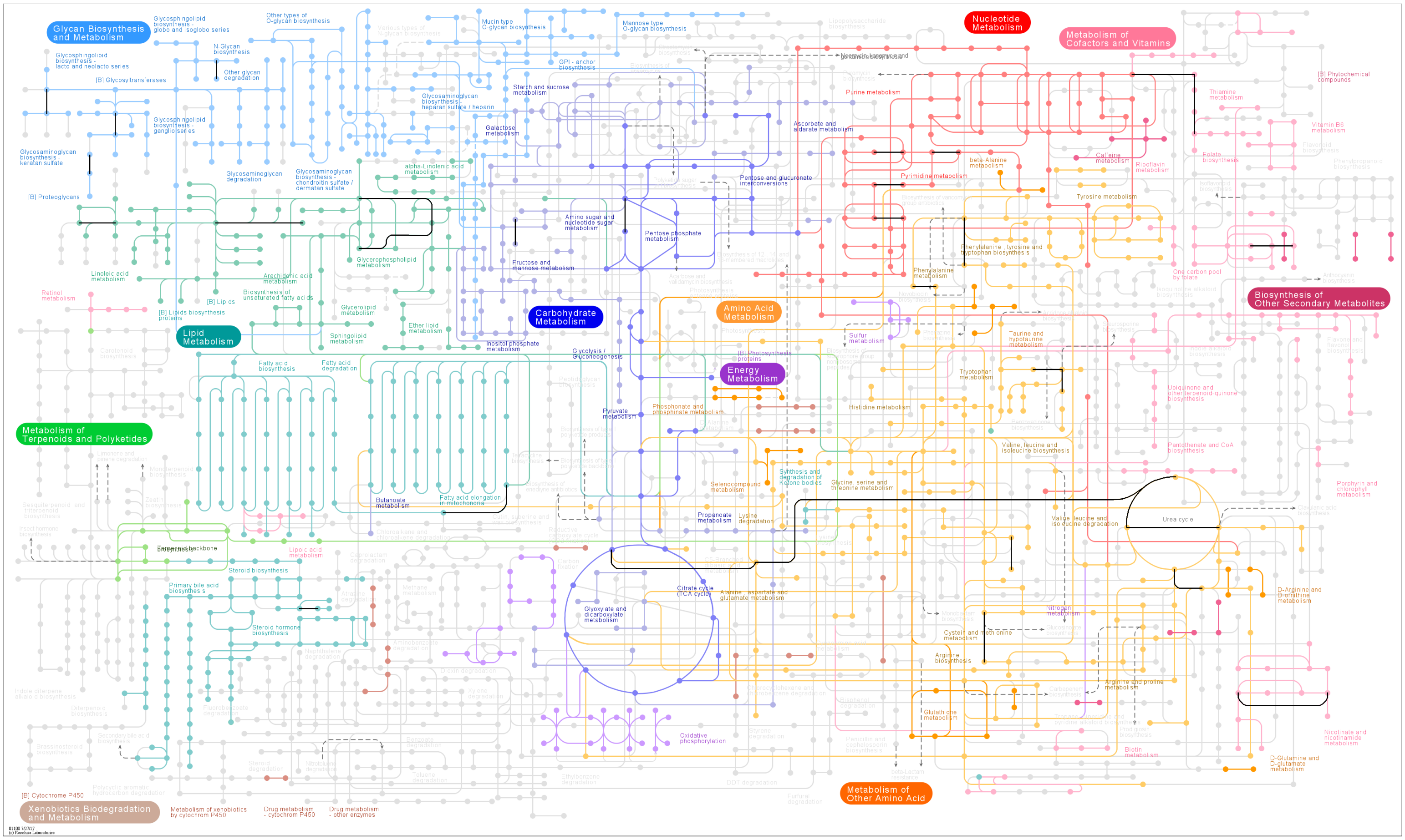

g SD2 Enriched

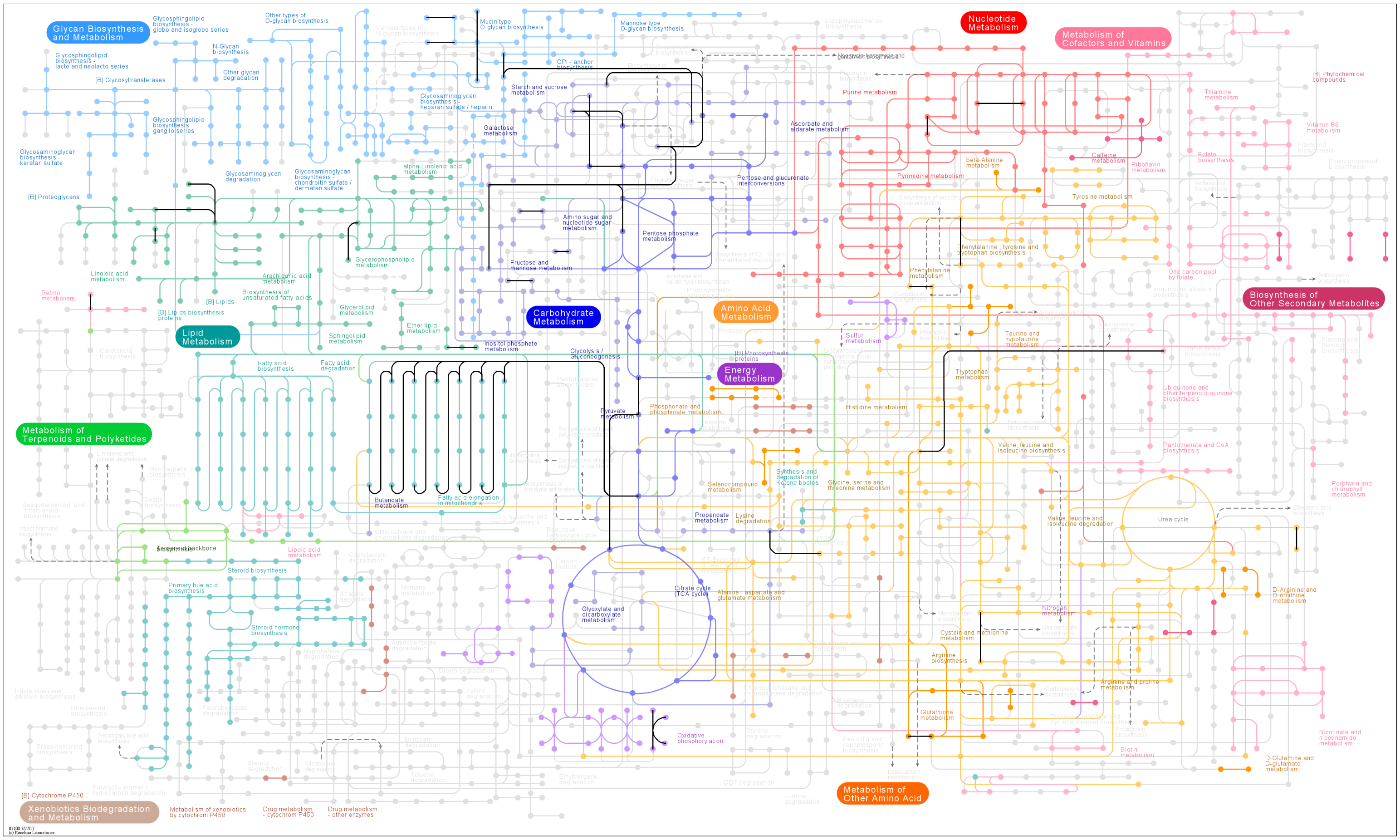

h SD3 Enriched

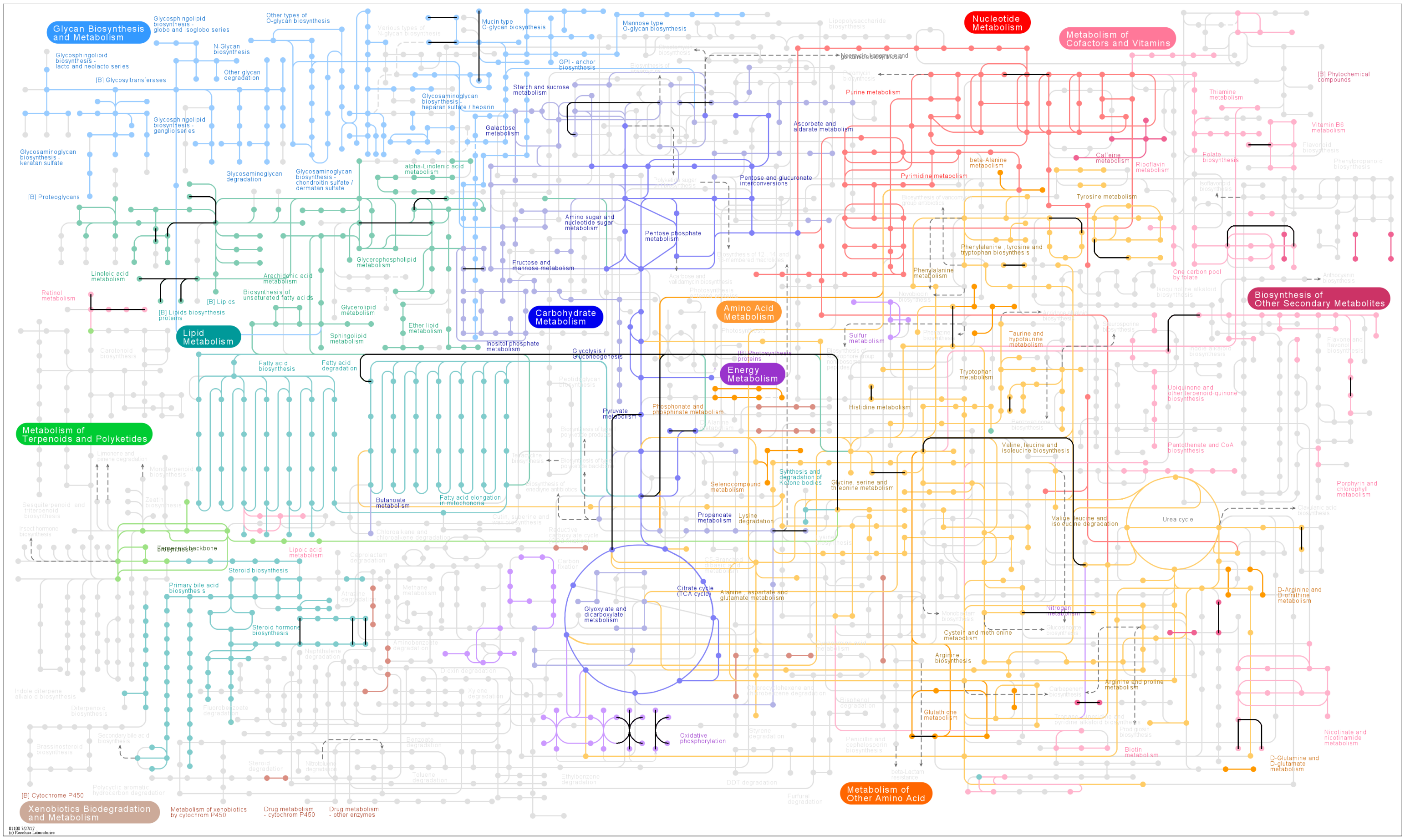

Supplementary Figure S7. Overlay of host differentially expressed genes identified during the chemotherapeutic and steroid models of invasive pulmonary aspergillosis onto the KEGG primary metabolic pathway for mice[36]. The KEGG primary metabolic pathway for mice was overlaid with differentially expressed genes from day 2 to day 3 in the (ab) chemotherapeutic and (cd) steroid model, and statistically enriched genes in the (ef) chemotherapeutic and (gh) steroid model on day 2 and day 3. Differentially expressed genes are colored in black. Chemotherapeutic Model, L; Steroid Model, S; Day 2, D2; Day 3, D3; Up, differentially expressed genes increasing in expression. Down, differentially expressed genes decreasing in expression. Enriched, differentially expressed genes of higher transcript expression for the identified model in comparison to the alternate model for a given day.

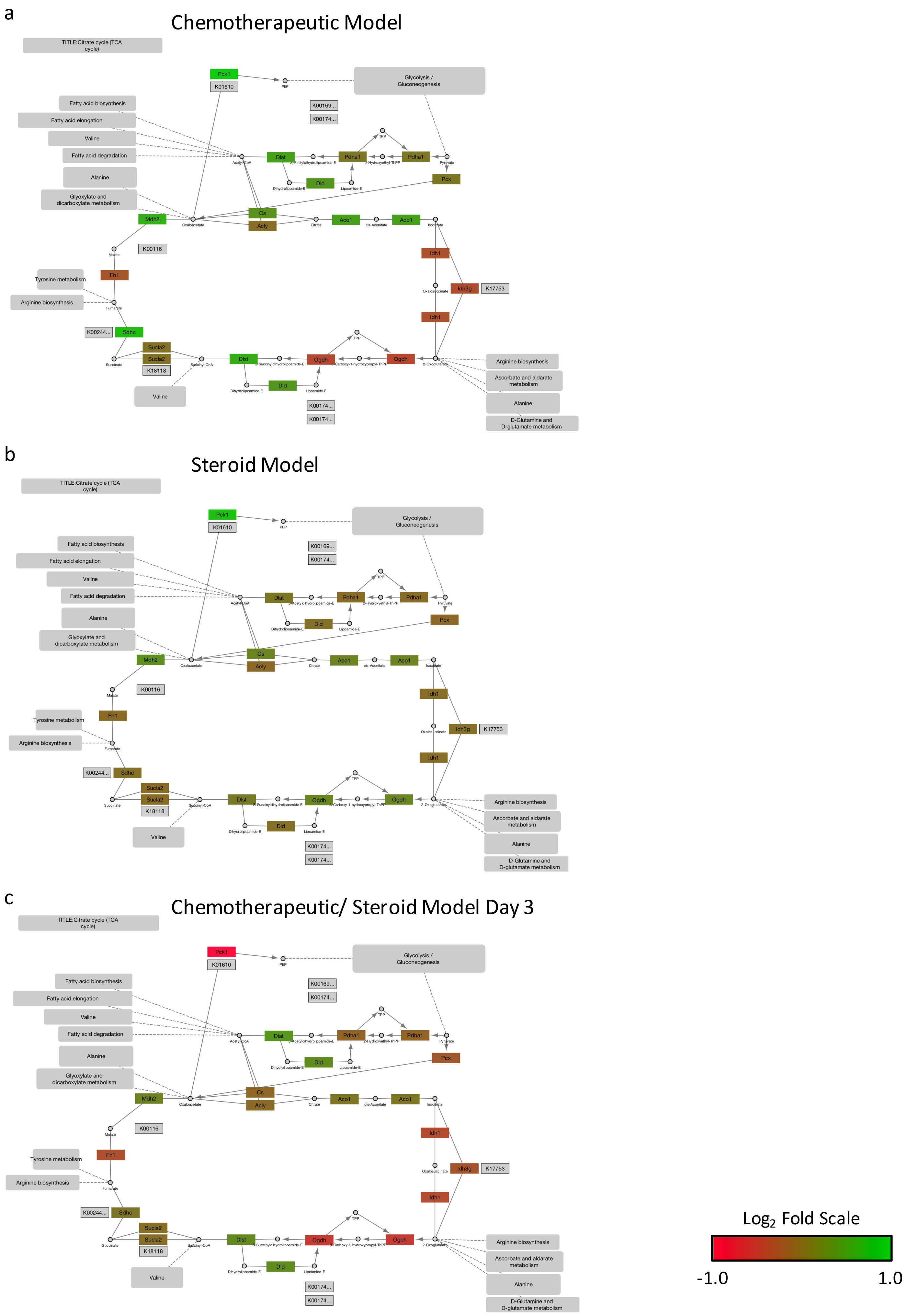
a
Chemotherapeutic Model
b
Steroid Model
c
Chemotherapeutic/ Steroid Model Day 3
TITLE:Citrate cycle (TCA cycle)
Pck1
K01610
PEP
Glycolysis / Gluconeogenesis
K00169...
K00174...
Fatty acid biosynthesis
Fatty acid elongation
Valine
Fatty acid degradation
Alanine
Glyoxylate and dicarboxylate metabolism
Acetyl-CoA
Dlat
S-Acetyldihydrolipoamide-E
Pdha1
2-Hydroxyethyl-ThPP
TPP
Pyruvate
Dihydrolipoamide-E
Dld
Lipoamide-E
Pcx
Mdh2
Oxaloacetate
Cs
Acly
Citrate
Aco1
cis-Aconitate
Isocitrate
Malate
K00116
Idh1
Tyrosine metabolism
Fh1
Oxalosuccinate
Idh3g
K17753
Arginine biosynthesis
Fumarate
K00244...
Sdhc
Sucla2
Succinate
Succinyl-CoA
K18118
Dlst
S-Succinyldihydrolipoamide-E
Ogdh
3-Carboxy-1-hydroxypropyl-ThPP
2-Oxoglutarate
Ascorbate and aldarate metabolism
D-Glutamine and D-glutamate metabolism
Log2 Fold Scale
-1.0
1.0

d Chemotherapeutic Model

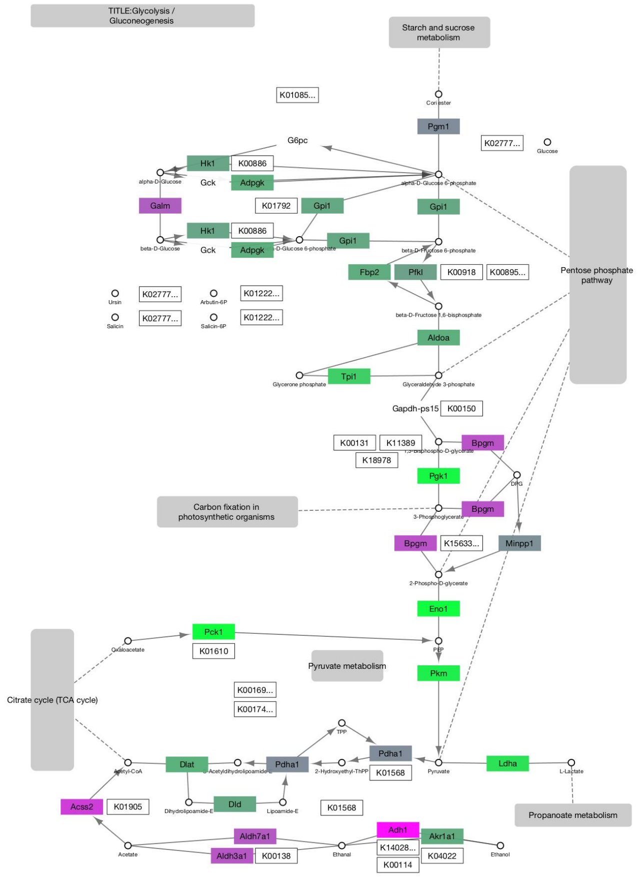

e Steroid Model

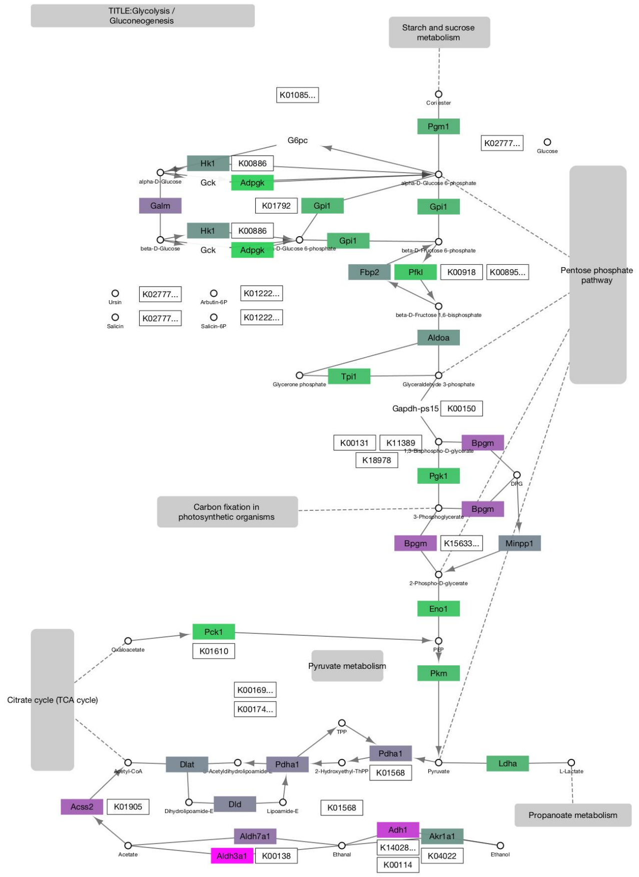

f Chemotherapeutic/ Steroid Model Day 3

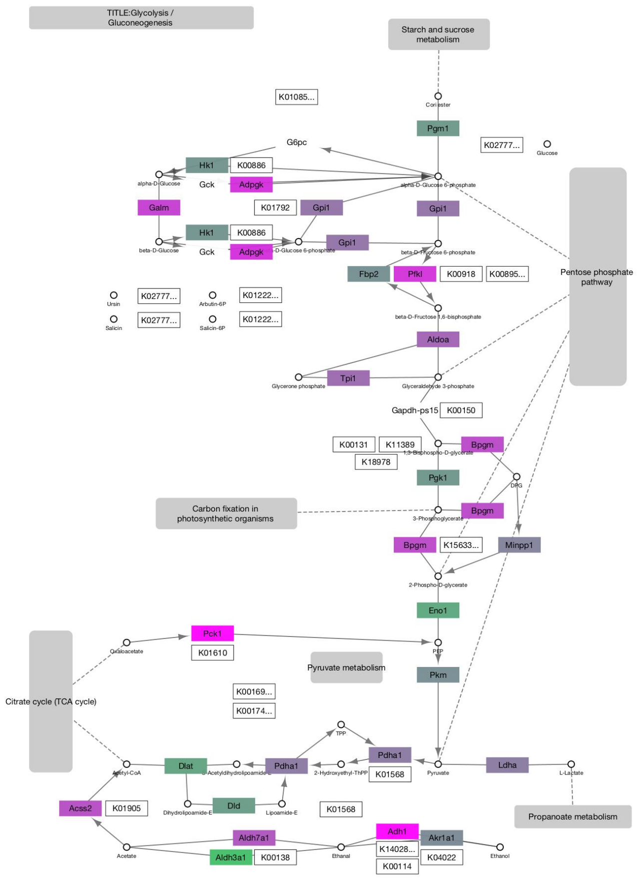

Log_2 Fold Scale

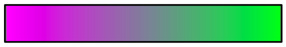

-1.5 1.5

g Chemotherapeutic Model

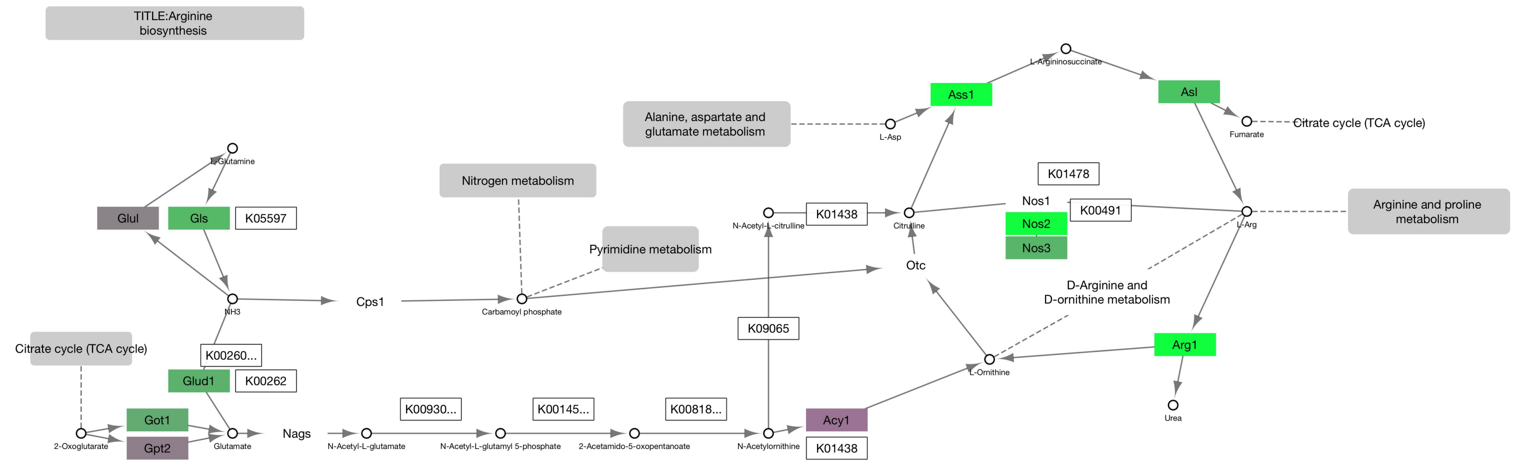

h Steroid Model

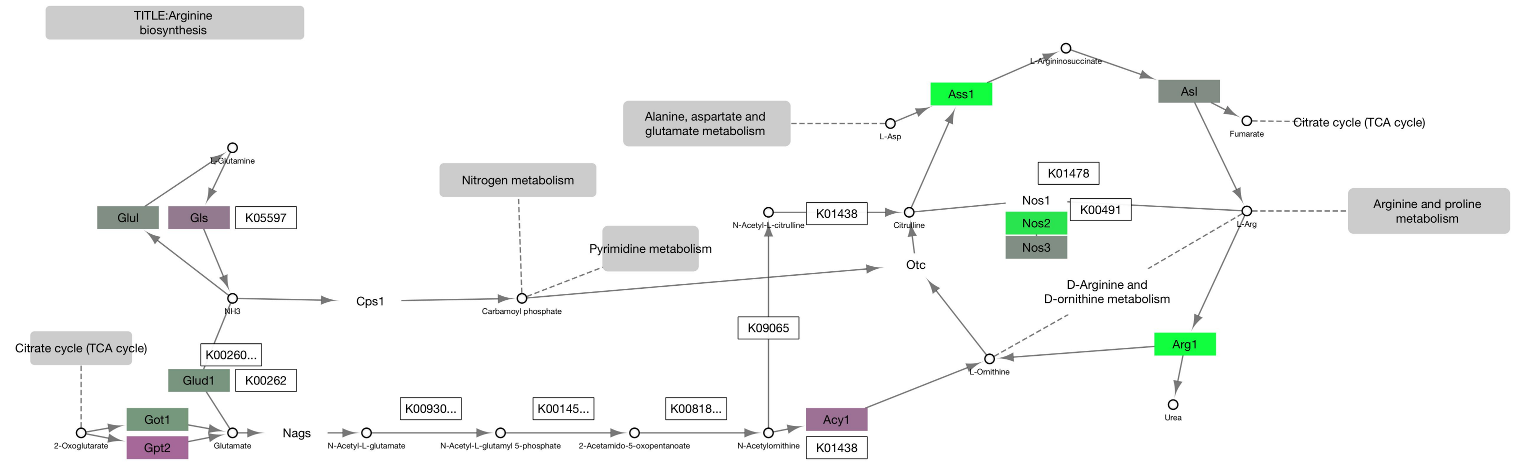

i Chemotherapeutic/ Steroid Model Day 3

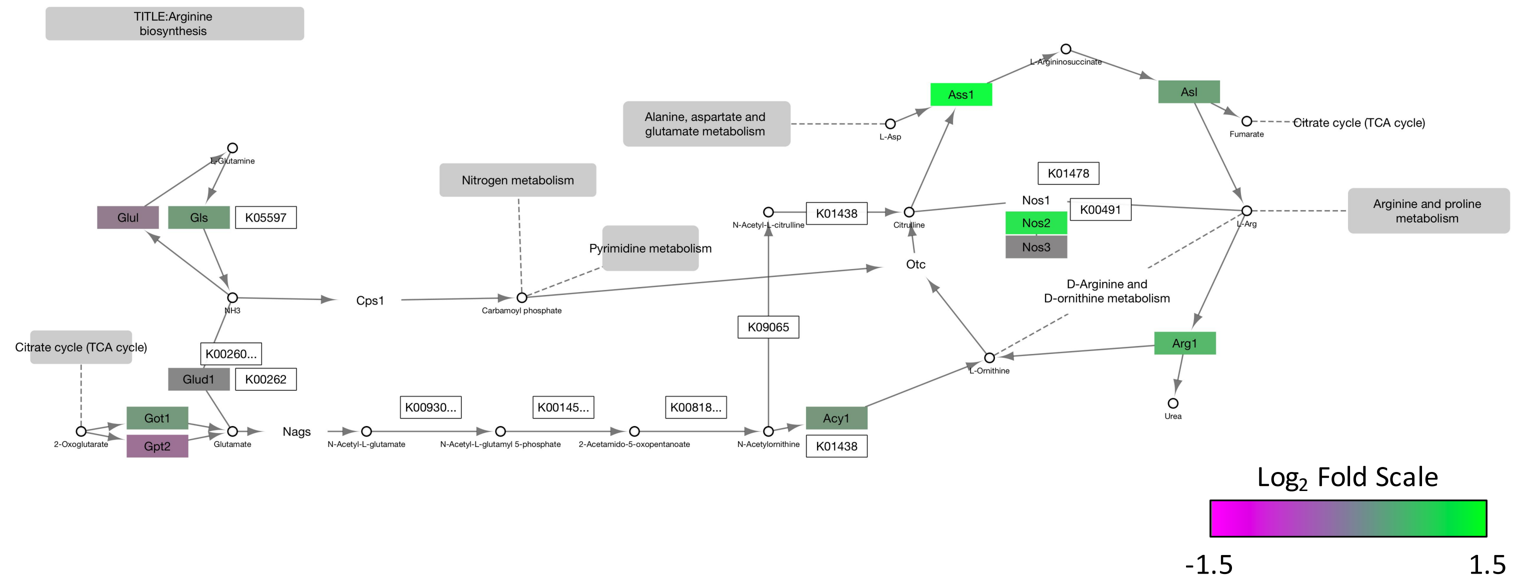

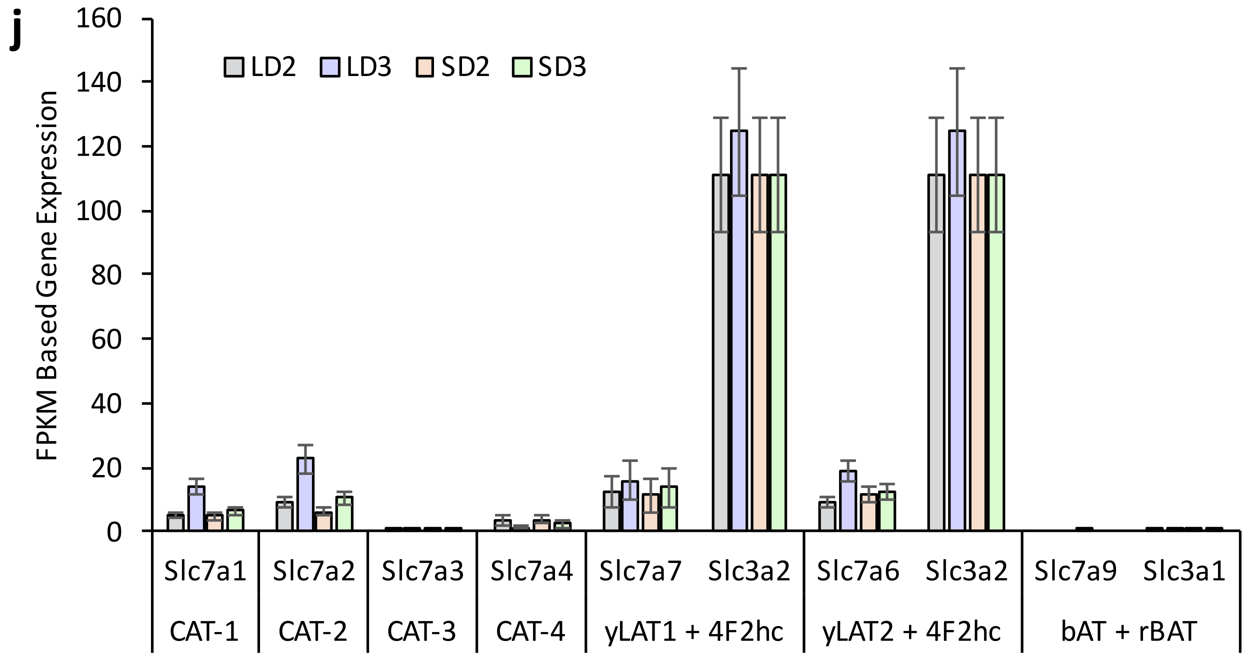

Supplementary Figure S8. Gene expression changes for metabolic pathways during the chemotherapeutic and steroid models of invasive pulmonary aspergillosis from day 2 to day 3 post inoculation. Gene expression was integrated into mouse KEGG based maps[36] for (abc) the citrate acid (TCA) cycle, (def) glycolysis, and (ghi) arginine biosynthesis (urea cycle) using cytoscape[33] and KEGGScape[123]. Genes are colored based on $\log_2$-fold change from day 2 to day 3 for the chemotherapeutic model (adg), steroid model (beh), and gene expression ratio between the chemotherapeutic model and steroid model on day 3 (cfi). White boxes with 'KO' prefixes are genes not present or identified in the mouse version of these pathways. White boxes with gene names are genes below the threshold for expression (FPKM < 1). (j) gene expression (FPKM) of known arginine transporters during the chemotherapeutic and steroid models of invasive pulmonary aspergillosis on day 2 and 3 post inoculation. Steroid Model, S; Chemotherapeutic Model, L; Day 2, D2; Day 3, D3.

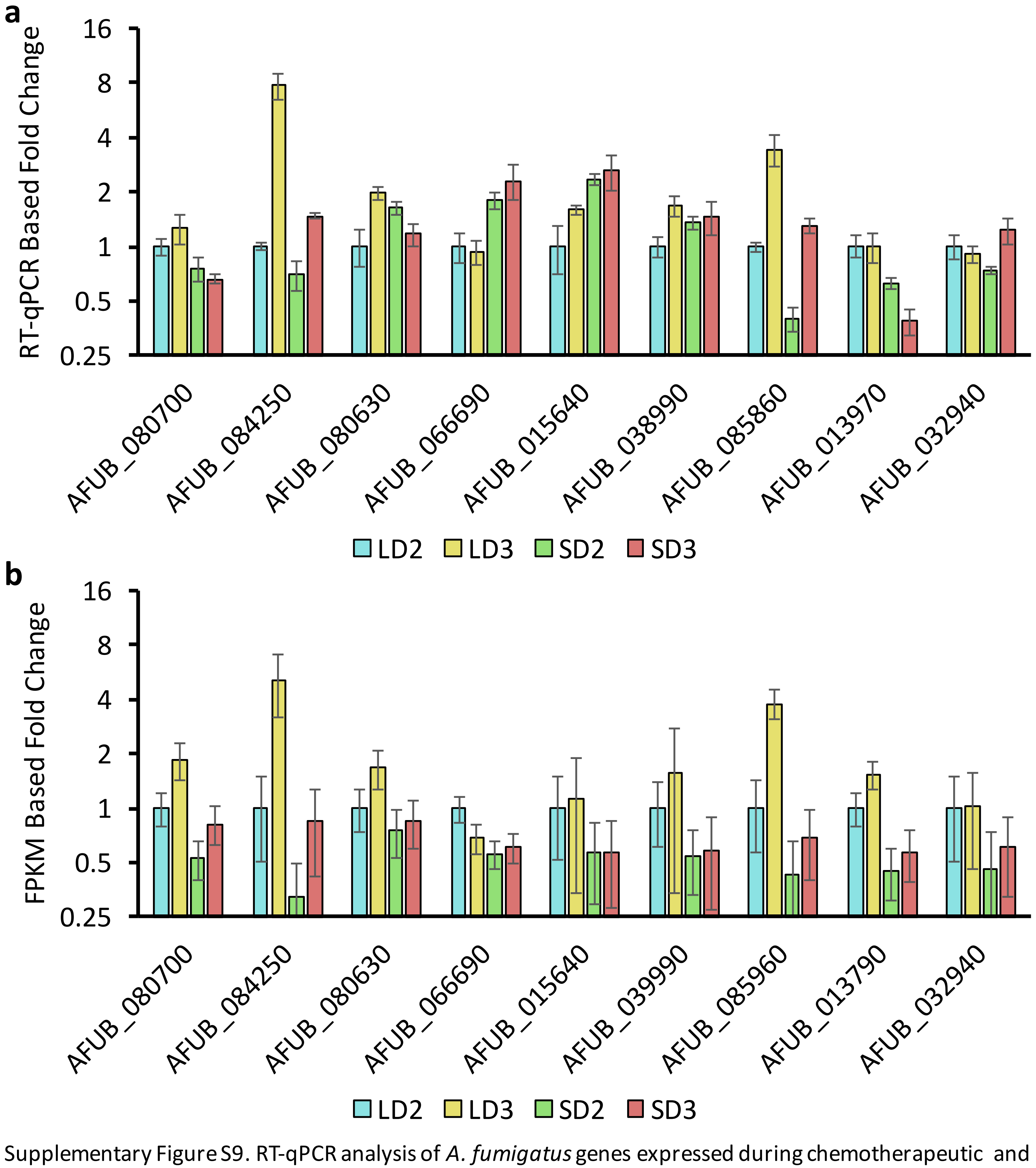

Supplementary Figure S9. RT-qPCR analysis of *A. fumigatus* genes expressed during chemotherapeutic and steroid treatment mouse models of invasive pulmonary aspergillosis on day 2 and 3 post inoculation. Fold change was determined for expressed putative *A. fumigatus* secreted proteins via (a) RT-qPCR analysis and (b) a FPKM based analysis. Fold-change was normalized for RT-qPCR using *β-tubulin* and *TefA,* and presented in relation to LD2 gene expression (comparator) using the *ΔΔCT* method. For FPKM based analysis data is presented in relation to LD2 gene expression (comparator). Steroid Model, S; Chemotherapeutic Model, L; Day 2, D2; Day 3, D3. Standard error of the sample mean is presented. Note the increased FPKM based fold change for all genes in LD3 and the decreased fold change for all genes in SD2 and SD3 in comparison to the RT-qPCR results.

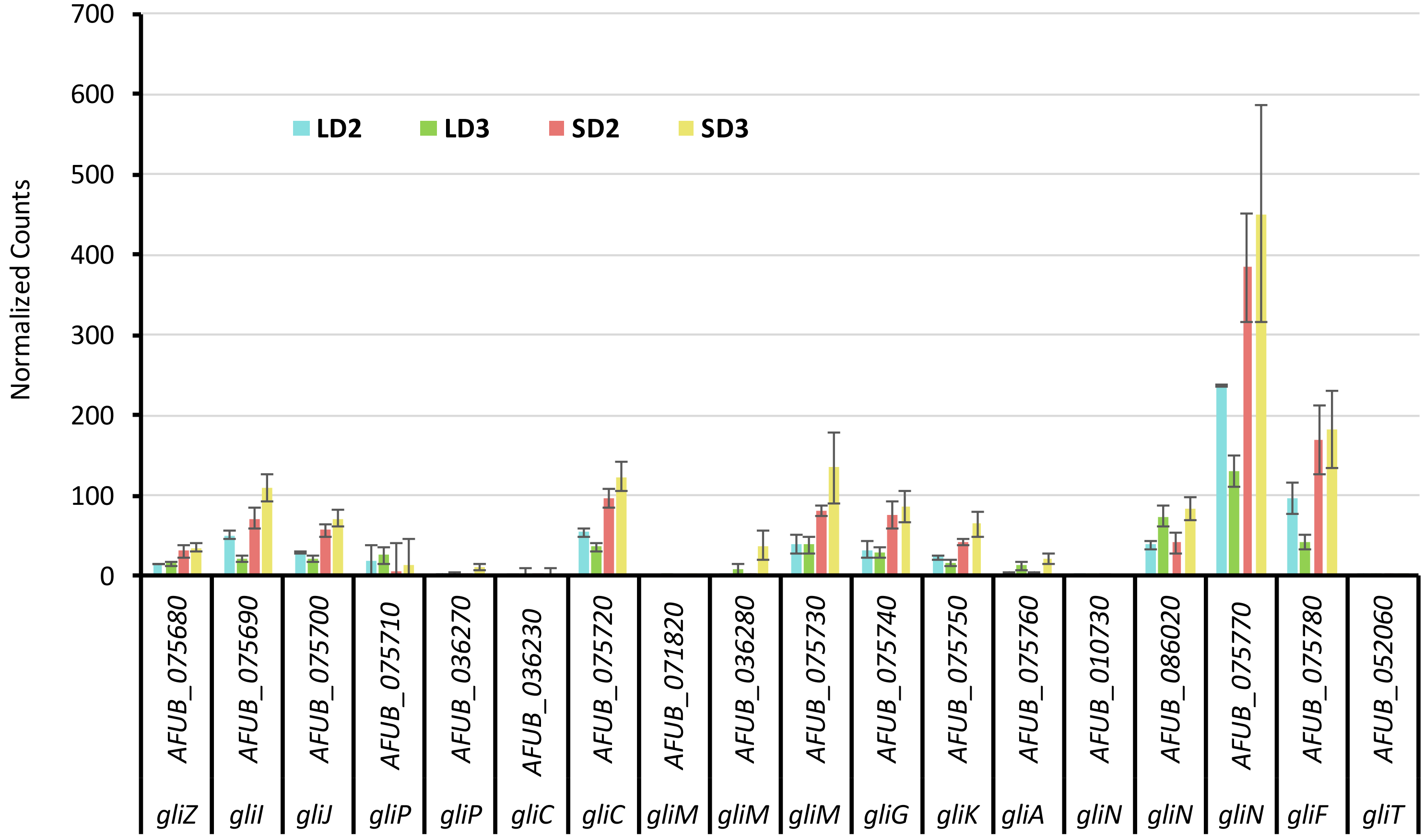

Supplementary Figure S10. Expression level (normalized counts) for *A. fumigatus* genes associated with the gliotoxin biosynthesis cluster during the chemotherapeutic and steroid models of invasive pulmonary aspergillosis on day 2 and 3 post inoculation. Steroid Model, S; Chemotherapeutic Model, L; Day 2, D2; Day 3, D3. Standard error of the sample mean is presented.
